# Harnessing Triplets of TIPS-Anthracene-Based Au(I) Complexes for Photoinduced Electron Transfer and Room-Temperature Phosphorescence

**DOI:** 10.1021/acs.inorgchem.6c01047

**Published:** 2026-08-04

**Authors:** Avik Bhanja, Sree Chithra, Rafael Araujo, Ebin Sebastian, Tomas Edvinsson, Victor Gray

**Affiliations:** † Department of Chemistry−Ångström Laboratory, 8097 Uppsala University, Uppsala 75237, Sweden; ‡ Department of Chemistry and Materials Science, Aalto University, Espoo 02150, Finland; § Department of Materials Science and Engineering, Uppsala University, Uppsala 75237, Sweden; ∥ Department of Physics, Cavendish Laboratory, 2152 University of Cambridge, Cambridge CB3 0HE, U.K.

## Abstract

Excited triplet states are key to many optoelectronic applications due to their long lifetimes and unique spin properties. Triisopropylsilyl (TIPS)-acene derivatives are well-known for their rich triplet-mediated photochemistry. However, metal-functionalized acenes remain scarce, and the influence of metal coordination on triplet-state processes is largely unexplored. Here, we functionalize a TIPS-anthracene backbone with a diphenylphosphine group and report two gold­(I) complexes: monomeric [Au­(TIPS-AntP)­Cl] and dimeric [Au­(TIPS-AntP)_2_OTf]. The excited-state dynamics of the ligands and the complexes were investigated in solutions. Both Au­(I) complexes exhibit nearly quantitative intersystem crossing, efficiently generating triplet excited states. Time-dependent density functional theory (TD-DFT) calculations corroborated the experimental absorption and emission results by providing insights into the energy gaps governing photophysical processes. The role of the Au­(I) in triplet formation was investigated using femtosecond and nanosecond transition absorption spectroscopy. Alongside fluorescence emission, both the ligands and their complexes exhibit room-temperature phosphorescence (RTP), with the Au­(I) complexes displaying enhanced RTP intensity. The photoactive triplet states of the Au­(I) complexes possess sufficiently long lifetimes to enable photoinduced electron transfer to appropriate electron acceptors. Overall, these findings establish Au­(I)-coordinated TIPS acenes as a promising platform for triplet-mediated photochemical and optoelectronic applications.

## Graphical abstract

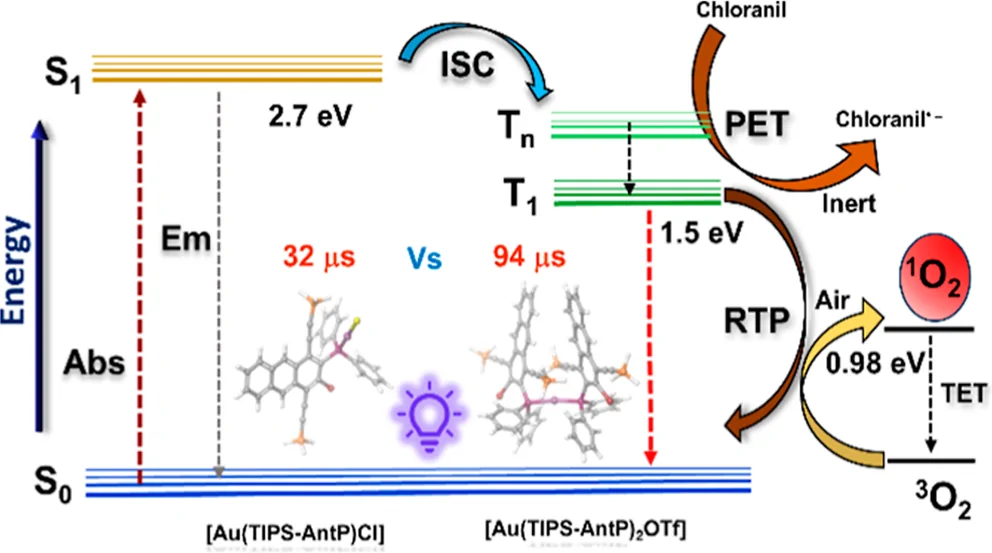

## Introduction

Molecular acenes are a distinctive group of polycyclic aromatic hydrocarbons (PAHs) composed of linearly fused aromatic rings and are well-known for their unique optoelectronic properties.^1–4^ Their extended π-conjugation and planar structures make them suitable candidates for a range of molecular electronic applications, including organic light-emitting diodes (OLEDs),^5,6^ organic photovoltaics (OPVs),^7,8^ triplet–triplet annihilation upconversion (TTA-UC),^9–11^ and photocatalysis.^12^ Among the wide variety of acenes, TIPS-substituted derivatives, particularly TIPS-pentacene and TIPS-tetracene, are widely used for singlet fission (SF) and TTA-UC.^13–17^ However, their relatively low triplet energies (0.8–1.2 eV) limit their suitability for integration into Si-based devices.^18^ In this context, TIPS-anthracene is an attractive candidate since its triplet energy (∼1.4 eV) lies well above the Si bandgap. As a result, TIPS-anthracene is widely used as a TTA-UC annihilator^19,20^ and as a singlet fission (SF) chromophore.^21,22^ In both cases the TIPS-anthracene triplet state is used to control and harness triplet excitons, either via a fusion or multiplication process.

Triplet excitons with long lifetimes are essential for a variety of emerging photochemical applications such as photodynamic therapy, oxygen sensing, solar energy conversion, photocatalysis, etc.^23–26^ However, their inherently “dark” (nonemissive) nature often makes them challenging to use and study. Yet, under certain circumstances, triplet excitons can recombine radiatively to produce phosphorescence. Achieving and controlling free triplets requires a clear mechanistic understanding of the triplet formation pathways and precise control over radiative and nonradiative decay channels.

Intersystem crossing (ISC) is one of the primary pathways for populating excited triplet states. Although ISC is intrinsically spin-forbidden, the rate can be enhanced through spin–orbit coupling (SOC), enabling efficient conversion from the singlet (S_1_) to triplet (T_*n*_) states. Incorporation of heavy atoms, such as bromine, into organic chromophores is a common strategy to increase SOC and promote ISC.^27–30^ Transition-metal-based compounds have also been getting attention in recent times for triplet-mediated applications.^31^ Among the d-block metals, recent advances in Fe­(III)^32,33^ and Cr­(III)^34^ complexes have demonstrated long-lived photoactive doublet states suitable for doublet-triplet energy transfer (DTET). Gold­(I) complexes, however, remain among the most promising systems for achieving strong SOC and are efficient photosensitizers.^35,36^ In addition, Au­(I) complexes are also notable for their luminescence properties,^37^ sensing applications,^38^ medicinal relevance,^39,40^ and catalytic activity.^41–44^ Aurophilic Au···Au interactions^45–47^ and thermally activated delayed fluorescence (TADF) are two extensively explored topics in Au­(I) chemistry.^48–50^ For TADF-active Au­(I) complexes, a small S_1_–T_1_ energy gap (Δ*E*_ST_) facilitates reverse intersystem crossing (RISC).^51^ On the other hand, in systems with large Δ*E*_ST_ gaps, RISC is suppressed, leading to well-defined, long-lived triplet states that can support electron- and energy-transfer processes relevant for photocatalysis, solar harvesting, and energy storage applications.

In this work, we aim to develop new TIPS-anthracene-based chromophores with efficient triplet generation through ISC and/or SF and large Δ*E*_ST_ gaps and explore their photochemical applications. Toward this end, a TIPS anthracene backbone was functionalized with a diphenylphosphine (−PPh_2_) moiety to enable coordination with heavy metal ions. These structures allowed for a systematic investigation of the excited-state dynamics and triplet-state behavior upon metal ion coordination.^52,53^ Tuning the incorporated metal ion and organic chromophore can offer valuable photophysical insights, including strong NIR triplet emission.^54,55^ With this motivation, we report the synthesis and structural and photophysical characterization of [Au­(TIPS-AntP)­Cl] (compound **H)** and [Au­(TIPS-AntP)_2_OTf] (compound **I**), where TIPS-anthracene chromophores are coordinated to the Au­(I) center in mono- and dimeric fashion, respectively (Scheme 1). We investigate the ability of both complexes to generate triplet states and mediate triplet-driven photoinduced processes, such as singlet oxygen sensitization and photoinduced electron transfer.^56^ Although the dimeric [Au­(TIPS-AntP)_2_OTf] complex could, in principle, facilitate SF based on the known behavior of TIPS-anthracene,^57^ our results show that ISC on the picosecond time scale is the primary mechanism of triplet formation. The influence of metal coordination in the triplet-state lifetime is examined in detail. Phosphorescence arising from the otherwise dark triplet states is observed in both the free ligand and the Au­(I) complexes. Our findings demonstrate that strategic incorporation of heavy atoms not only enhances SOC but also provides precise control over the excited-state dynamics in organic semiconductors. This work thus bridges coordination chemistry and organic semiconductor photophysics, expanding the potential of triplet-based technologies.

**Scheme 1:**
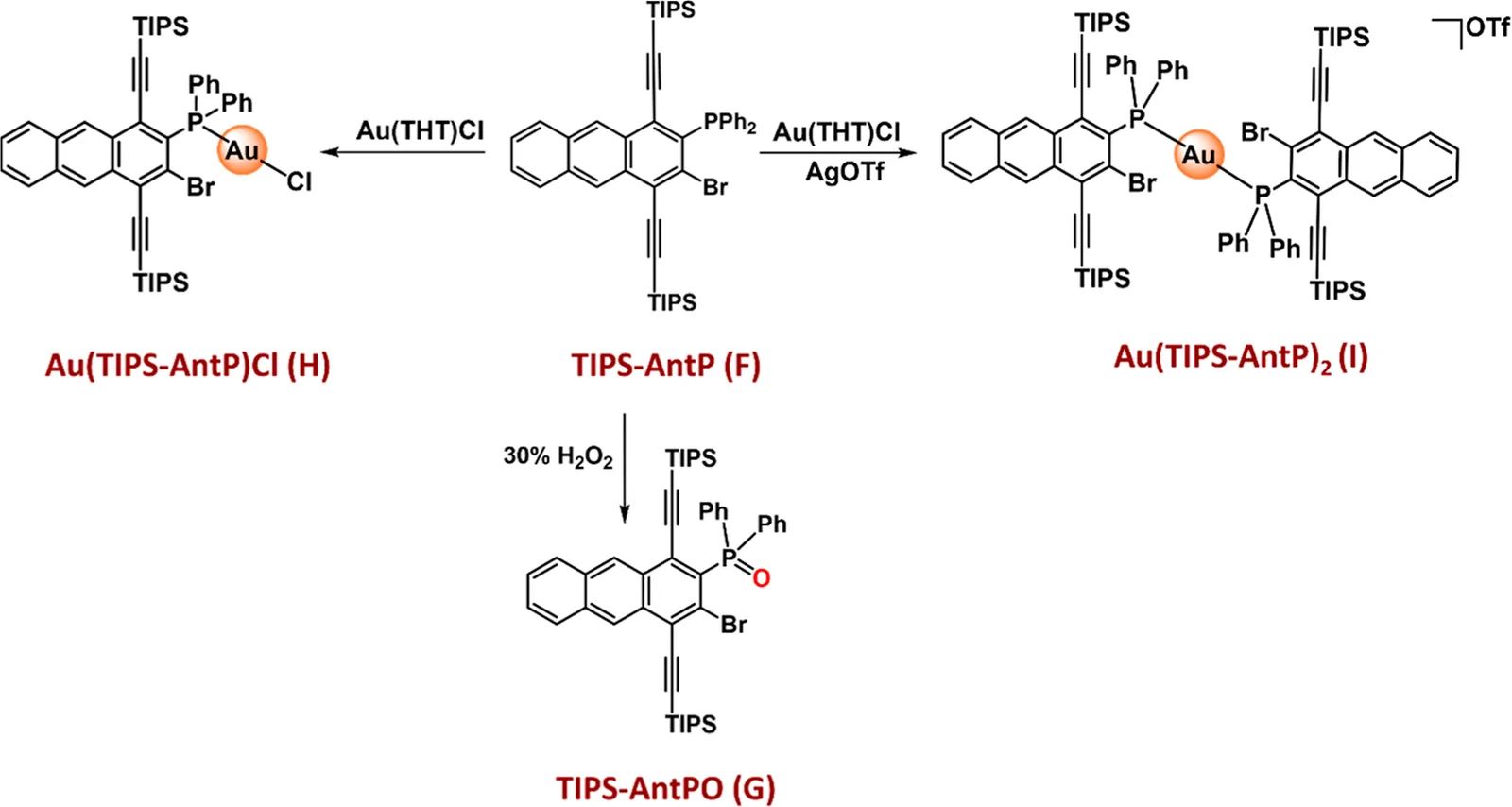
Schematic Illustration of Synthesis of TIPS-Acene-Based Compounds and Its Gold­(I) Complexes

## Results and Discussion

### Synthesis and Characterization

The synthetic protocol of dibromo compound **E** is described in detail in the Supporting Information.^58^ Compound **E** was functionalized using chlorodiphenylphosphine (PPh_2_Cl) to introduce a chelating pocket within the anthracene backbone for metal coordination, following a modified literature procedure, to afford TIPS-AntP (compound **F)**.^59^ Compound **F** was employed further to yield three distinct products. First, compound **F** was oxidized to yield the corresponding phosphine oxide (compound **G**). Compound **F** was also employed for complexation reactions in the presence of Au­(THT)Cl to synthesize the monomeric [Au­(TIPS-AntP)­Cl] **(compound H)** and dimeric [Au­(TIPS-AntP)_2_OTf] (compound **I)** gold complexes, as described in Scheme 1. The unsymmetrical Au­(I) complex bearing one anthracenyl unit (compound **H**) was obtained via a 1:1 stoichiometric reaction between compound **F** and Au­(THT)­Cl under inert conditions. In contrast, the symmetrical Au­(I) complex bearing two anthracenyl units (compound **I**) was achieved via a 2:1:1 stoichiometric reaction involving compound **F**, Au­(THT)­Cl, and AgOTf under an inert atmosphere.

Successful formation of the metal complexes and their precursors was confirmed by ^1^H, ^13^C, and ^31^P NMR spectroscopy (Figures S1–S19). The MALDI-FTICR mass spectrometry further validates the formation of both gold complexes, yielding *m*/*z* = 1034.23 for [Au­(TIPS-AntP)­Cl] and *m*/*z* = 1800.58 for [Au­(TIPS-AntP)_2_OTf] (Figures S20 and S21). Single crystals of compounds **G** and **H**, suitable for X-ray diffraction analysis, were successfully grown by the slow diffusion of diethyl ether into dichloromethane solutions of the compounds at room temperature (Figure 1). The crystal data and the structure refinement parameters are provided in Table S1 in the Supporting Information.

**Figure 1:**
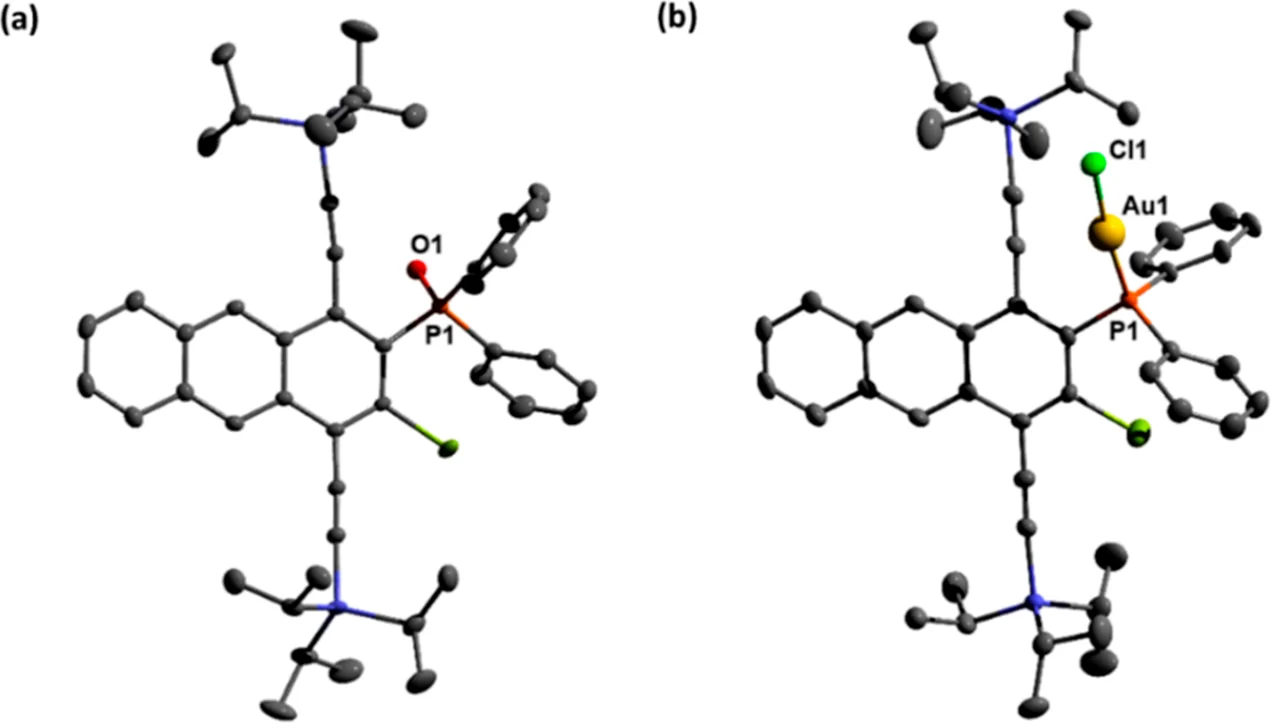
Molecular structures obtained from single-crystal X-ray diffraction of (a) TIPS-AntPO and (b) [Au­(TIPS-AntP)­Cl]. Color code: C, gray; Si, blue; Br, lime; O, red; Cl, green; P, orange; and Au, yellow. Thermal ellipsoids at 50% probability and H atoms were omitted for clarity.

For [Au­(TIPS-AntP)­Cl], charge neutrality is maintained through the coordination of a chloride ion to the Au­(I) center. The Au­(I) center adopts an almost linear coordination geometry with a slight distortion, as reflected in the P–Au–Cl angle of 174.56(5)°. For the dimeric complex **I,** ([Au­(TIPS-AntP)_2_OTf), diffraction-quality single crystals could not be obtained. Nevertheless, its molecular structure was optimized using density functional theory (DFT), and its electronic excitation energies were evaluated using time-dependent DFT (TD-DFT) calculations. Compound **I** is a cationic Au­(I) complex, as the second TIPS-AntP unit coordinates through displacement of the terminal chloride ligand, with countercharge balanced by a trifluoromethanesulfonate (OTf^–^) anion. The crystal packing of both TIPS-AntPO and [Au­(TIPS-AntP)­Cl] is significantly influenced by the bulky -PPh_2_ group, resulting in week π···π interactions between the two adjacent anthracene units (Figures S22 and S23).

### Photophysical Properties

The UV–vis absorption and emission spectra of all the compounds were recorded in toluene at room temperature. The sharp, structured bands observed in the absorption spectra arise primarily from intraligand π–π* electronic transitions (Figure 2). The UV–vis absorption and emission spectra of the free ligand (TIPS-AntP) and its phosphine oxide analogue (TIPS-AntPO) display similar structural features (Figure S24), each exhibiting a strong absorption band at 418 nm. Upon metal coordination, this band undergoes a red shift of 13 nm in the monomeric Au­(I) complex [Au­(TIPS-AntP)­Cl], whereas the dimeric complex [Au­(TIPS-AntP)_2_OTf] shows only a minimal change in the absorption wavelength. The slight blue shift observed for the dimeric compound relative to the monomer, together with the reduced intensity of the 0–0 vibronic peak, suggests weak electronic coupling between the closely spaced anthracene chromophores, coupled by the Au­(I) center in a face-to-face arrangement, consistent with H-aggregate behavior.^60^ The small magnitude of these changes indicates that the coupling is limited, in agreement with the largely monomer-like emission. Such interactions are also supported by the optimized structures from DFT (vide infra). The absorption and emission maxima for all compounds are summarized in Table 1.

**Figure 2:**
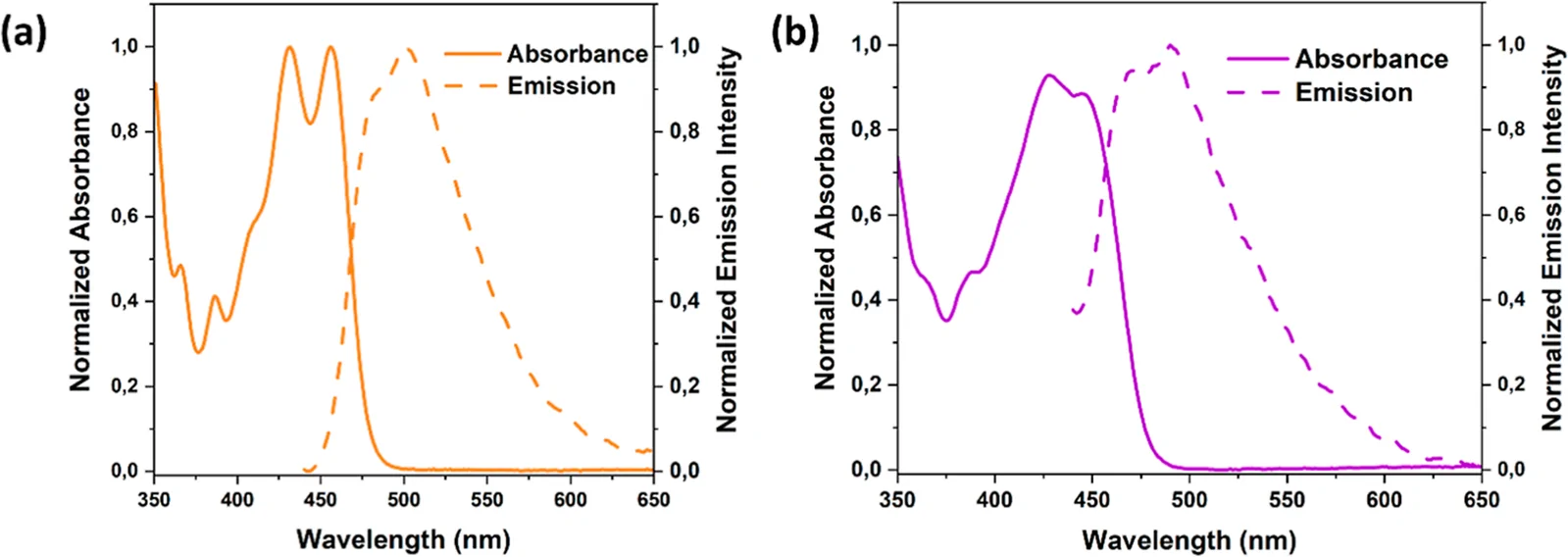
UV–vis absorption (solid) and emission (dashed) spectra of (a) [Au­(TIPS-AntP)­Cl] and (b) [Au­(TIPS-AntP)_2_OTf] recorded in toluene at RT. The excitation wavelength (λ_exc_) was 430 nm.

**Table 1:** Experimental Values of Absorption, Emission, and Phosphorescence Maxima, Fluorescence Quantum Yield, and Singlet (τ_singlet_) and Triplet Lifetimes (τ_triplet_) of the Free Ligands and Its Au­(I) Complexes in Degassed Toluene at 293 K

compound	absorption λ_max_ (nm)	fluorescence emission λ_max_ (nm)	phosphorescence emission λ_max_ (nm)	Φ_Fluo_ [%]	τ_singlet_ [ps]	τ_triplet_ [μs]	*k*_r_ [s^–1^]	*k*_nr_ [s^–1^]	Φ_ISC_ [%]
**TIPS-AntP**	418	484	783	0.45 ± 0.2	22	86	2.1 × 10^8^	4.5 × 10^10^	99.4
**TIPS-AntPO**	423	485	790	7.18 ± 0.4	92	48	7.7 × 10^8^	1.0 × 10^10^	92.9
**[Au(TIPS-AntP)Cl]**	431	498	800	1.14 ± 0.1	37	32.5	2.9 × 10^8^	2.5 × 10^10^	98.9
**[Au(TIPS-AntP)**_**2**_ **OTf]**	428	490	790	2.24 ± 0.1	94	94	2.4 × 10^8^	1.0 × 10^10^	97.7

The absorption and emission spectra of both the Au­(I) complexes were further recorded in degassed organic solvents with increasing polarity, specifically toluene, dichloromethane, THF, and benzonitrile. Both [Au­(TIPS-AntP)­Cl] and [Au­(TIPS-AntP)_2_OTf] exhibit solvatochromic emission shifts, with their emission maxima red-shifting as the solvent polarity increases from toluene to benzonitrile. Specifically, for [Au­(TIPS-AntP)­Cl], the emission maximum appears at 500 nm in toluene and is red-shifted by 17 nm in benzonitrile. In the case of the dimeric complex [Au­(TIPS-AntP)_2_OTf], the emission maxima shift from 490 nm in toluene to 513 nm in benzonitrile. Notably, the emission intensity increased with solvent polarity for the monomeric complex [Au­(TIPS-AntP)­Cl], while the cationic Au­(I) dimer [Au­(TIPS-AntP)_2_OTf] displays negligible changes in emission intensity across solvents (Figure S25). These observations suggest the presence of a polarized excited state in both complexes, yet nonradiative decay pathways are reduced only in the monomeric complex. Fluorescence quantum yields (**Φ**_Fluo_) were determined in toluene using 9,10-bis­(phenylethynyl)­anthracene (BPEA) as a reference. As shown in Table 1, the free ligand TIPS-AntP (compound **F**) exhibits the lowest quantum yield (0.45%), whereas the corresponding phosphine oxide analogue TIPS-AntPO (compound **G**) shows the highest value (7.18%) (Figure S26). Among the Au­(I) complexes, the dimeric [Au­(TIPS-AntP)_2_OTf] complex exhibits a higher Φ_Fluo_ (2.24%) compared to the monomeric complex [Au­(TIPS-AntP)­Cl] (1.14%). The overall low Φ_Fluo_ values and fast triplet formation (vide infra) suggest rapid ISC. Indeed, the fluorescence decay was too fast to be accurately resolved using traditional time-correlated single-photon counting (TCSPC).

Electronic structure calculations were performed to gain further insights into the optoelectronic properties. In the monomeric Au­(I) complex, the HOMO is predominantly localized on the π-system of the anthracene backbone, whereas the LUMO shifts toward the phosphine/Au coordination site, indicating partial polarization toward the metal center (Figure 3). A similar LUMO distribution is observed for the dimeric [Au­(TIPS-AntP)_2_OTf] complex (Figure S27). Additionally, in the dimer, both HOMO and LUMO exhibit significant electron density across the two anthracene units, suggesting favorable interactions and a stacked arrangement between the chromophores. Such alignment could, in principle, support singlet fission or excimer formation. However, no excimer features were observed experimentally in the emission spectra of [Au­(TIPS-AntP)_2_OTf].

**Figure 3:**
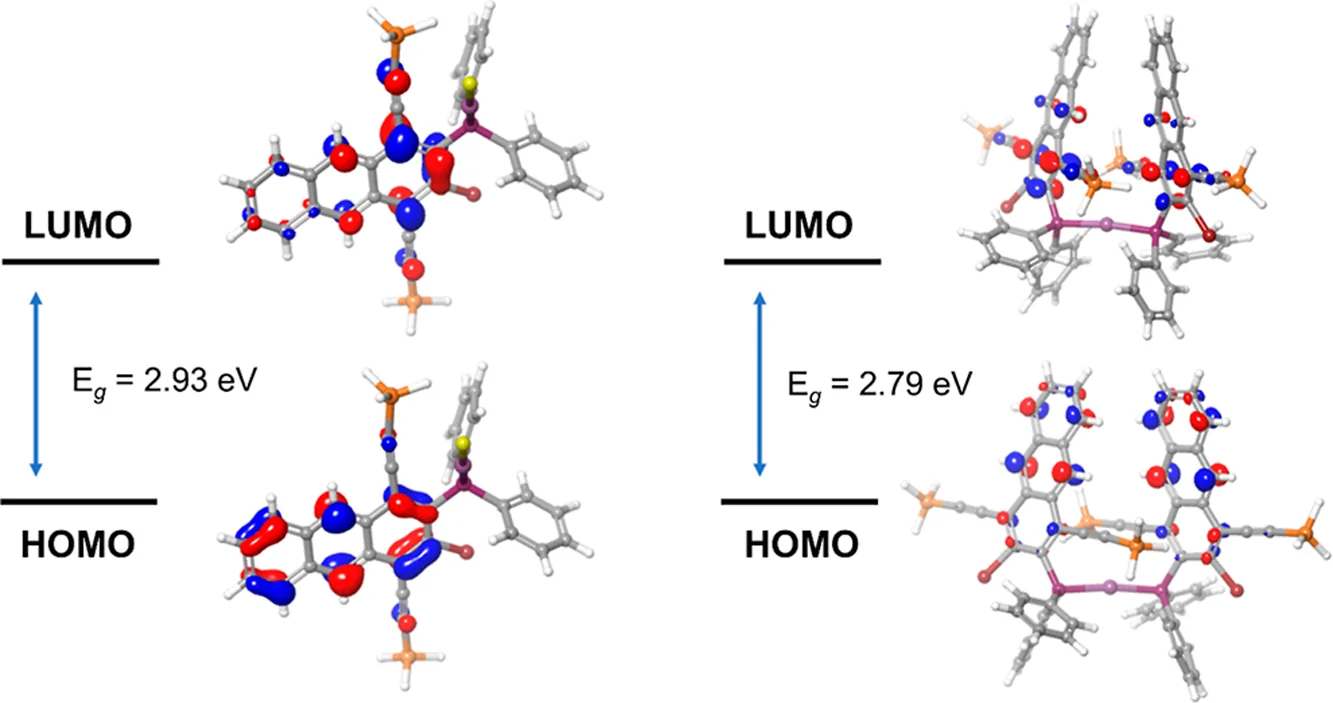
HOMO/LUMO orbitals of (*left*) [Au­(TIPS-AntP)­Cl] and (*right*) [Au­(TIPS-AntP)_2_OTf] complexes.

Energy-level analysis shows that singlet fission is thermodynamically unfavorable for the dimer. Comparison of calculated singlet (2.40 eV) and triplet (1.44 eV) energies indicates an endothermic singlet fission driving force of approximately 0.5 eV (Δ*E* = 2*E*_T_1__ – *E*_S_1__), making the process implausible. TD-DFT calculations slightly underestimate singlet excitation energies for both monomeric and dimeric complexes, as expected for gas-phase B3LYP calculations, while solvent models are needed for comparison with energies from experiments in solutions.^61^ Experimentally, the singlet excitation energy (*E*_00_) for the [Au­(TIPS-AntP)­Cl] and [Au­(TIPS-AntP)_2_OTf] complexes is found to be 2.66 eV (467 nm) and 2.71 eV (457 nm), respectively, in toluene (Figure 2), estimated by normalizing the lowest-energy absorption and emission spectra and determining the point of intersection.

Femtosecond transient absorption (fsTA) spectra were recorded to gain deeper insight into the excited-state dynamics of the compounds. Details of the experimental setups are provided in the Supporting Information. Briefly, transient absorption spectroscopy (TAS) uses two laser pulses: a monochromatic excitation (pump) pulse, followed by a broadband probe pulse. By varying the delay between pump and probe, the differential absorption is recorded as a function of time. All synthesized compounds exhibited similar spectral features and dynamics in TAS, with the principal differences arising only in their temporal evolution. Therefore, we focus the discussion here on the gold complexes (Figure 4), while spectra and analyses of the other compounds are presented in Figures S28 and S29.

**Figure 4:**
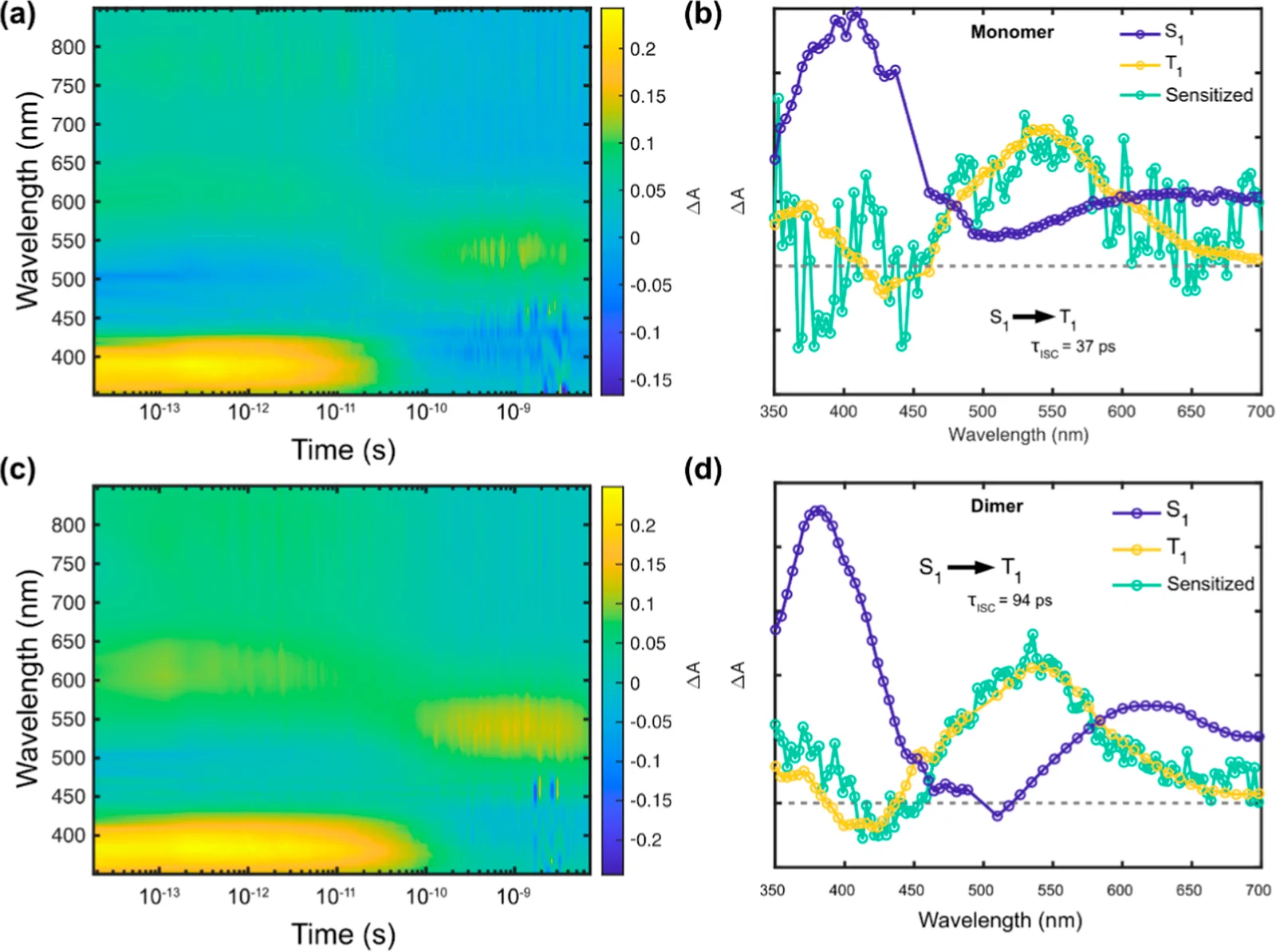
Chirp-corrected 2D fs-transient absorption (fsTA) map of (a) [Au­(TIPS-AntP)­Cl] and (c) [Au­(TIPS-AntP)_2_OTf] upon excitation at 430 nm. (b) Monomer and (d) dimer Au­(I) species-associated spectra of the excited singlet (blue) and triplet (yellow) obtained from global analysis. Sensitized triplet spectra are shown for comparison. The suggested two-state ISC mechanism and fitted ISC time constant τ_ISC_ are also indicated.

A strong photoinduced absorption (PIA) centered around 380 nm was observed for all compounds, along with a weaker, broad PIA spanning 550–750 nm. Ground-state bleach (GSB) and stimulated emission (SE) appear as dips in the PIA in the 450–550 nm region. Spectral evolution occurs over a few hundred picoseconds, where the initial PIAs decay to be replaced by a sharp PIA centered around 550 nm, while the GSB in the 400–450 nm region becomes more pronounced and the SE signal diminishes. The final spectra persist beyond the experimental time window (8 ns) and match the sensitized triplet spectra obtained from nanosecond TAS using PdOEP as a triplet sensitizer (vide infra). The relative amplitudes of singlet and triplet species are comparable for both monomeric and dimeric complexes, indicating that similar numbers of triplets are formed. Thus, the spectral evolution is attributed to rapid ISC, and no experimental evidence of singlet fission is observed in the dimeric complex.

This conclusion is consistent with the energetic alignment between S_1_ and T_3_ predicted from TD-DFT calculations for both complexes, which could facilitate rapid ISC. Furthermore, global analysis of the fsTA data is well described by a simple two-state kinetic model, in which the initial state corresponds to the photoexcited singlet and the final state corresponds to the triplet excited state (Figure 4d). The ISC time constants ( $\tau_{ISC}=\frac{1}{k_{ISC}}$ ) were determined to be 22 ps for TIPS-AntP, 37 ps for [Au­(TIPS-AntP)­Cl], 94 ps for [Au­(TIPS-AntP)_2_OTf], and 92 ps for TIPS-AntPO. Using the measured singlet-state decay rates and fluorescence quantum yields, the radiative (*k*_r_) and nonradiative decay rates (*k*_*n*r_) from the singlet state were calculated (Table 1). Furthermore, assuming that nonradiative decay is dominated by ISC (*k*_*n*r_∼*k*_ISC_), the ISC quantum yields (Φ_ISC_ = *k*_ISC_ */(k*_ISC_ + *k*_*r*_)) were estimated, as listed in Table 1.

Since the triplet lifetime exceeds the temporal window accessible with fsTA, nanosecond transient absorption (nsTA) measurements were used to determine triplet decay dynamics (Figure S30). Upon photoexcitation at 430 nm, a long-lived transient absorption spectrum resembling the final fsTA spectra was observed, which decays on the microsecond time scale. Single-wavelength kinetic traces yield triplet lifetimes of 32 μs for the monomeric [Au­(TIPS-AntP)­Cl] complex, 94 μs for the dimeric [Au­(TIPS-AntP)_2_OTf] complex, 47 μs for the phosphine oxide TIPS-AntPO, and 86 μs for TIPS-AntP (Figure S31).

To further probe triplet dynamics and the influence of SOC, room-temperature phosphorescence (RTP) measurements were conducted. RTP was detected for both Au­(I) complexes under inert conditions. The phosphorescence yield of the complexes was determined to be 0.02% for both the monomer and dimer relative to Ru­(bpy)_3_ in deaerated water (PLQY of 4.2%).^62^ For [Au­(TIPS-AntP)­Cl], phosphorescence appears at 800 nm, while for [Au­(TIPS-AntP)_2_OTf], the emission maximum is at 790 nm (Figure 5). The corresponding triplet energies were estimated from these peak positions to be 1.55 and 1.57 eV, respectively, in good agreement with TD-DFT calculations and supporting the conclusion that singlet fission is energetically unfavorable for [Au­(TIPS-AntP)_2_OTf].

**Figure 5:**
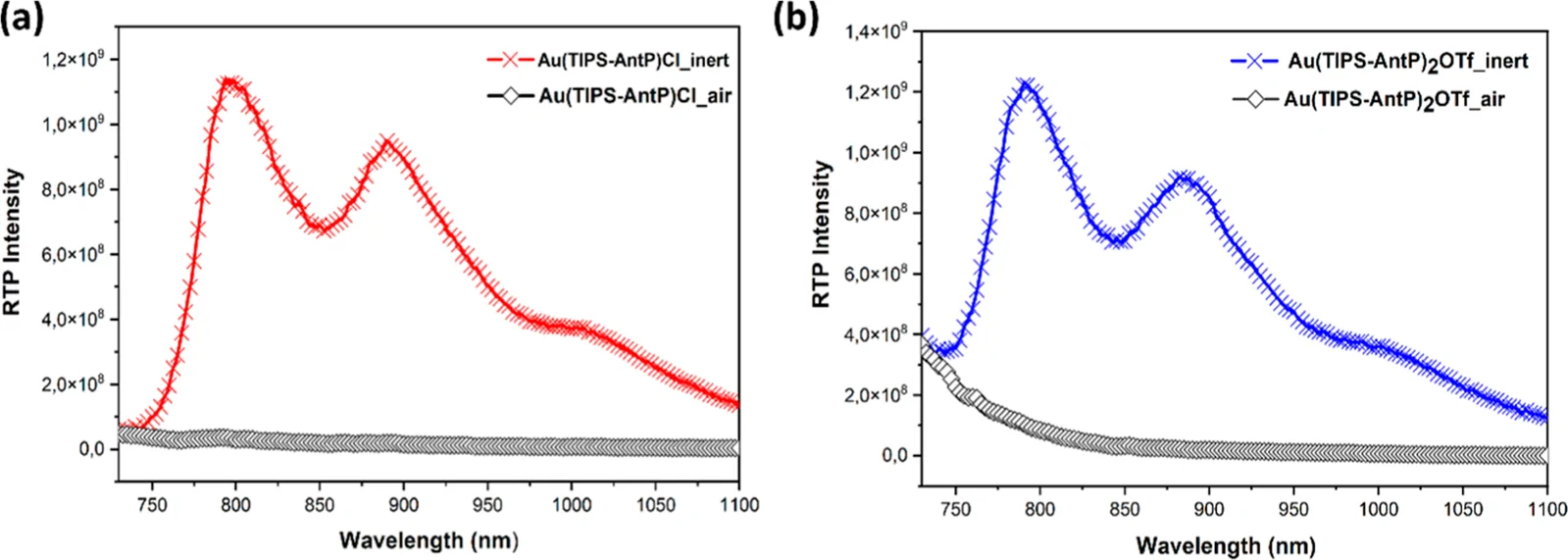
Room-temperature phosphorescence emission spectra (λ_exc_ = 430 nm) of (a) [Au­(TIPS-AntP)­Cl] and (b) [Au­(TIPS-AntP)_2_OTf] recorded in toluene under inert and air conditions at room temperature.

To determine whether RTP originates from metal coordination or is inherent to the ligand framework, we also recorded the RTP spectra of TIPS-AntP and TIPS-AntPO (Figure S32). TIPS-AntP exhibits phosphorescence at 783 nm (1.58 eV) and TIPS-AntPO at 790 nm (1.57 eV), indicating that sufficient ISC occurs within the ligands. Comparison across the series reveals that the emission onset is red-shifted in the Au­(I) complexes, indicating stabilization of the triplet state upon metal coordination. Absorption normalized RTP intensities of both the Au­(I) complexes are found to be approximately twice as high as that of the free ligand. Overall, these observations suggest that in both complexes, the phosphorescence originates primarily from ligand-centered triplet states, with the enhanced emission intensity arising from SOC induced by the Au­(I) center (Figure S33).

### Photoinduced Electron Transfer Reactivity

Since both Au­(I) complexes efficiently generate long-lived triplet states, we investigated their reactivity in photoinduced electron-transfer reactions as a model for potential photocatalytic activity. Tetrachloro-*p*-benzoquinone (chloranil, Chl) was selected as the electron acceptor because it is a comparatively strong electron acceptor with a reduction potential of −0.36 V vs Fc/Fc^+^ in benzonitrile (PhCN), and it exhibits a distinct spectroscopic signature for the one-electron reduced radical anion (Chl^•–^) at ∼440 nm.^63^ Electron transfer reactions were studied using nsTA in PhCN, which facilitates stabilization of the reaction products.

With selective excitation of [Au­(TIPS-AntP)­Cl] at 470 nm, the triplet lifetime decreased from 28 μs to about 13 μs as the concentration of Chl was increased from 10 to 100 μM (Figure 6a). In the corresponding transient absorption spectra (Figure 6b), a decrease in the PIA at 550 nm is observed, accompanied by the emergence of new PIA near 440 nm characteristic of Chl^•–^. In this region, spectral overlaps occur between the Chl^•–^ associated PIA and the GSB of the Au­(I) complex. Furthermore, the Chl^•–^ signal is longer lived than the unquenched triplet in PhCN, which is often observed for photogenerated radical species.^64^ Monitoring the triplet decay as a function of Chl concentration yielded a Stern–Volmer plot (Figure S35) with a bimolecular quenching constant of 4.3 × 10^8^ M^–1^ s^–1^.

**Figure 6:**
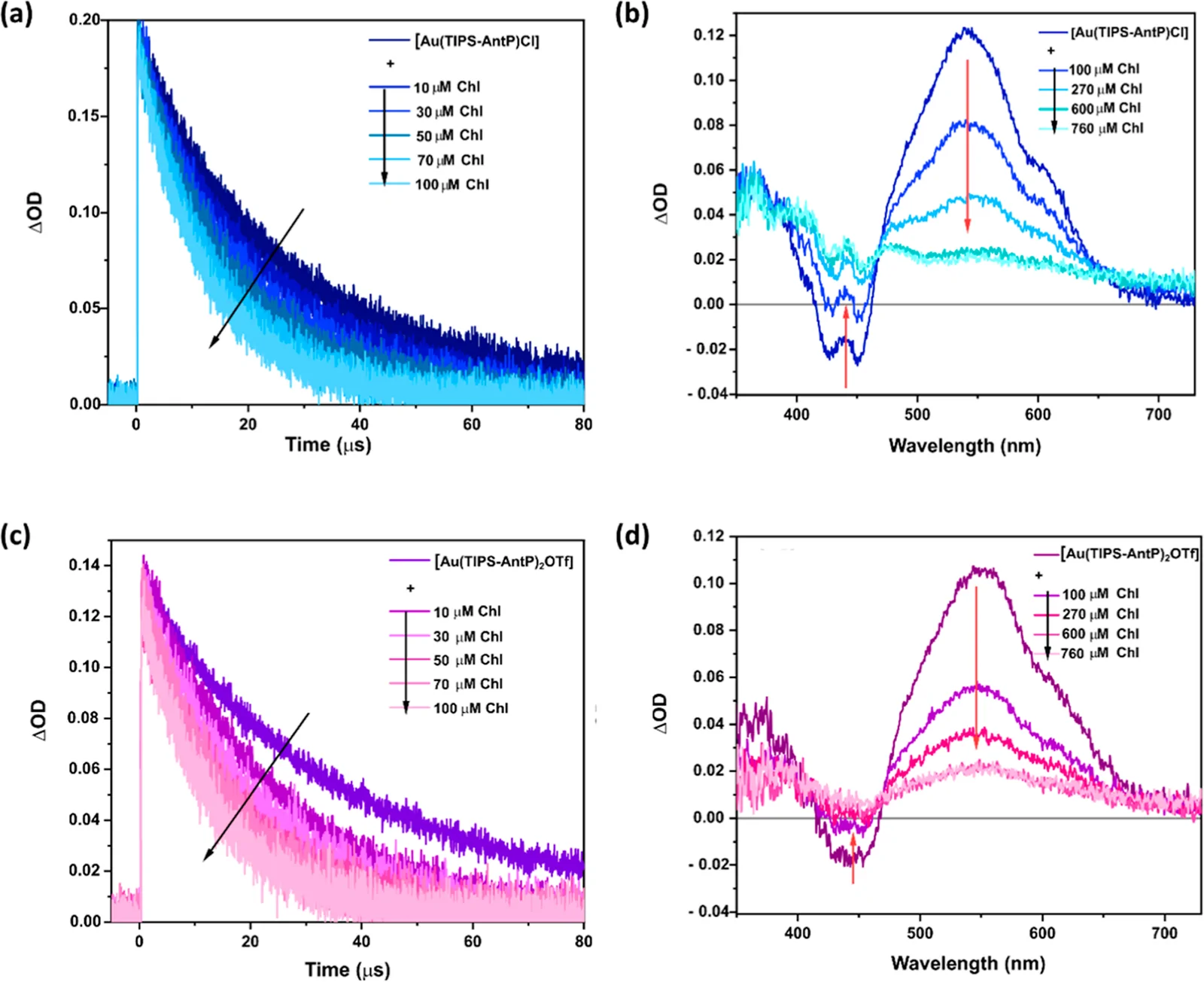
Kinetic traces at 548 nm for (a) monomeric complex [Au­(TIPS-AntP)­Cl] and (c) dimeric complex [Au­(TIPS-AntP)_2_OTf], excited at 470 nm with increasing concentrations of chloranil (Chl). nsTA spectra at 10 μs of (b) monomeric complex [Au­(TIPS-AntP)Cl and (d) dimeric complex [Au­(TIPS-AntP)_2_OTf] excited at 470 nm with increasing concentrations of chloranil (Chl).

For the dimeric complex [Au­(TIPS-AntP)_2_OTf], the triplet lifetime decreased from 42 to 13 μs under similar conditions (Figure 6c), giving a bimolecular quenching constant of 6.9 × 10^8^ M^–1^ s^–1^. Cyclic voltammetry revealed the first oxidation wave at 0.93 V vs Fc/Fc^+^ for [Au­(TIPS-AntP)­Cl] and at 0.74 V vs Fc/Fc^+^ for the dimeric [Au­(TIPS-AntP)_2_OTf] complex in THF (Figure S36). The driving force for electron transfer to Chl was estimated using the relation: Δ*G*_ET_ = *E*_(D/D+)_ – *E*_(A/A−)_ – Δ*E*_0–0_, where Δ*G*_ET_ is the Gibbs free energy change for electron transfer, *E*_(D/D+)_ is the ground-state oxidation potential of the donor, *E*_(A/A−)_ is the ground-state reduction potential of the acceptor, and Δ*E*_0–0_ is the triplet energy. Using this approach, Δ*G*_ET_ values of −0.26 V for [Au­(TIPS-AntP)­Cl] and −0.47 V for [Au­(TIPS-AntP)_2_OTf] were obtained, indicating that photoinduced electron transfer to Chl is strongly exergonic in both cases.

These results support that both the Au­(I) complexes are robust photoreductants. Based on the triplet energy given in Table 1, the excited-state oxidation potential of [Au­(TIPS-AntP)­Cl] and [Au­(TIPS-AntP)_2_OTf] is also estimated as −0.62 V and −0.83 V, confirming the dimeric Au­(I) complex is roughly 0.2 V more reducing in its excited triplet state compared to the former, which also aligns with its slightly higher electron transfer rate constant. Even though, despite this stronger driving force, the spectral signal corresponding to Chl^•–^ in the dimer (Figure 6d) is significantly weaker than the monomer. A plausible explanation is that the positive charge on the dimeric Au­(I) core hinders charge separation and promotes more rapid charge recombination. The rise of the Chl^•–^ signal for both systems is provided in Figure S35.

### Singlet Oxygen Production

Triplet states with sufficiently long triplet lifetimes can sensitize ground-state molecular oxygen O_2_(^3^Σ*g* ^–^) to generate singlet oxygen O_2_(^1^Δ*g*). Direct detection of the characteristic near-infrared phosphorescence at ∼1270 nm provides clear evidence for singlet oxygen formation, corresponding to the ^1^Δ*g* → ^3^Σg^–^ relaxation pathway. In the present systems, the triplet energies 1.55 eV for [Au­(TIPS-AntP)­Cl] and 1.57 eV for [Au­(TIPS-AntP)_2_OTf] are well above the excitation energy of O_2_(^1^Δ*g*) (0.98 eV), making triplet–triplet energy transfer to oxygen (O_2_(^3^Σg^–^) favorable.^65^ Both the complexes were excited at λ_exc_ = 422 nm in air-equilibrated dichloromethane solutions. As anticipated, strong O_2_(^1^Δ*g*) phosphorescence at ∼1274 nm was clearly observed for both compounds (Figure 7). The singlet oxygen quantum yields (Φ_Δ_) were determined relative to zinc tetraphenylporphyrin (ZnTPP, Φ_Δ_ = 68%).^66^ For [Au­(TIPS-AntP)­Cl], Φ_Δ_ was ∼71%, whereas the dimeric [Au­(TIPS-AntP)_2_OTf] complex showed a significantly higher Φ_Δ_ of ∼97% in air-saturated DCM.

**Figure 7:**
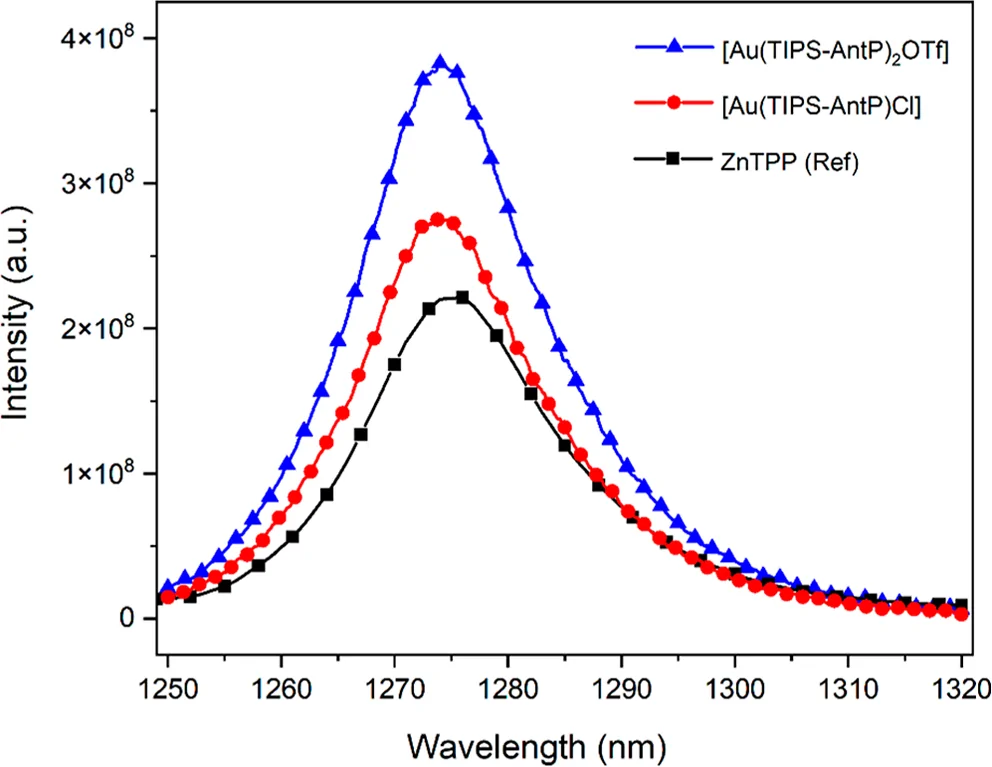
Singlet oxygen (O_2_(^1^Δ*g*)) emission of Au­(I) complexes in air-equilibrated DCM solutions at λ_exc_ = 422 nm.

## Conclusion

In this work, we developed a new TIPS-anthracene scaffold functionalized with diphenylphosphine groups to enable metal coordination and successfully synthesized two TIPS-anthracene-based Au (I) complexes. Comprehensive photophysical and transient spectroscopic studies reveal that the free ligands and Au­(I) complexes effectively generate triplet states with a lifetime of τ_t__riplet_ ≈ 40–90 μs. In particular, the dimeric Au­(I) complex exhibits a more stabilized triplet exciton with a markedly slower decay, offering new design strategies for acene-based triplet mediating materials. The triplet (T_1_) energies and long lifetimes of both the Au­(I) complexes were sufficient to enable room-temperature phosphorescence and highly efficient singlet-oxygen generation. Although ISC is fast in both ligand and complex, phosphorescence is slightly enhanced by Au­(I) coordination. Additionally, both complexes display strong photoreductant capabilities, engaging in photoinduced electron transfer reactions with close to diffusion-limited bimolecular rate constants. These findings demonstrate that Au­(I) coordination preserves the intrinsic triplet characteristics of the TIPS-anthracene core while enabling tunable photochemical functionality through metal–ligand interactions. Overall, this work establishes TIPS-anthracene frameworks as a versatile platform for engineering triplet-active molecular systems relevant to photoredox catalysis, oxygen sensitization, and solar energy conversion.

## Experimental Section

### General Procedures

All the reactions were performed under a purified argon atmosphere using the standard high-vacuum Schlenk-line technique. All the solvents used were purchased from VWR and dried before using. ^n^BuLi (1.6 M in hexanes), NaBH_4_, and AgOTf were purchased from Sigma-Aldrich and used as received. Triisopropylsilyl acetylene, SnCl_2_·2H_2_O, and PPh_2_Cl were obtained from Fluorochem and used as received. Au­(THT)Cl was prepared according to literature methods.^67^ The synthetic procedures of compounds **B**–**G** are given in Supporting Information.

### Synthesis of Au­(I) Complexes

#### Compound H, [Au­(TIPS-AntP)­Cl)]

Under an inert atmosphere, an oven-dried Schlenk flask was charged with compound F (TIPS-AntP, 0.16 g, 0.2 mmol) and Au­(THT)Cl (0.065 g, 0.2 mmol) in anhydrous DCM (10 mL). The reaction mixture was stirred at room temperature for 2 h. After completion, the solvent was removed under reduced pressure, and the crude product was purified by silica gel column chromatography using a gradient of pentane/ethyl acetate (from 100:0 to 5:1) to yield compound H as a yellow solid (0.11 g, 0.1 mmol, 52%). A block-shaped single crystal of the compound was grown from the DCM/diethyl ether reaction mixture. ^1^H NMR (400 MHz, CDCl_3_): δ [ppm]: 9.16 (s, 1H), 9.07 (s, 1H), 8.05–7.95 (dd, 2H), 7.71–7.76 (dd, 4H), 7.63–7.56 (t, 2H), 7.51–7.41 (m, 6H) 1.23–1.22 (m, 21H), 1.06–1.04 (m, 21H). ^13^C NMR (400 MHz, CDCl_3_): δ [ppm]: 133.99, 133.84, 132.73, 131.97, 131.67, 131.53, 130.52, 130.31, 129.69, 129.38, 129.25, 128.85, 128.57, 128.03, 127.81, 127.32, 126.57, 119.00, 106.92, 103.41, 101.88, 19.02, 11.42. ^31^P NMR (400 MHz, CDCl_3_): δ [ppm]: 35.91. ESI-MS­(*m*/*z*) calculated for C_48_H_58_AuBrClPSi_2_: 1033.2421; found, [M + H]^+^ 1034.2331, [M – Cl]^+^ 999.2653.

#### Compound I, [Au­(TIPS-AntP)_2_)­OTf]

TIPS-AntP (0.08 g, 0.1 mmol) was dissolved in 10 mL of anhydrous DCM in an oven-dried, argon-purged Schlenk flask, followed by the addition of Au­(THT)­Cl (0.017 g, 0.05 mmol). After 15 min of stirring, AgOTf (0.013 g, 0.05 mmol) was added to the solution and further stirred for 3 h for a complete reaction. AgCl formed during the reaction was filtered under an inert atmosphere, and the resulting solution was dried and washed with pentane to get a yellow crystalline powder (0.05 g, 0.026 mmol, 52%). ^1^H NMR (400 MHz, CDCl_3_): δ [ppm]: 9.15 (s, 2H), 9.08 (s, 2H), 8.06–7.96 (dd, 4H), 7.69–7.64 (dd, 4H), 7.56–7.49 (m, 20H) 1.24–1.22 (m, 42H), 0.97 (m, 42H). ^13^C NMR (400 MHz, CDCl_3_): δ [ppm]: 134.43, 133.66, 133.24, 132.82, 132.05, 131.86, 131.29, 131.03, 130.18, 130.05, 129.49, 129.12, 128.90, 128.68, 128.27, 128.04, 127.31, 127.04, 126.62, 125.75, 119.94, 106.31, 103.47, 19.27, 19.15, 12.03, 11.84. ^31^P NMR (400 MHz, CDCl_3_): δ [ppm]: 42.64. ESI-MS­(*m*/*z*) calculated for C_96_H_116_AuBr_2_P_2_Si_4_: 1800.5734; found, [M]^+^ 1800.5838.

## Supplementary Material



## Data Availability

Data underlying the the figures in this publication is available at Zenodo data repository at https://doi.org/10.5281/zenodo.20814580.

## Notes

Materials and methods, synthesis and characterization, X-ray crystallographic data, photophysical measurements, TD-DFT calculations, transient spectroscopic data, electrochemical data, and electron transfer data. An early version of the manuscript has been deposited on ChemRxiv.^68^ (PDF).
