# Supplementary material for: Harnessing Triplets of TIPS-Anthracene-Based Au(I) Complexes for Photoinduced Electron Transfer and Room-Temperature Phosphorescence
Source: Inorg Chem. 2026 Aug 4;65(32):18532–42. doi: 10.1021/acs.inorgchem.6c01047 (PMC13488395; doi:10.1021/acs.inorgchem.6c01047)
Supplement: Supplementary file 1 [file ic6c01047_si_001.pdf]

Supporting Information

Harnessing Triplets of TIPS-Anthracene Based Au(I) Complexes for Photoinduced Electron Transfer and Room-temperature Phosphorescence

Avik Bhanja[a,b], Sree Chithra,[a] Rafael Araujo[c], Ebin Sebastian[d], Tomas Edvinsson[c], Victor Gray*[a]

[a]Department of Chemistry–Ångström Laboratory, Uppsala University, 75237, Sweden
[b]Department of Chemistry and Materials Science, Aalto University, 02150, Finland
[c] Department of Materials Science and Engineering, Uppsala University, 75237, Sweden
[d] Department of Physics, Cavendish Laboratory, University of Cambridge, CB3 0HE, UK

Email: victor.gray@kemi.uu.se

Table of Contents

Content **Page No.**

Content	Page No.
1. Experimental procedures	S3
1.1. General details	S3
1.2. Spectroscopic measurements	S3
1.2.1. Nanosecond Transient Absorption (nSTA) measurement	S4
1.2.2. Femtosecond Transient Absorption (fSTA) measurement	S4-S5
1.3. Electrochemistry	S5
1.4. X-ray crystallographic details	S5-S6
1.5. Computational details	S6
2. Synthesis and Characterization	S6-S10
3. Spectral data	S11-S21
4. Refinement details for the X-ray structures	S21-S22
5. Packing diagrams	S22
6. Photophysical Measurements	S23-S24
6.1 Quantum yield determination	S24
7. TD-DFT Calculations	S24-S26
8. Transient Absorption Spectroscopy	S26-S28
9. Room Temperature Phosphorescence	S28-S29
10. Photoinduced Electron Transfer	S30
11. Electrochemistry	SS31
12. Singlet oxygen quantum yield determination	S32
13. Cartesian coordinates	S32-S36
14. References	S36-S37

1. Experimental Procedures

1.1. General details

All the glassware was oven-dried and cooled under vacuum and purged with Argon before use. Solvents were freshly collected from *Pure Solve MD7* solvent purification system (SPS) under Argon. Argon was supplied by Air Liquide Europe. All the NMR spectra were recorded on a Jeol 400 MHz spectrometer (^{1}H, 400 MHz; ^{13}C, 101 MHz; ^{31}P, 162 MHz) at 298 K. Chemical shift values are quoted in δ (ppm) and coupling constants in *J* (Hz). ^{1}H chemical shift values were reported relative to tetramethylsilane (TMS) and were referenced to the residual proton resonances of the corresponding deuterated solvent signal. ^{13}C{^{1}H} NMR spectra are reported relative to TMS using the natural-abundance carbon resonances of the deuterated solvents. ^{31}P NMR spectra are referenced externally to H_3PO_4 (85%, aq.). The following abbreviations (or combinations thereof) were used to describe multiplicities: s, singlet; d, doublet; t, triplet; m, multiplet. For column chromatography, silica gel (40-63 μm) from VWR was used. A gradient elution using pentane and EtOAc was performed, based on Merck aluminium TLC sheets (silica gel 60F254). High-resolution mass spectrometry (HRMS) experiments were performed in positive ionization mode using a matrix-assisted laser desorption/ionization Fourier-transform ion cyclotron resonance (MALDI-FTICR) (7T solariX XR-2ω, Bruker Daltonics) mass spectrometer equipped with a Smartbeam II 2 kHz laser. For HRMS experiments, the compounds were dissolved in dry THF and mixed 1:1 with 2,5-dihydroxy benzoic acid (DHB) matrix solution (35mg/ml in dry THF) and 1 μl of mixed solution from each compound was spotted on MTP 384 ground steel target plates (Bruker Daltonics) and left for drying at room temperature. Either M^{+} or $[M+H]^{+}$ ions were detected for each compound. Molecular ion m/z values of each compound were calculated using Isotope pattern software (Bruker Daltonics). Molecular isotopic distributions of each molecule were also checked for validating the identity of the molecule.

1.2. Spectroscopic measurements

All the samples were prepared inside an argon glovebox with oxygen levels <1 ppm. The solvents were obtained from a Solvent Purification System (SPS) from and then freeze pump thawed before bringing them inside the glovebox. The steady state UV-visible absorption was performed using Agilent Cary 60 and the steady-state fluorescence spectra were obtained using Fluorolog 3-222 emission spectrophotometer (Horiba Jobin-Yvon). Spectra were acquired with FluorEssence software using a right-angle detector geometry (90° angle) along with corrections for variations in the lamp output and detector response. Fluorescence quantum yields were

measured using 9,10-Bis(phenylethynyl)anthracene in degassed toluene ($\phi_F = 0.85$) as the reference.[1] Singlet oxygen emission was recorded on a FLS 1000 fluorimeter with a nitrogen cooled NIR PMT detector.

1.2.1 Nanosecond transient absorption (nsTA) measurements

The nsTA measurements were carried out using a Nd:YAG laser (Ekspla, NT342B laser) as the source of excitation of the samples with an OPO set at 430 nm having energies of ~2 mJ/pulse. Sensitized spectra were excited at 545 nm. The spectrometer (LP920, Edinburgh Instruments) comprises a pulsed 450 W ozone-free Xe arc lamp, a symmetrical Czerny-Turner monochromator (TMS300) with 5 nm bandwidth and detectors for both single kinetic traces (LP900 photomultiplier, with Tektronix TDS3012C oscilloscope) and entire spectra (Andor SH720 ICCD camera). The kinetic traces were fit using a home-written Matlab script using least-square minimization.

1.2.2 Femtosecond transient absorption (fsTA) measurements

The fsTA measurements were carried out using a Ti:sapphire-based amplifier along with an integrated oscillator and pump lasers (Coherent Libra). A beam splitter splits the laser fundamental (800 nm, 3 kHz, fwhm 40fs) into a pump and probe which were then directed towards the UV–vis–NIR TA spectrometer (TAS, Newport Corp.). Optical parametric amplifiers (TOPAS NirUVis, Light Conversion) were used to generate the excitation wavelength (pump-450 nm). The pump was passed through a waveplate that was set at magic angle and attenuated using a neutral density filter before reaching the sample. A calcium fluoride crystal was used to generate the probe supercontinuum (UV–vis) and an optical delay ($t_{\text{window}} \leq 8$ ns) was used to vary the path, thereby recording the transient spectra at varying pump–probe delay times on a silicon diode array (Newport custom-made). The samples were kept in 1 mm quartz cuvettes and had an absorbance of 0.2-0.3 at 450 nm. A pump power of 0.2 mW was used for measurements and focused on the sample in an approximately 0.05 mm^2 spot.

Due to laser failure some samples were measured on a different setup. This setup comprised a Yb-based laser and a HARPIA fsTA setupt (Light Conversion). The fundamental output of a PHAROS femtosecond laser (1030 nm, 10 kHz; Light Conversion) was split to provide pump and probe paths. Tunable pump pulses were generated using an ORPHEUS optical parametric amplifier (Light Conversion) operated in a collinear geometry. For the probe, UV–blue white-light continuum (≤ ~520 nm) was produced by first frequency-doubling the 1030 nm beam to 515 nm in a β-BaB_2O_4 (BBO) crystal and then focusing the 515 nm

pulses into a sapphire plate. For longer-wavelength coverage (> ~520 nm; visible–NIR), the 1030 nm fundamental was focused directly into sapphire to generate the supercontinuum. Pump and probe were spatially and temporally overlapped at the sample, with the delay controlled by the HARPIA delay line. To suppress rotational-diffusion artifacts, the pump polarization was set to the magic angle (54.7°) relative to the probe. Transient signals were recorded as $\Delta A(\lambda, t)$. Visible and UV regions were recorded as separate experiments. The samples were irradiated at 440 nm with a pump power of 61 μW. All TA datasets were background and chip corrected using a home-written Matlab script. Single-value decomposition and target analysis with a two-state model was also performed using the same script.

1.3. Electrochemistry

Cyclic voltammograms (CV) were obtained at room temperature using an AUTOLAB PGSTAT 302 potentiostat. The experiments were performed in an Argon atmosphere glovebox with levels of O_2 (<1 ppm) and H_2O (<1 ppm). The experiment was performed in a cylindrical 2.5 cm x 5 cm glass cell. A 3 mm diameter glassy carbon disc electrode was used as a working electrode which was polished carefully with 0.05-micron Al particle paste on a polishing pad, a Pt wire was used as a counter electrode, and as a reference electrode a pseudo reference Ag/Ag^+ silver wire was used, covered with two vycor frits. As a supporting electrolyte 0.1 M tetrabutylammonium hexafluorophosphate ($^nBu_4PF_6$) was used (≥99.0 %, Sigma-Aldrich). Before use, $^nBu_4PF_6$ was recrystallized from ethanol and the dried in a vacuum oven at 85 ^oC. The typical experiments were performed with 5 ml solvent with the 100 mM supporting electrolyte and 1 mM of the compounds was added to the cell with a scan rate of 100 mV/s. To determine the potential, ferrocene was added at the end of each experiment as an internal reference.

1.4. X-ray crystallographic details

Single crystals of TIPS-AntPO and [Au(TIPS-AntP)Cl] were obtained by layer diffusion technique using DCM layered with diethyl ether. A suitable crystal was selected and mounted using Fomblin oil on a fiber-loop on a XtaLAB Synergy, Single source (CuKα) diffractometer equipped with a HyPix detector. The crystal was kept at 150.00(10) K during data collection. The structure was solved using Olex2,[2] with the SHELXT structure solution program using intrinsic phasing and refined with the SHELXL[3] refinement package using Least Squares minimization. All the non-hydrogen atoms were refined using an anisotropic model and all the hydrogen atoms were constrained in geometrical positions to their parent atom. Crystallographic data are presented in Table S1. Deposition Numbers 2491607 (TIPS-AntPO)

and 2491609 ([Au(TIPS-AntP)Cl]) contain(s) the supplementary crystallographic data for this paper.

1.5. Computational details

All electronic structure calculations were performed using the Jaguar quantum chemistry package.[4] The ground-state geometries of the investigated molecules were optimized within the framework of density functional theory (DFT), employing the B3LYP hybrid functional[5-7] with Grimme's D3 dispersion corrections.[8,9] The LACVP**++ basis set was used throughout, which combines the Los Alamos core–valence pseudopotential with double-ζ polarization and diffuse functions. For the dimer, five different initial structural configurations were tested, and the lowest-energy geometry was selected for further analysis. Vibrational frequency calculations were carried out at the same level of theory to verify the nature of the stationary point for the lowest energy structure. Only small imaginary modes (< 20 cm^{-1}) were observed, which lie within the typical range of numerical noise and are attributed to low-energy torsional or collective motions rather than genuine instabilities. Excited-state properties were investigated within the time-dependent DFT (TD-DFT) formalism under the Franck–Condon approximation for vertical transitions.[10,11] Both singlet and triplet manifolds were explored, and the lowest-energy excitations were analyzed in terms of dominant orbital contributions, excitation energies, oscillator strengths, and transition dipole moments.

2. Synthesis and Characterization

Scheme S1 Synthetic Diagram of TIPS-anthracene-based Phosphine Ligand

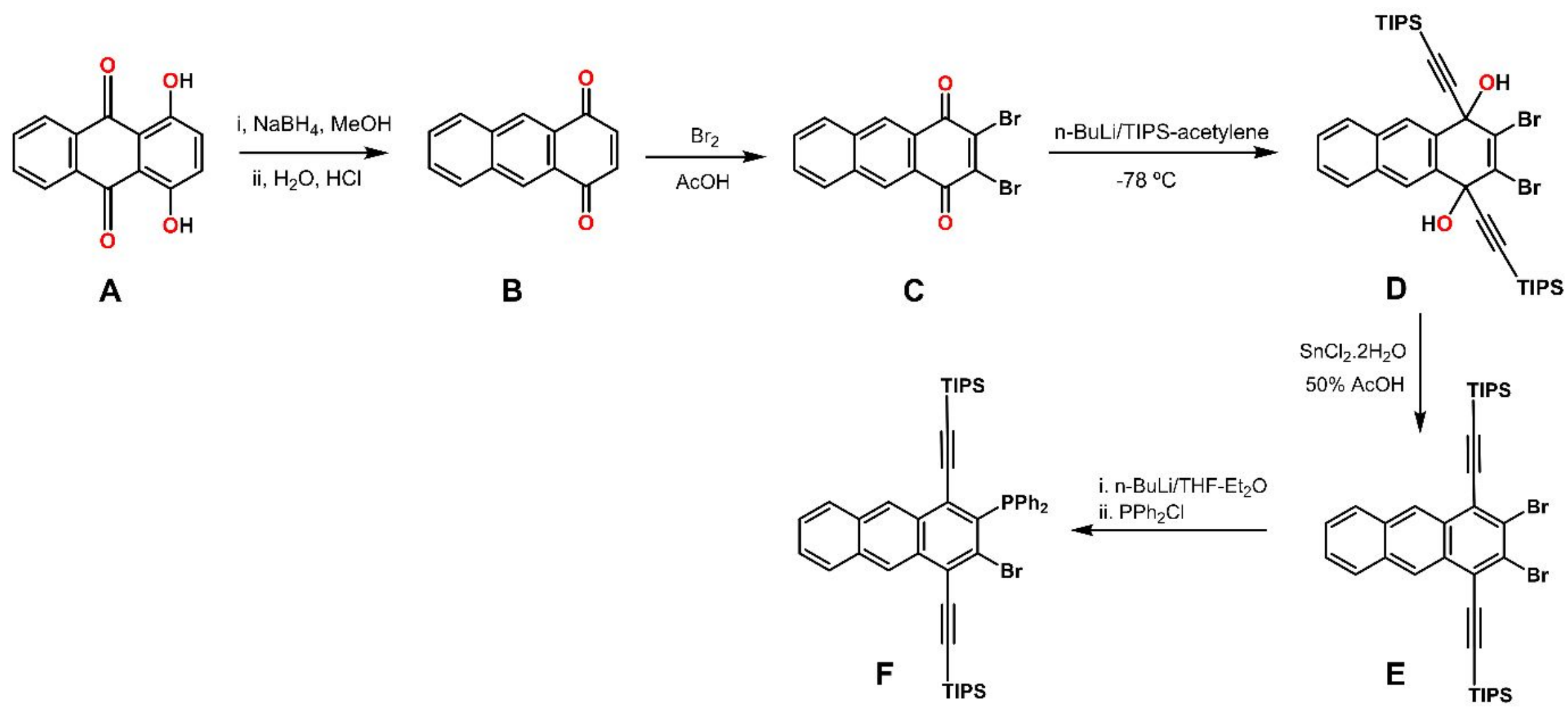

A triisopropylsilyl (TIPS) ethynyl-substituted anthracene-based phosphine ligand was prepared following a multi-step synthetic protocol outlined in Scheme S1. Compound **A** to **C** was

synthesized by following a modified synthetic protocol.[12] First 1,4-dihydroxyanthraquinone (**A**) was reduced to 1,4-anthraquinone (**B**), which was then brominated to form 2,3-dibromoanthraquinone (**C**). Subsequent butylation and reduction with stannous chloride led to the formation of dibromo-TIPS-anthracene (**E**). A controlled, single lithium–halogen exchange was then performed to selectively remove one bromine atom and the resulting intermediate was reacted with PPh_2Cl to yield the final phosphine-functionalized product (**F**).

Synthesis of compound B (1,4-anthraquinone):

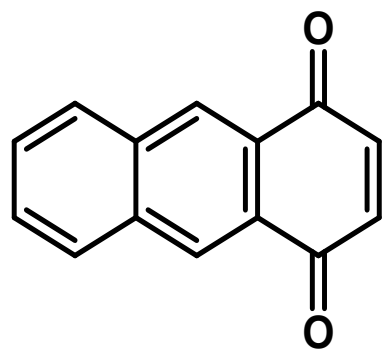

Compound A, 1,4-dihydroxyanthracene-9,10-dione (5.00 g, 20.8 mmol) was dissolved in methanol (100 mL) and the solution was cooled to 0 °C. Sodium borohydride (5.00 g, 132.1 mmol) was added gradually with stirring. The reaction mixture was maintained at 0 °C and stirred for 90 minutes. After completion, aqueous hydrochloric acid (6 M, 90 mL) was added slowly, leading to the formation of a precipitate. The solid was collected by filtration, washed with water, and dried to afford 1,4-anthraquinone as a brownish orange solid (3.9 g, 18.73 mmol, 90%). ^{1}H NMR (400 MHz, DMSO-d_6) δ [ppm]: 8.63 (s, 2H), 8.30 – 8.27 (dd, 2H), 7.79 – 7.76 (dd, 2H), 7.16 (s, 2H). ^{13}C NMR (400 MHz, DMSO-d_6) δ [ppm]: 184.25, 139.95, 134.15, 130.04, 129.59, 128.06, 128.01.

Synthesis of compound C (2,3-dibromoanthraquinone):

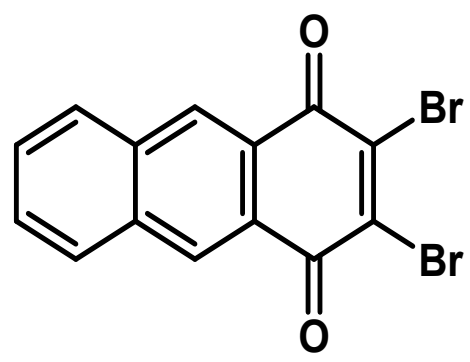

1,4-anthraquinone (6.8 g, 32.5 mmol) was dissolved in glacial acetic acid (200 mL) and kept the flask under a cold-water bath. Liquid bromine (3.5 mL, 65 mmol, 2 equivalents) was added slowly with dropwise manner under stirring condition at room temperature. The resulting mixture was stirred for 30 mins at ambient temperature and then heated to 110 °C for 6 hrs. Then the reaction mixture was cooled and poured into ice-water (500 mL). The resulting brown precipitate was collected by filtration, washed carefully with water, and dried under reduced pressure to yield the desired brown crystalline solid (11.1 g, 30.3 mmol, 93%). ^{1}H NMR (400

MHz, DMSO-d_6) δ [ppm]: 8.73 (s, 2H), 8.32 – 8.30 (dd, 2H), 7.82 – 7.79 (dd, 2H). ^{13}C NMR (400 MHz, DMSO-d_6) δ [ppm]: 176.05, 134.72, 130.68, 130.48, 129.09, 128.49, 127.39.

Synthesis of compound D (2,3-dibromo-1,4-bis(triisopropylsilylethynyl)-1,4-dihydroanthracene-1,4-diol):

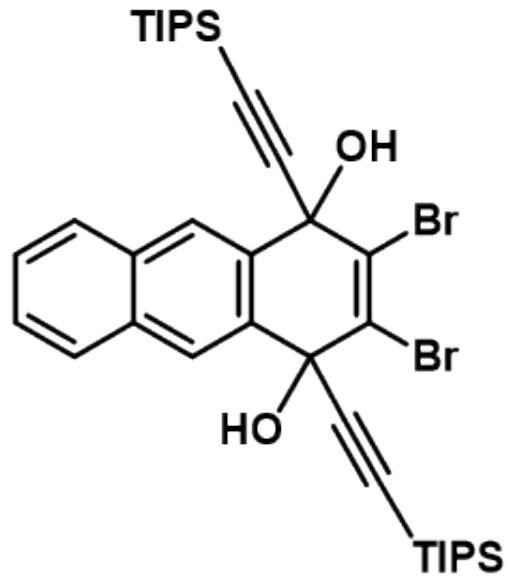

A flame-dried, argon-purged 250 mL Schlenk flask was charged with dry pentane (75 mL), followed by the addition of triisopropylsilyl acetylene (6.8 mL, 30 mmol). The reaction mixture was cooled to −78 °C, and a solution of n-BuLi (1.6 M in hexane, 16.7 mL, 26.7 mmol) was added dropwise under stirring. The mixture was maintained at −78 °C for 30 min, then allowed to warm to room temperature and stirred for an additional 45 min to ensure complete metal–halogen exchange. In a separate flame-dried Schlenk flask, compound C (2,3-dibromoanthraquinone) (3.66 g, 10 mmol) was dried under vacuum, purged with argon, and dissolved in anhydrous THF (75 mL). This solution was transferred via cannula to the previously prepared Li-exchanged TIPS-acetylene solution under an argon atmosphere. The resulting clear brown reaction mixture was stirred at room temperature for 16 h. After that the reaction was quenched by addition of 1 mL water and the solvent was evaporated to dryness. Residual TIPS-acetylene in the crude mixture was removed by repeated washings with pentane. The crude product was further purified by column chromatography with 10% ethyl acetate/pentane to afford light brown solids (7.1 g, 9.7 mmol, 97%). ^{1}H NMR (400 MHz, DMSO-d_6) δ [ppm]: 8.31 (s, 2H), 7.95–7.92 (dd, 2H), 7.55–7.52 (dd, 2H), 7.03 (s, 2H), 0.97–0.95 (m, 42H).

Synthesis of compound E (2,3-dibromo-1,4-bis(triisopropylsilylethynyl)-anthracene):

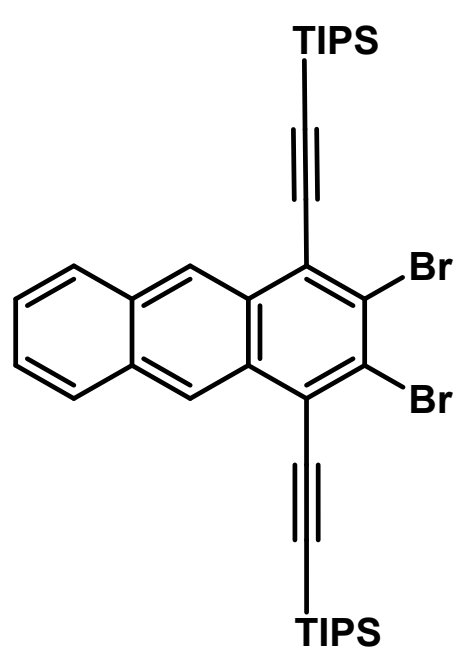

To a stirred solution of compound D (7.1 g, 9.7 mmol) in THF (100 mL) was added a suspension of stannous chloride dihydrate (17.5 g, 77.6 mmol) in a 1:1 mixture of water and acetic acid (100 mL). The reaction mixture was then heated at 50 °C for 3 h. Upon completion, the solvent was removed under reduced pressure, and the resulting brownish orange residue was subjected to solvent extraction using a 1:1 mixture of water and chloroform. The organic layer was separated and washed with H_2O (5 × 150 mL), dried with $MgSO_4$, and concentrated in vacuo. The crude product was further purified by column chromatography with pentane as the eluent to afford a crystalline yellow solid. (4.1 g, 5.9 mmol, 61%). ^{1}H NMR (400 MHz, $CDCl_3$) δ [ppm]: 8.98 (s, 2H), 8.00–7.98 (dd, 2H), 7.56–7.54 (dd, 2H), 1.27–1.26 (m, 42H). ^{13}C NMR (400 MHz, $CDCl_3$) δ [ppm]: 133.18, 131.86, 128.43, 127.88, 126.88, 107.68, 89.41, 18.40, 10.98.

Synthesis of compound F [3-bromo-2-diphenylphosphino-1,4-bis(triisopropylsilylethynyl) anthracene; TIPS-AntP]:

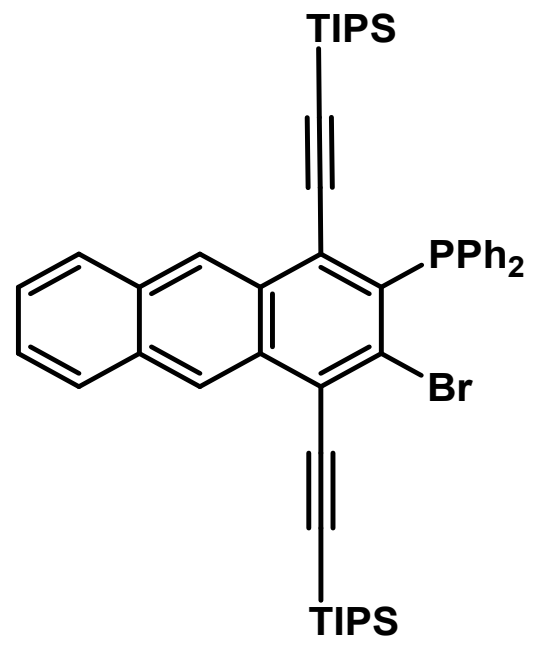

Incorporation of diphenyl-phosphine into the ligand framework was performed following a modified synthetic protocol.[13] A 100 mL Schlenk flask was charged with 2,3-dibromo-1,4-bis(TIPS-ethynyl)anthracene (1.05 g, 1.5 mmol) dissolved in a 1:1 mixture of anhydrous diethyl ether and THF (25 mL each). The solution was cooled to –110 °C, and a solution of n-BuLi (1.6 M in hexanes, 1.1 mL, 1.75 mmol) was added dropwise under stirring, resulting in lithium–bromine exchange. The reaction mixture was maintained at –110 °C for 1 h, after which chlorodiphenylphosphine (0.35 mL, 1.75 mmol) was added and the solution was stirred for another 2 h in a cold bath at –110 °C. After that the mixture was allowed to warm to room temperature and stirred for 16 h. Completion of the reaction was checked by TLC and the volatiles were removed under reduced pressure. The crude compound was redissolved in 20 mL anhydrous diethyl ether and filtered off the excess LiCl under inert atmosphere. The residue was then purified by column chromatography using 10% DCM/pentane as eluent to afford an orange compound (0.6 g, 0.75 mmol, 50%). ^{1}H NMR (400 MHz, $CDCl_3$) δ [ppm]: 9.18 (s, 1H),

9.03 (s, 1H), 8.01–7.97 (dd, 2H), 7.82–7.75 (dd, 2H), 7.61–7.48 (m, 10H), 1.23–1.22 (m, 21H), 1.10–1.09 (m, 21H). ^{13}C NMR (400 MHz, $CDCl_3$) δ [ppm]: 137.96, 136.68, 135.94, 135.78, 133.26, 132.90, 132.47, 132.28, 130.93, 129.12, 128.75, 128.40, 128.31, 127.59, 126.60, 126.05, 125.38, 109.68, 104.86, 104.27, 103.77, 18.82, 11.68. ^{31}P NMR (400 MHz, $CDCl_3$) δ [ppm]: 9.28.

Synthesis of compound G [3-Bromo-2-diphenylphosphineoxide-1,4-bis (triisopropylsilylethynyl) anthracene; TIPS-AntPO)]:

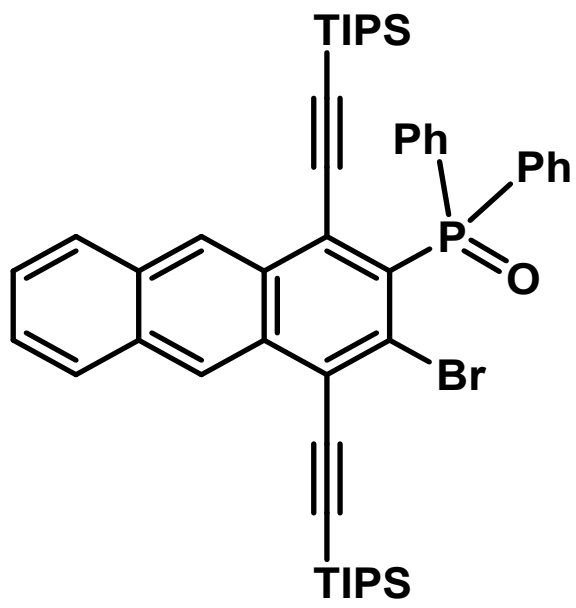

TIPS-ethynyl-substituted anthracene-based phosphine ligand (F) (0.4 g, 0.5 mmol) was dissolved into 30 mL of THF and kept it in an ice-bath. 2 mL of H_2O_2 was added into it and stirred for overnight. The reaction was quenched using saturated sodium sulphite solution and was extracted using DCM (3 × 20 mL). The organic layer was dried (Na_2SO_4) and all volatile components were removed in vacuo to furnish solid yellow powder (0.33 g, 0.4 mmol, 80%), which was purified via recrystallization from DCM/pentane solvent mixture. ^{1}H NMR (400 MHz, $CDCl_3$) δ [ppm]: 9.24 (s, 1H), 9.04 (s, 1H), 8.02–7.95 (dd, 2H), 7.81–7.76 (dd, 2H), 7.60–7.47 (m, 10H), 1.22–1.21 (m, 21H), 1.07–1.05 (m, 21H). ^{13}C NMR (400 MHz, $CDCl_3$) δ [ppm]: 134.85, 133.84, 133.76, 132.50, 132.29, 132.13, 132.02, 131.79, 130.48, 128.85, 128.61, 128.48, 128.18, 127.69, 126.90, 126.22, 125.82, 125.73, 124.92, 114.77, 105.84, 103.80, 102.32, 18.89, 11.52. ^{31}P NMR (400 MHz, $CDCl_3$) δ [ppm]: 31.93.

3. Spectral Data

Compound B

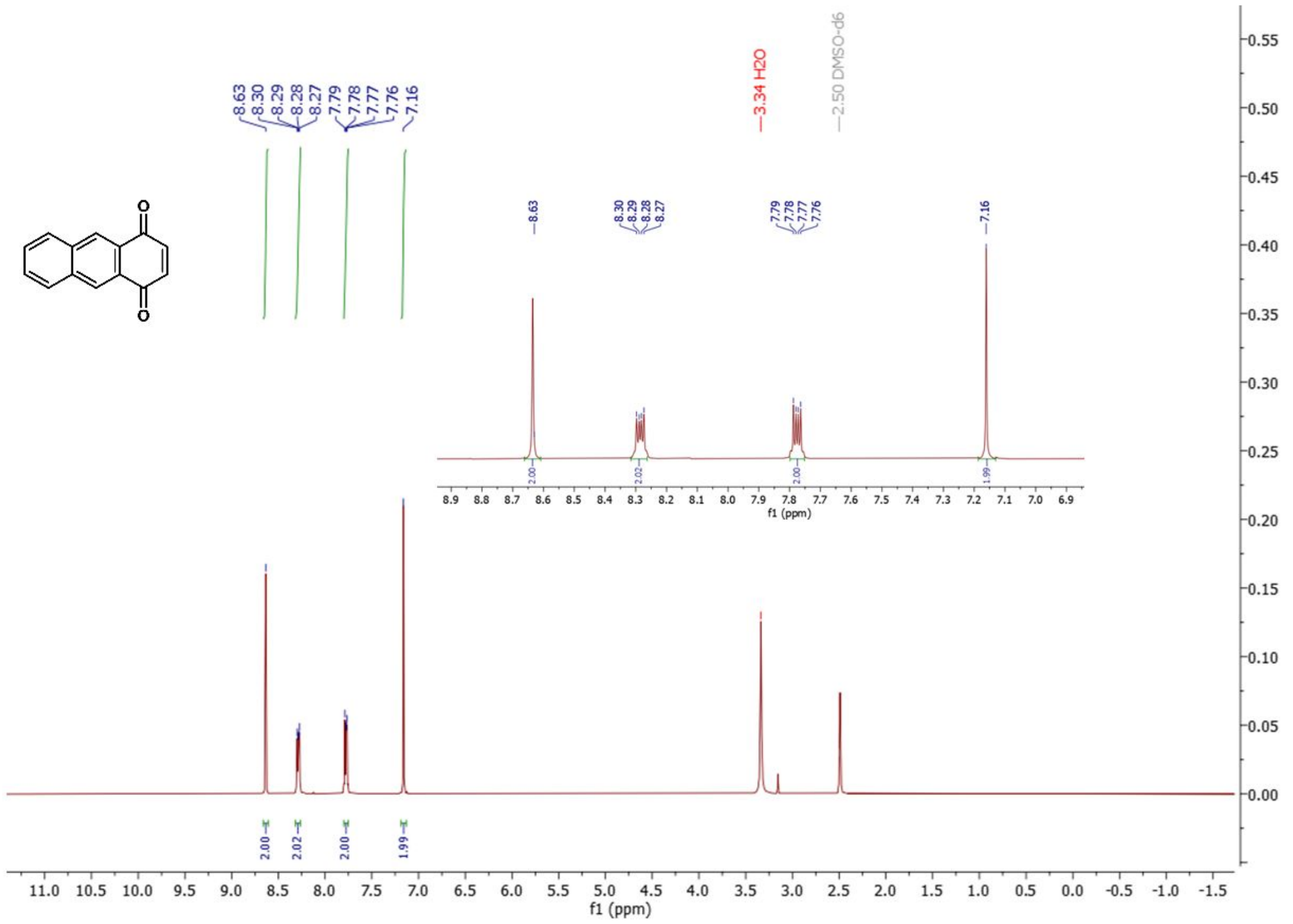

Figure S1. ^{1}H-NMR spectrum of 1,4-anthraquinone (compound B) in DMSO-d_6 at 298 K

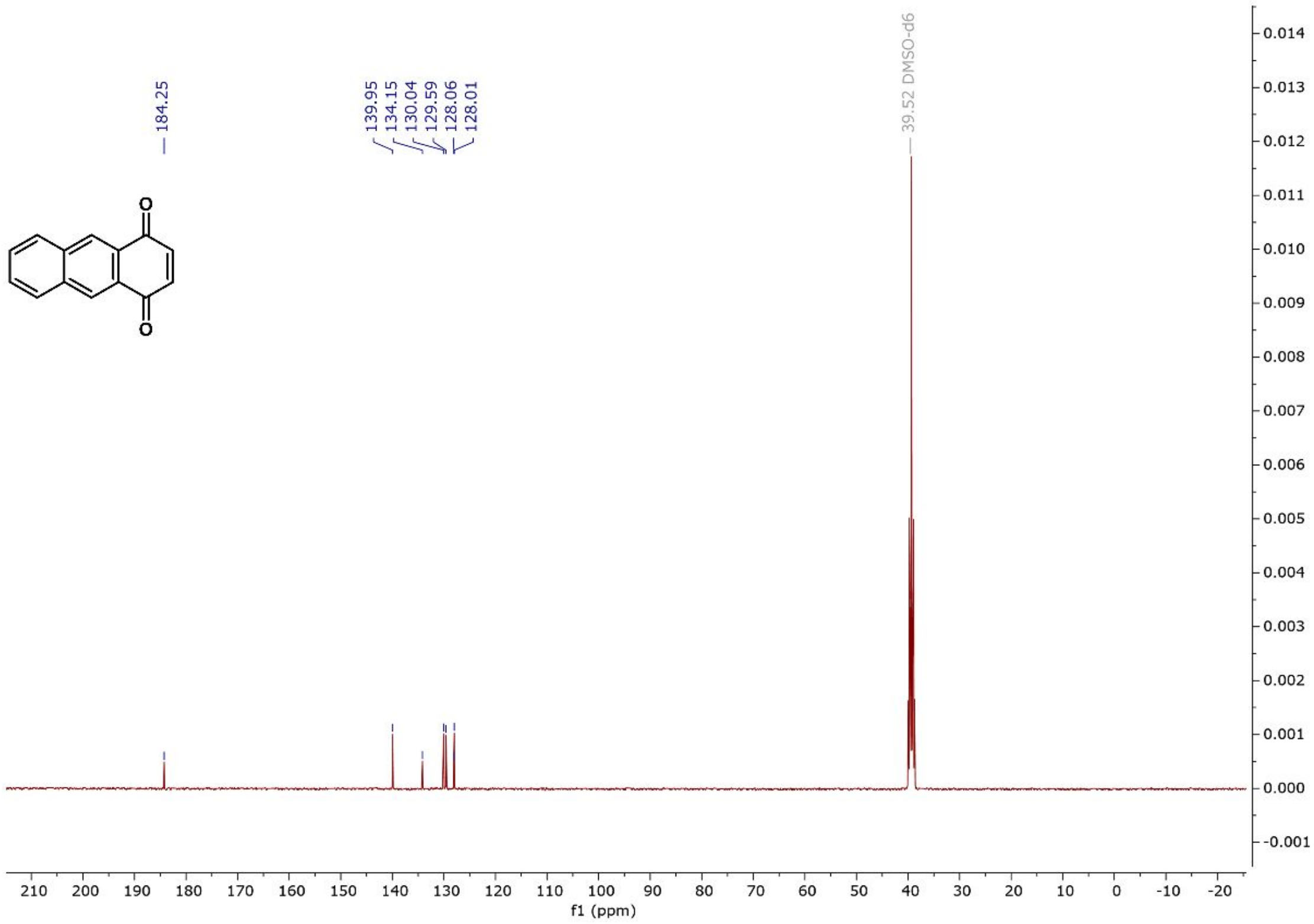

Figure S2. ^{13}C-NMR spectrum of 1,4-anthraquinone (compound B) in DMSO-d_6 at 298 K

Compound C

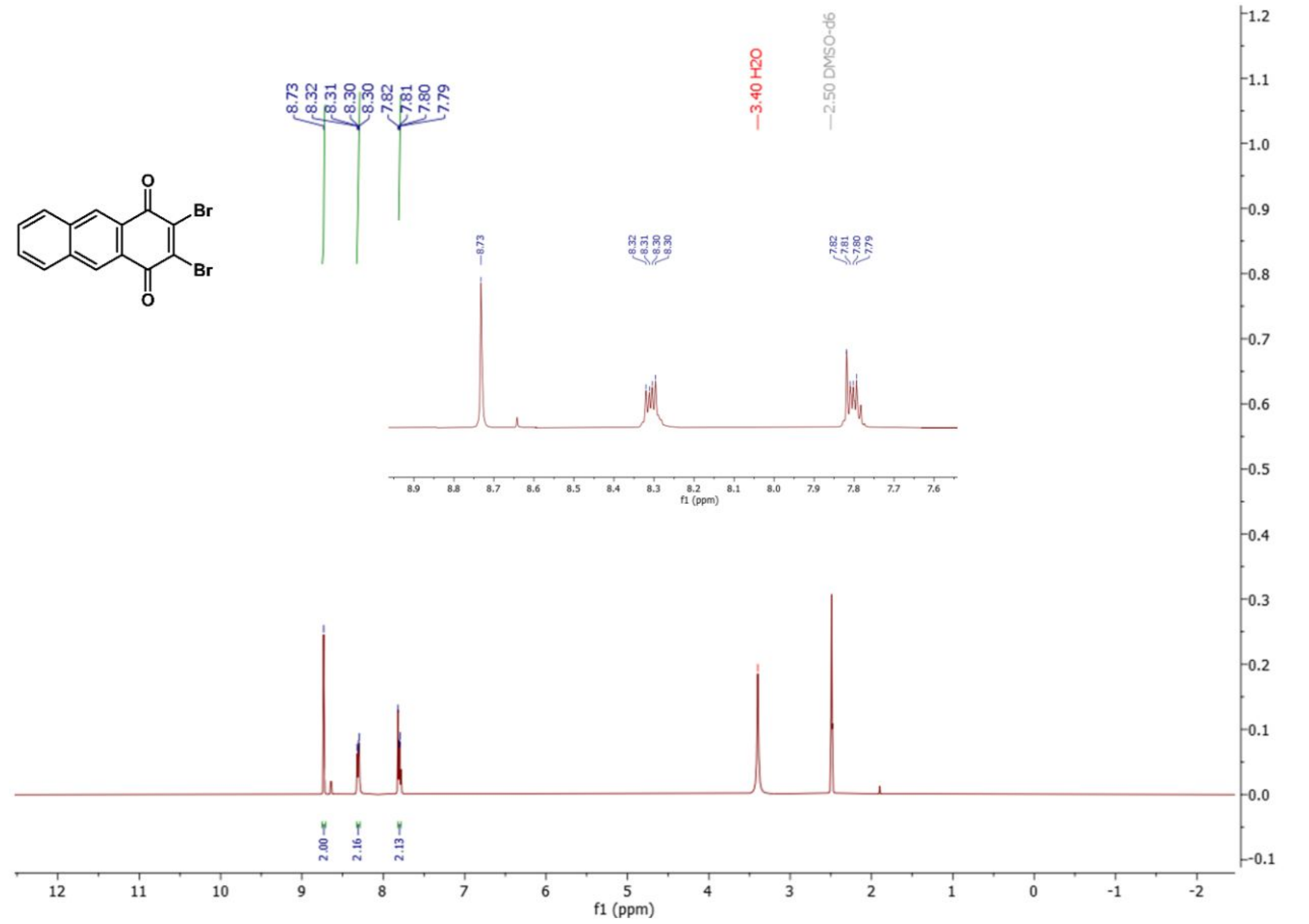

Figure S3. ^{1}H-NMR spectrum of 2,3-dibromoanthraquinone (compound C) in DMSO-d_6 at 298 K

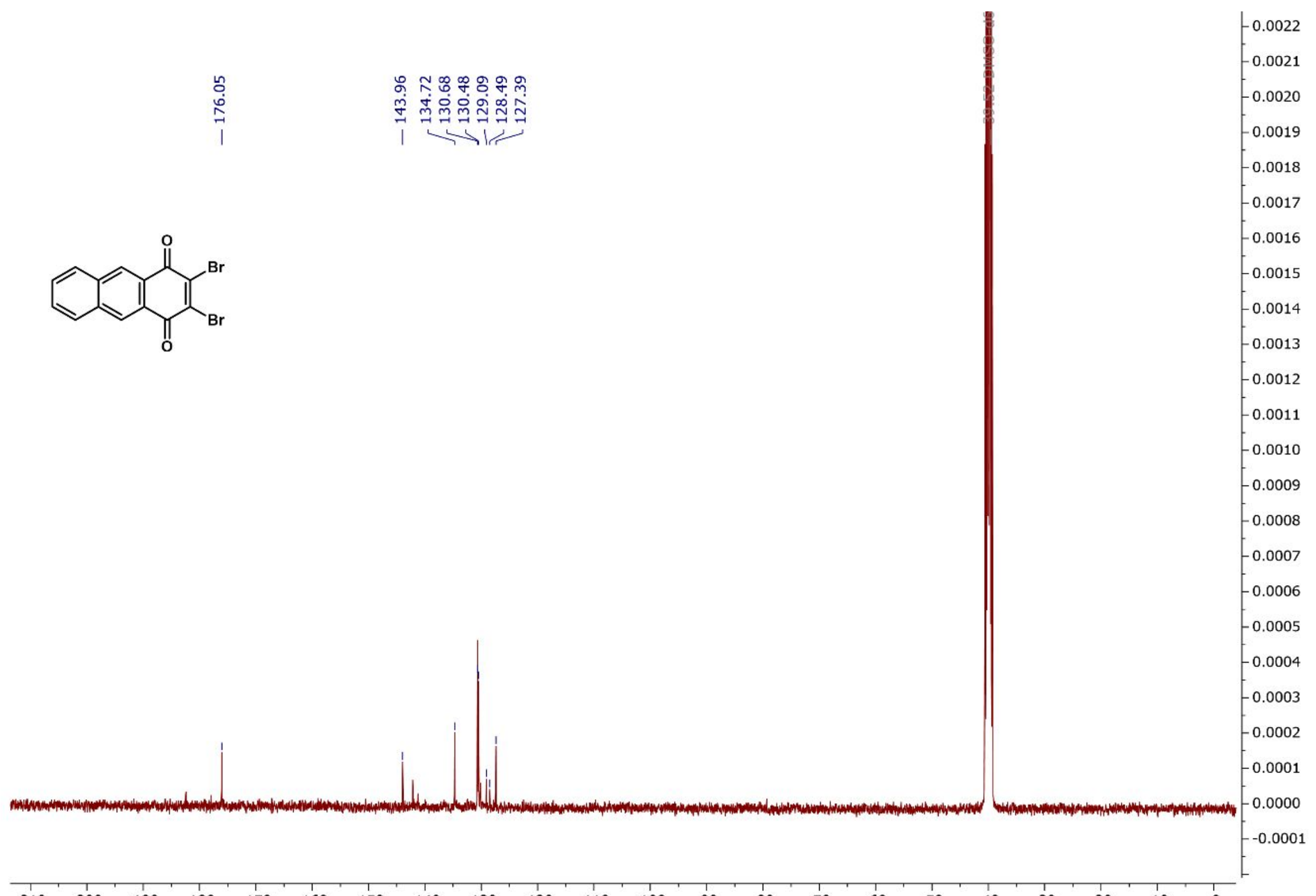

Figure S4. ^{13}C-NMR spectrum of 2,3-dibromoanthraquinone (compound C) in DMSO-d_6 at 298 K

Compound D

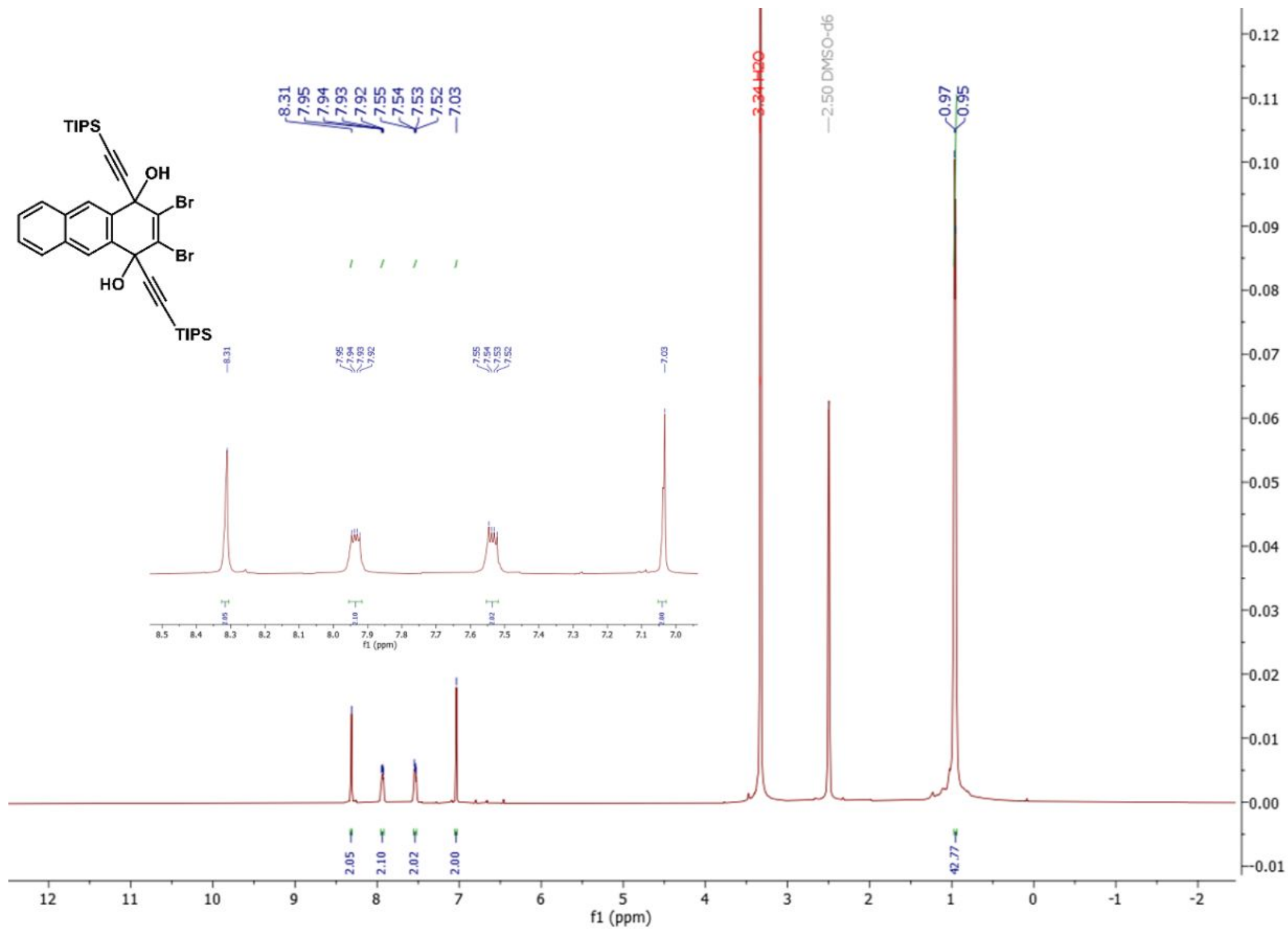

Figure S5. ^{1}H-NMR spectrum of 2,3-dibromo-1,4-bis(triisopropylsilylethynyl)-1,4-dihydroanthracene-1,4-diol (compound D) in DMSO-d_6 at 298 K

Compound E

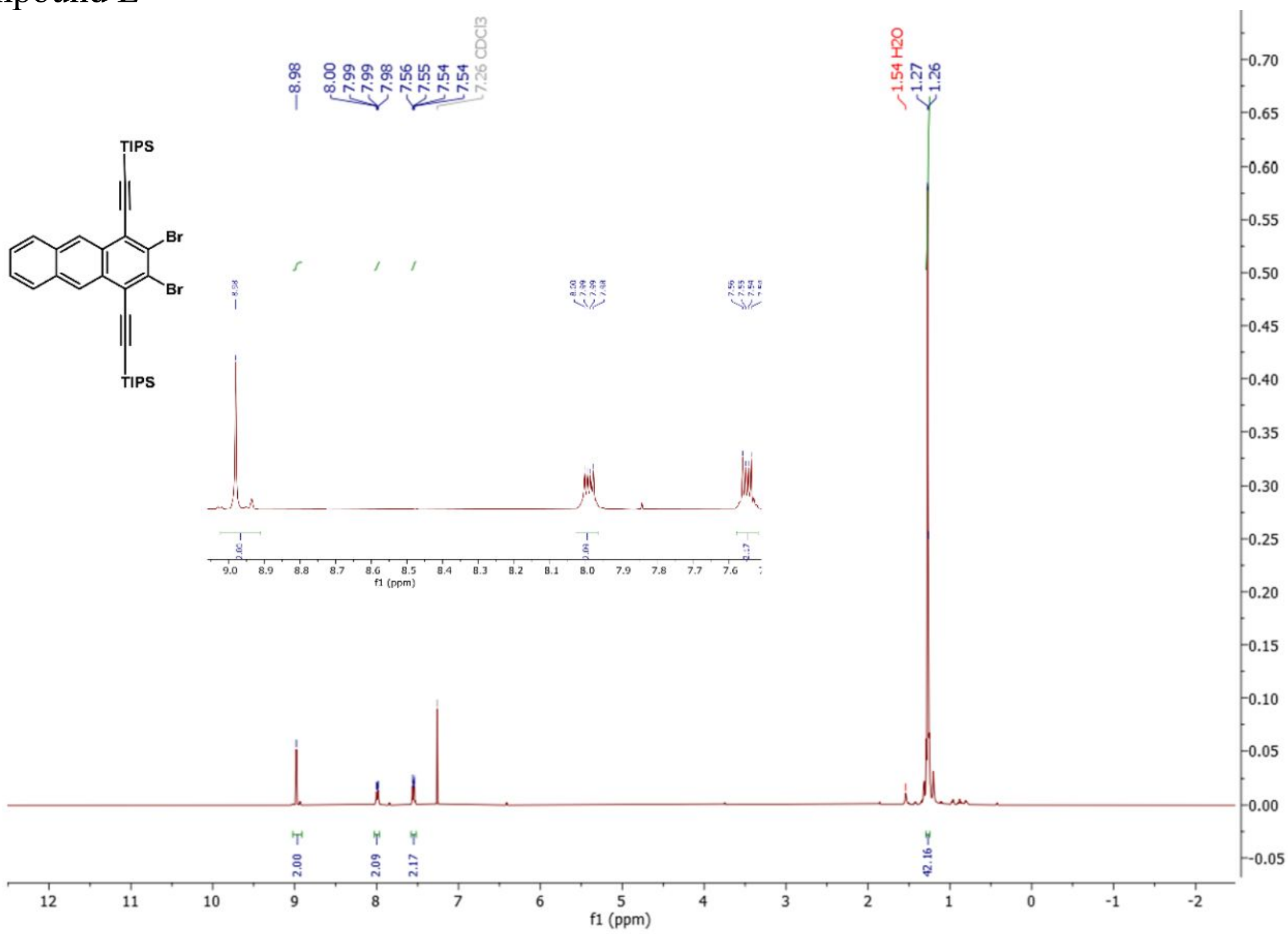

Figure S6. ^{1}H-NMR spectrum of 2,3-dibromo-1,4-bis(triisopropylsilylethynyl)-anthracene (compound E) in $CDCl_3$ at 298 K

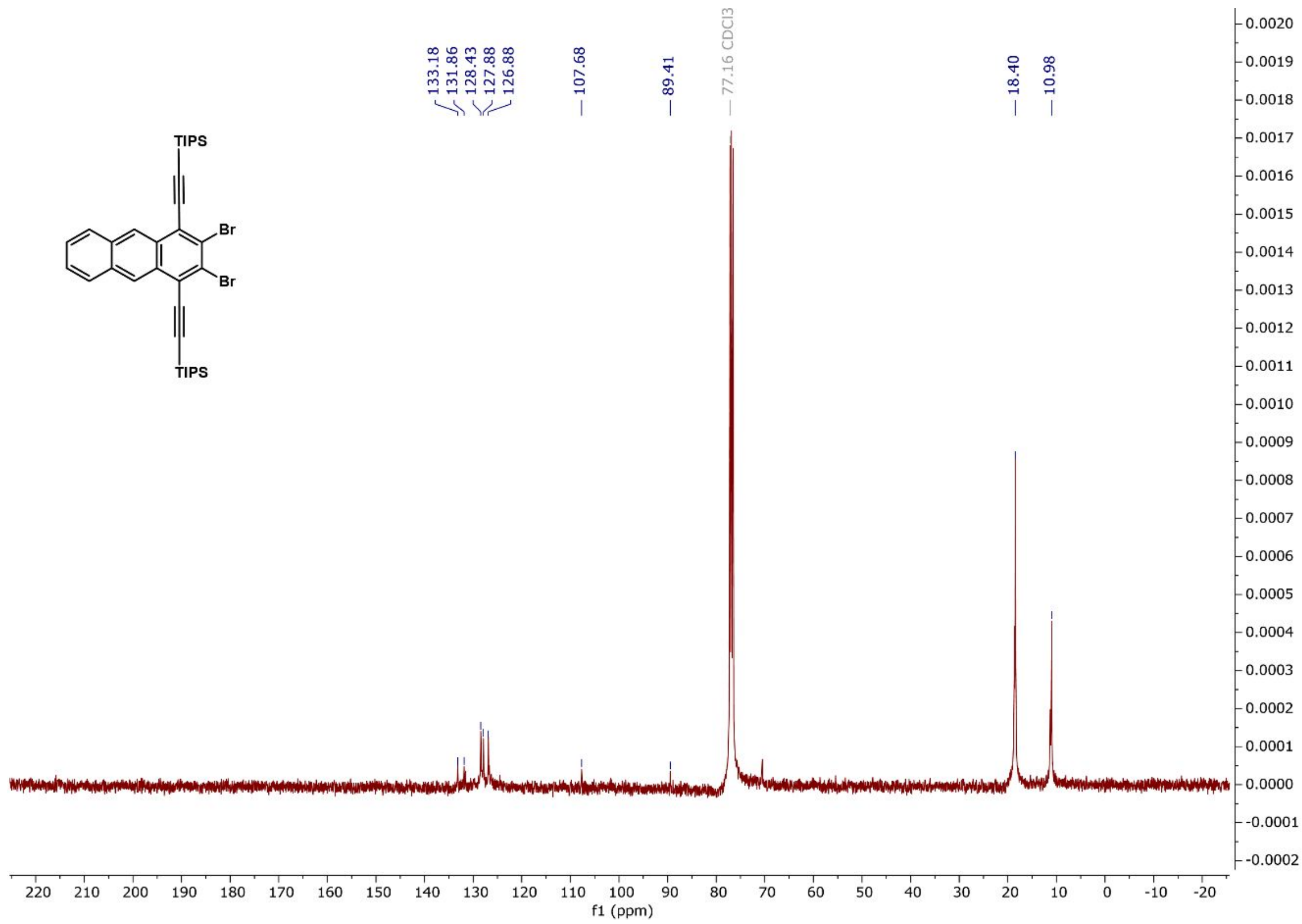

Figure S7. ^{13}C-NMR spectrum of 2,3-dibromo-1,4-bis(triisopropylsilylethynyl)-anthracene (compound E) in $CDCl_3$ at 298 K

Compound F

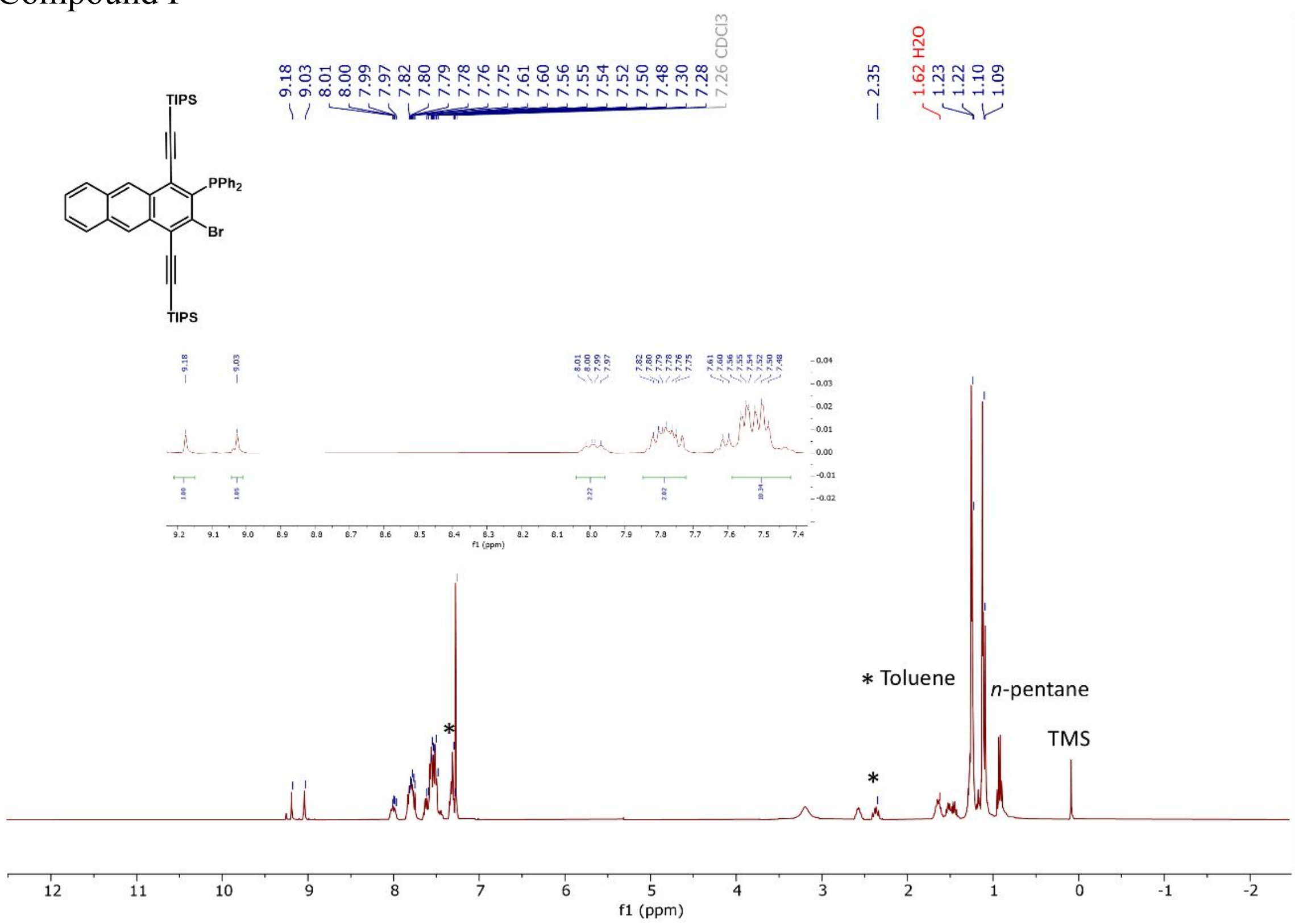

Figure S8. ^{1}H-NMR spectrum of 3-bromo-2-diphenylphosphino-1,4-bis(triisopropylsilylethynyl) anthracene (compound F) in $CDCl_3$ at 298 K

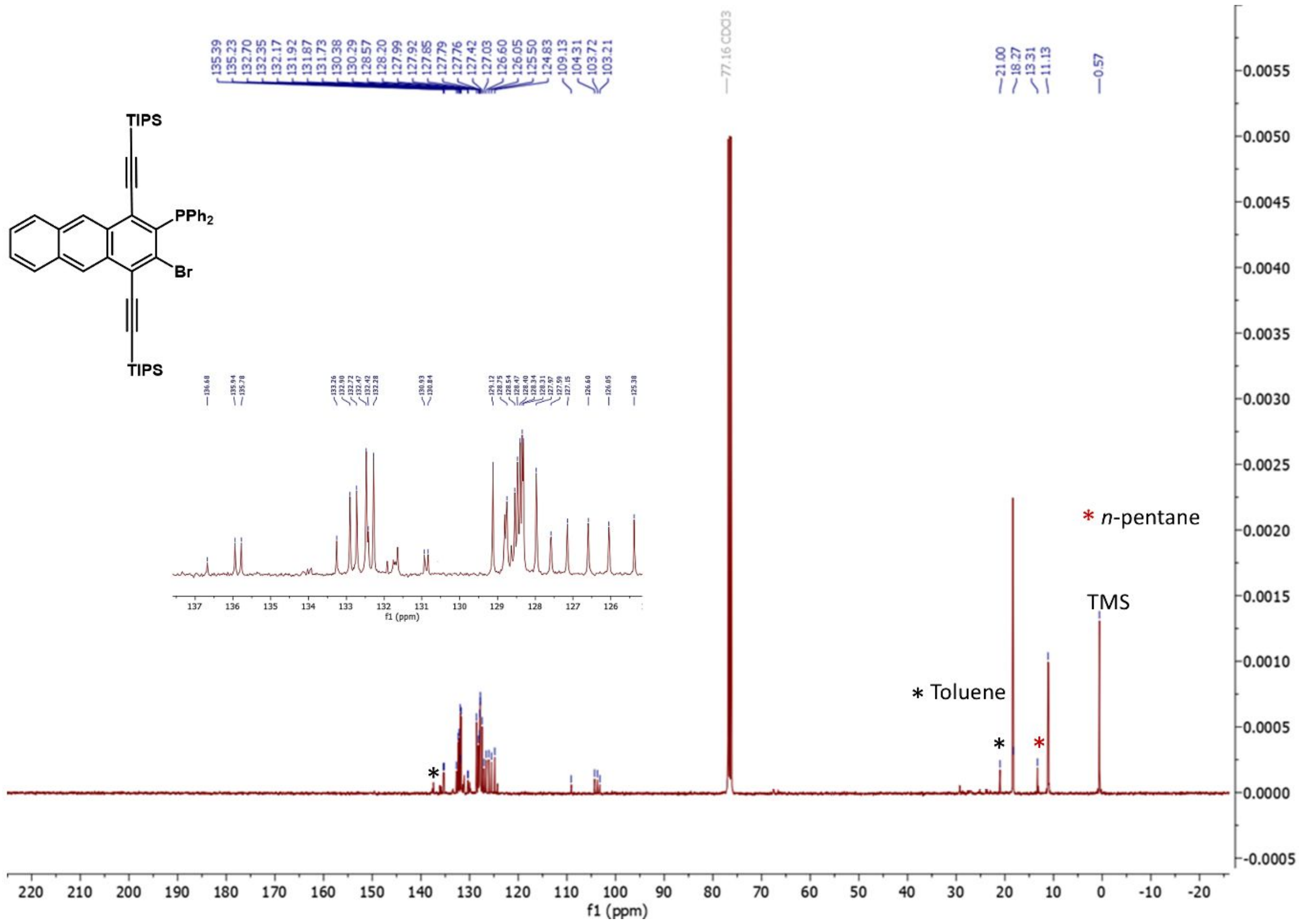

Figure S9. ^{13}C-NMR spectrum of 3-bromo-2-diphenylphosphino-1,4-bis(triisopropylsilylethynyl) anthracene (compound F) in $CDCl_3$ at 298 K

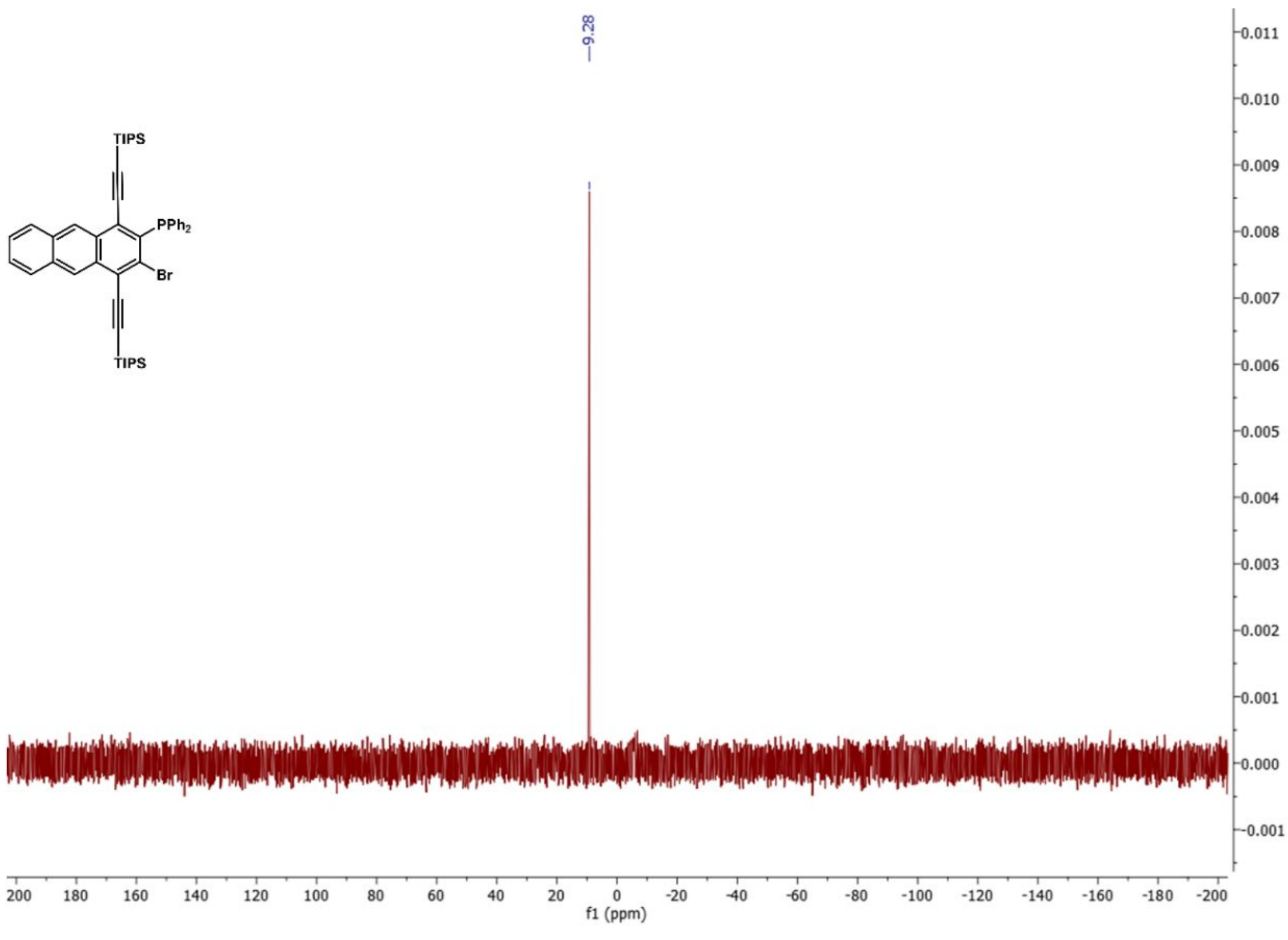

Figure S10. ^{31}P-NMR spectrum of 3-bromo-2-diphenylphosphino-1,4-bis(triisopropylsilylethynyl) anthracene (compound F) in $CDCl_3$ at 298 K

Compound G

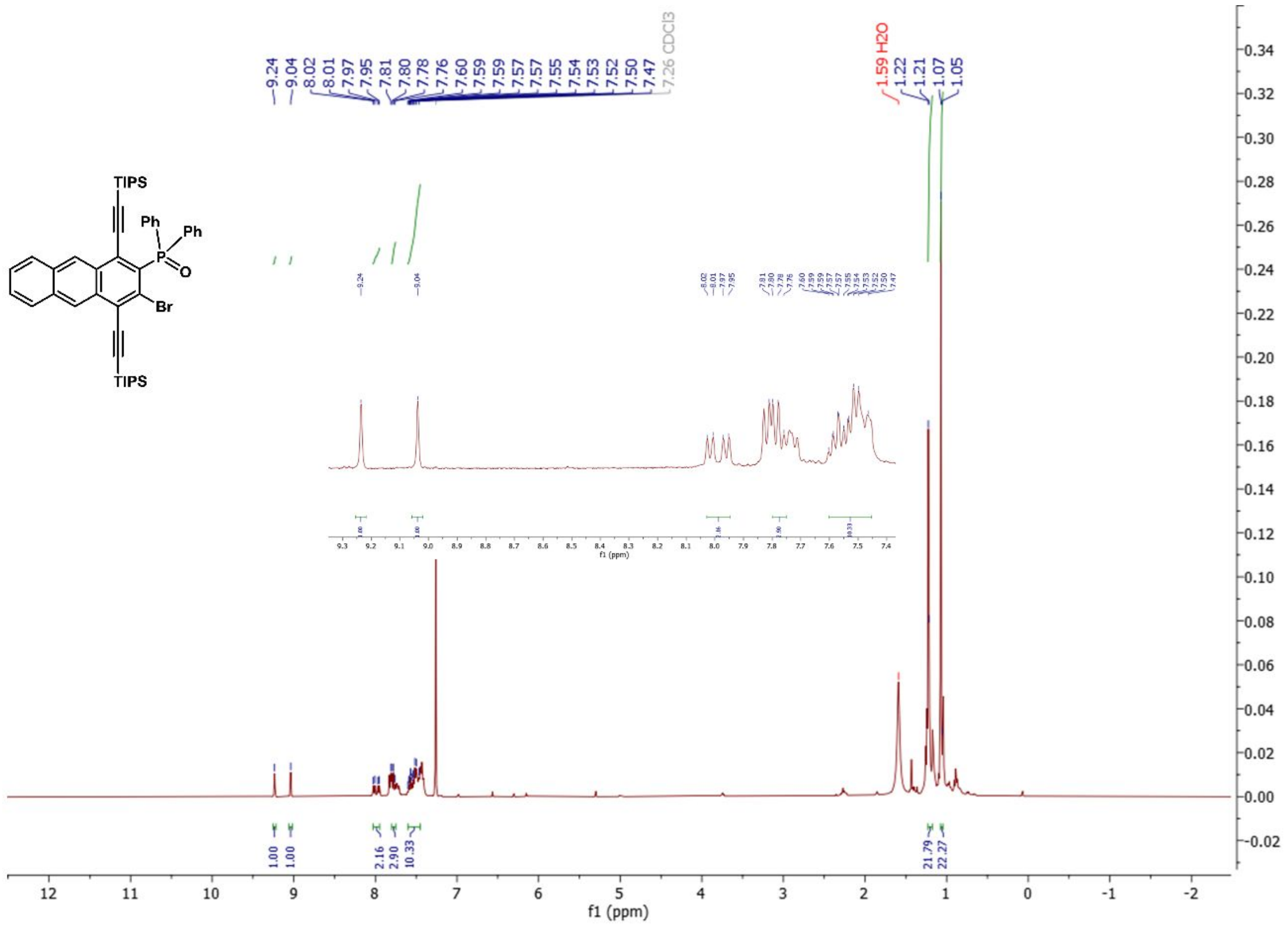

Figure S11. ^{1}H-NMR spectrum of 3-Bromo-2-diphenylphosphineoxide-1,4-bis (triisopropylsilylethynyl) anthracene (compound G; TIPS-AntPO) in $CDCl_3$ at 298 K

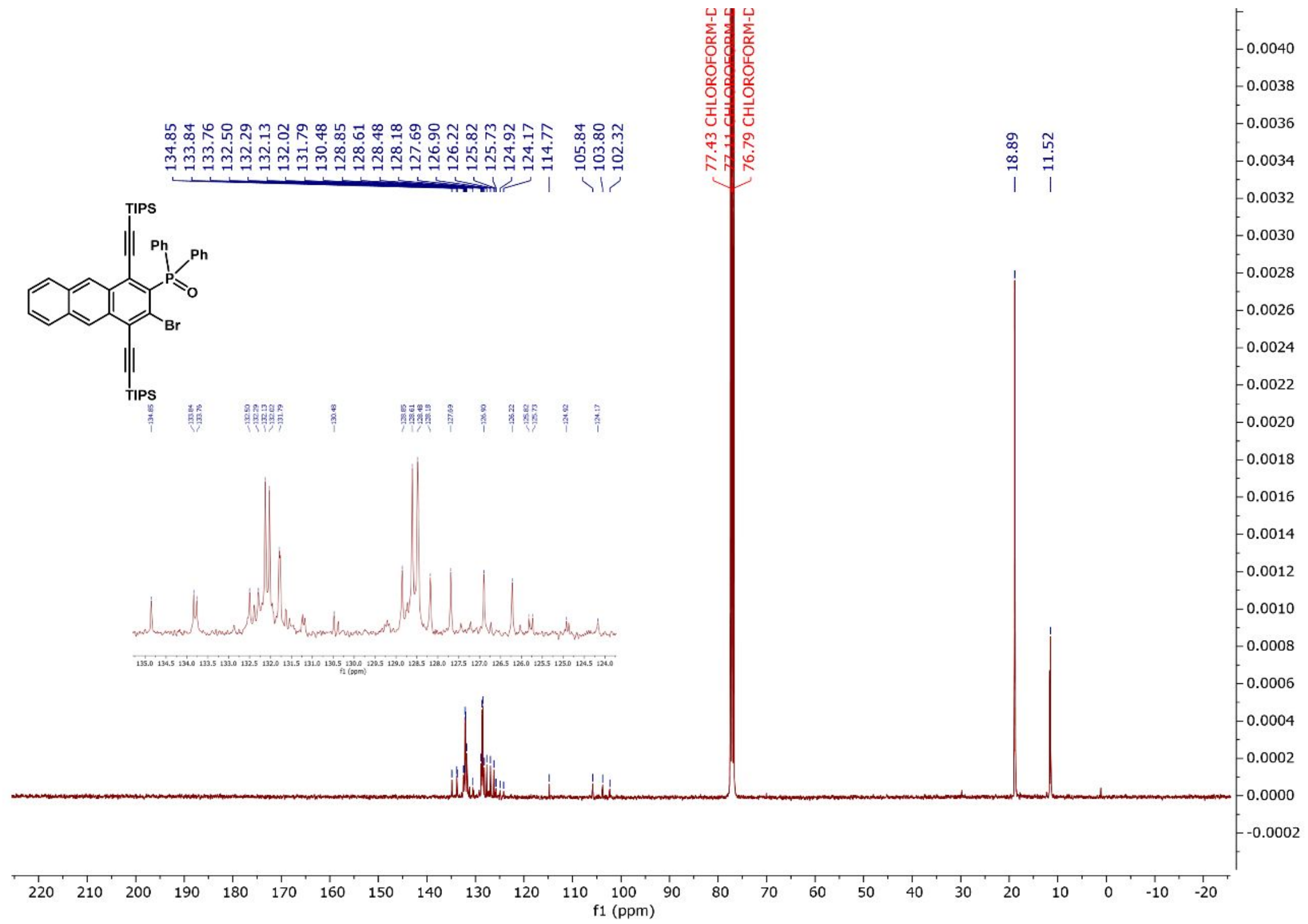

Figure S12. ^{13}C-NMR spectrum of 33-Bromo-2-diphenylphosphineoxide-1,4-bis (triisopropylsilylethynyl) anthracene (compound G; TIPS-AntPO) in $CDCl_3$ at 298 K

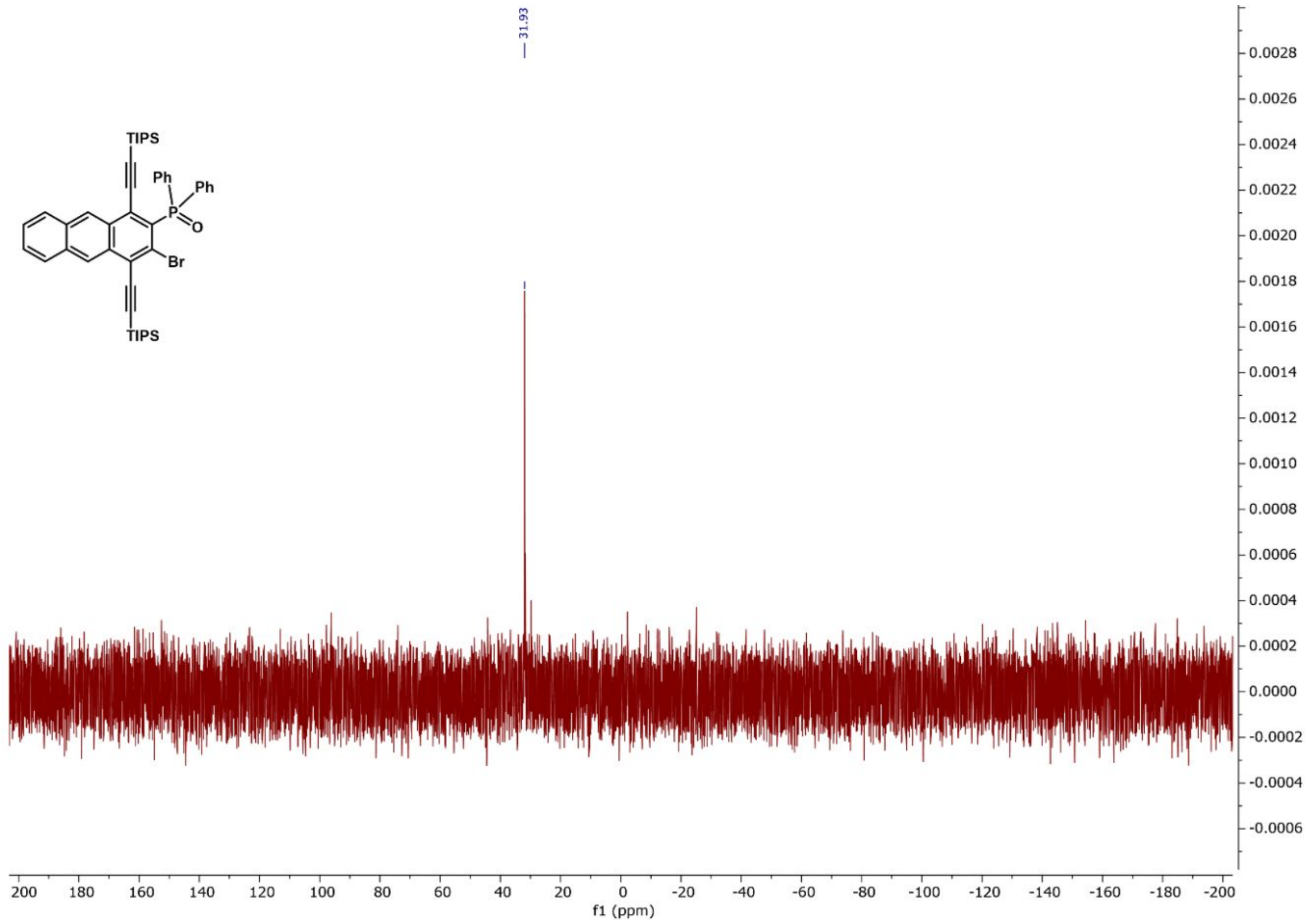

Figure S13. ^{31}P-NMR spectrum of 3-Bromo-2-diphenylphosphineoxide-1,4-bis (triisopropylsilylethynyl) anthracene (compound G; TIPS-AntPO) in $CDCl_3$ at 298 K

Compound H

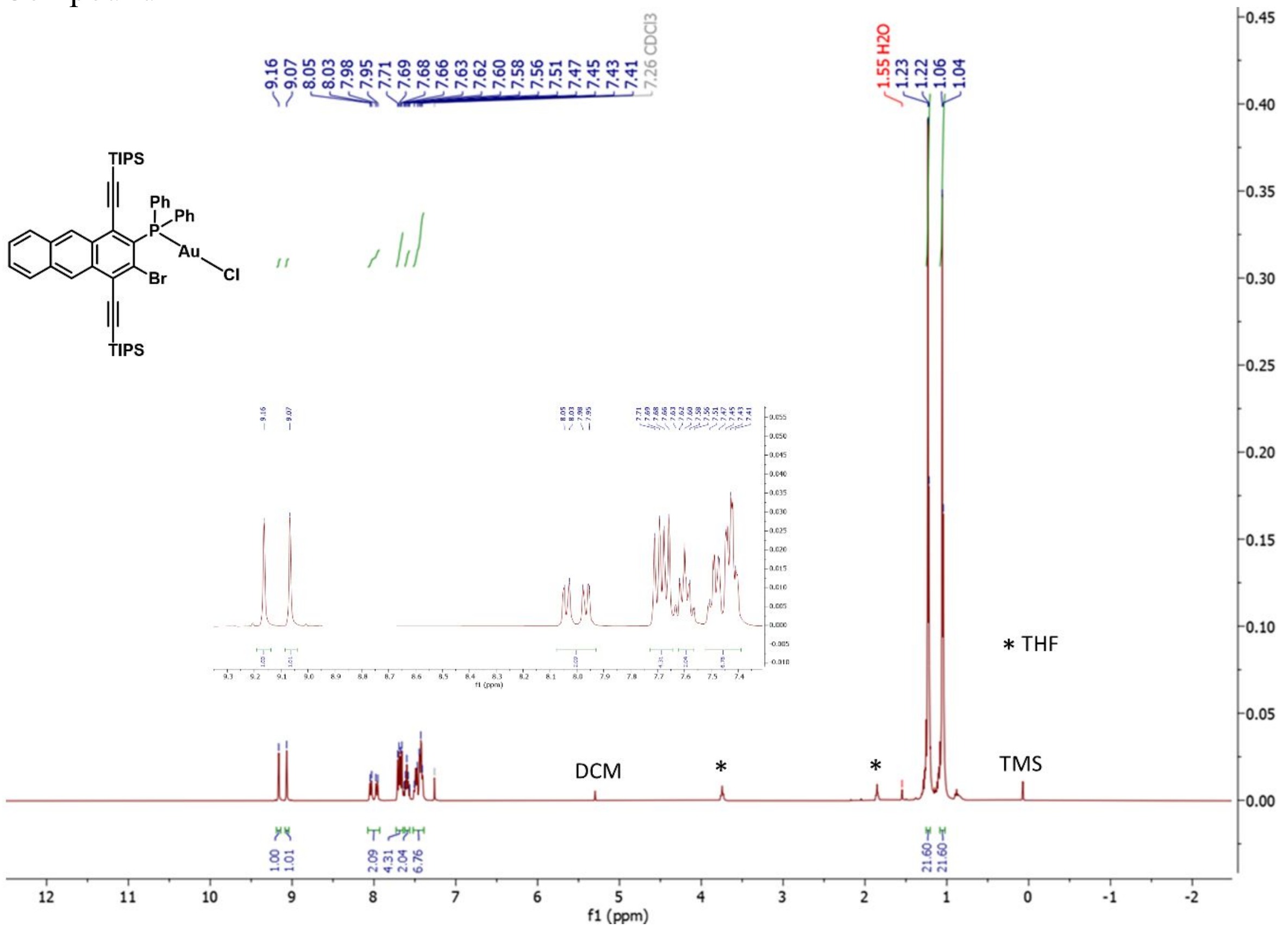

Figure S14. ^{1}H-NMR spectrum of compound H [Au(TIPS-AntP)Cl)] in $CDCl_3$ at 298 K

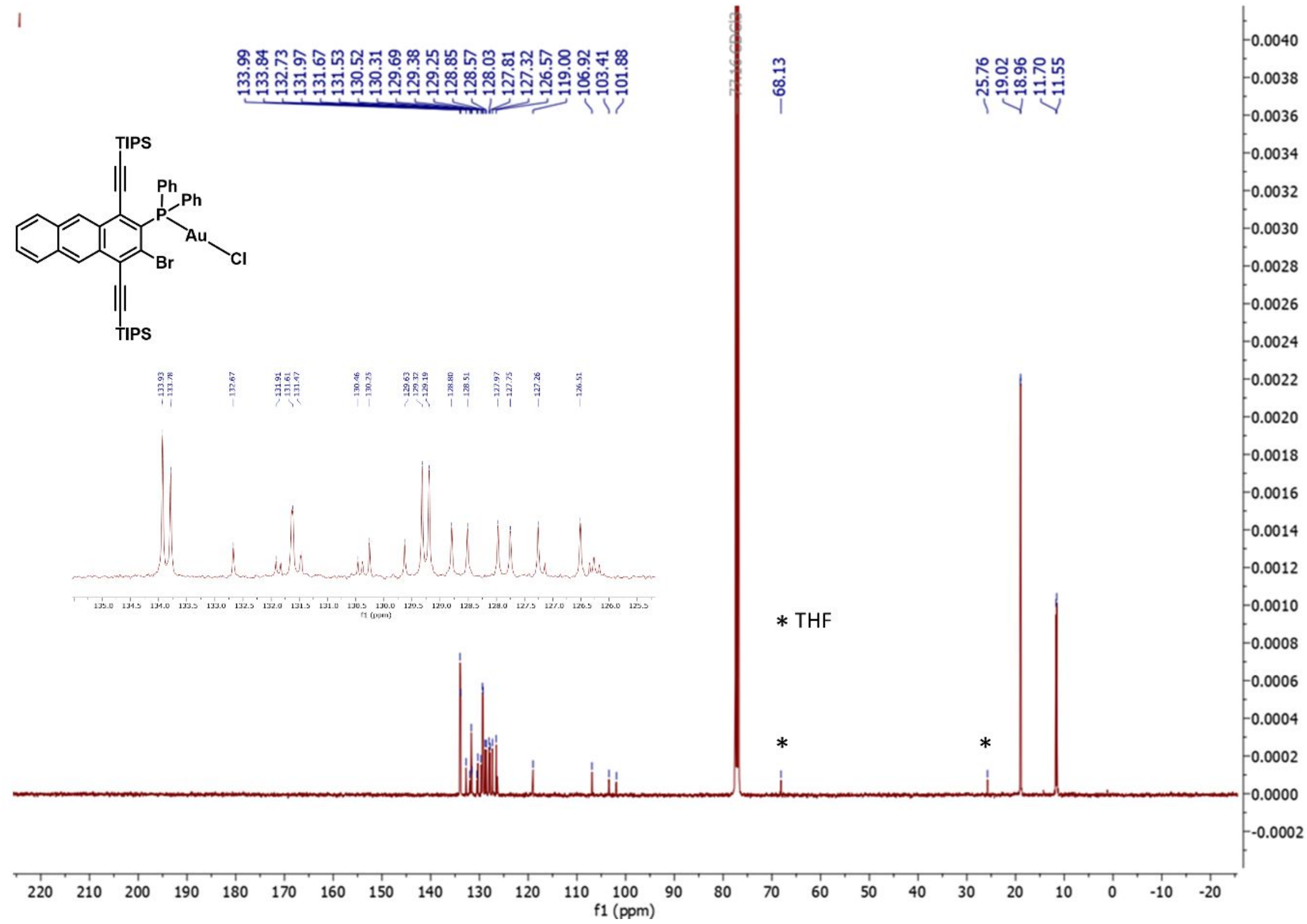

Figure S15. ^{13}C-NMR spectrum of compound H [Au(TIPS-AntP)Cl)] in $CDCl_3$ at 298 K

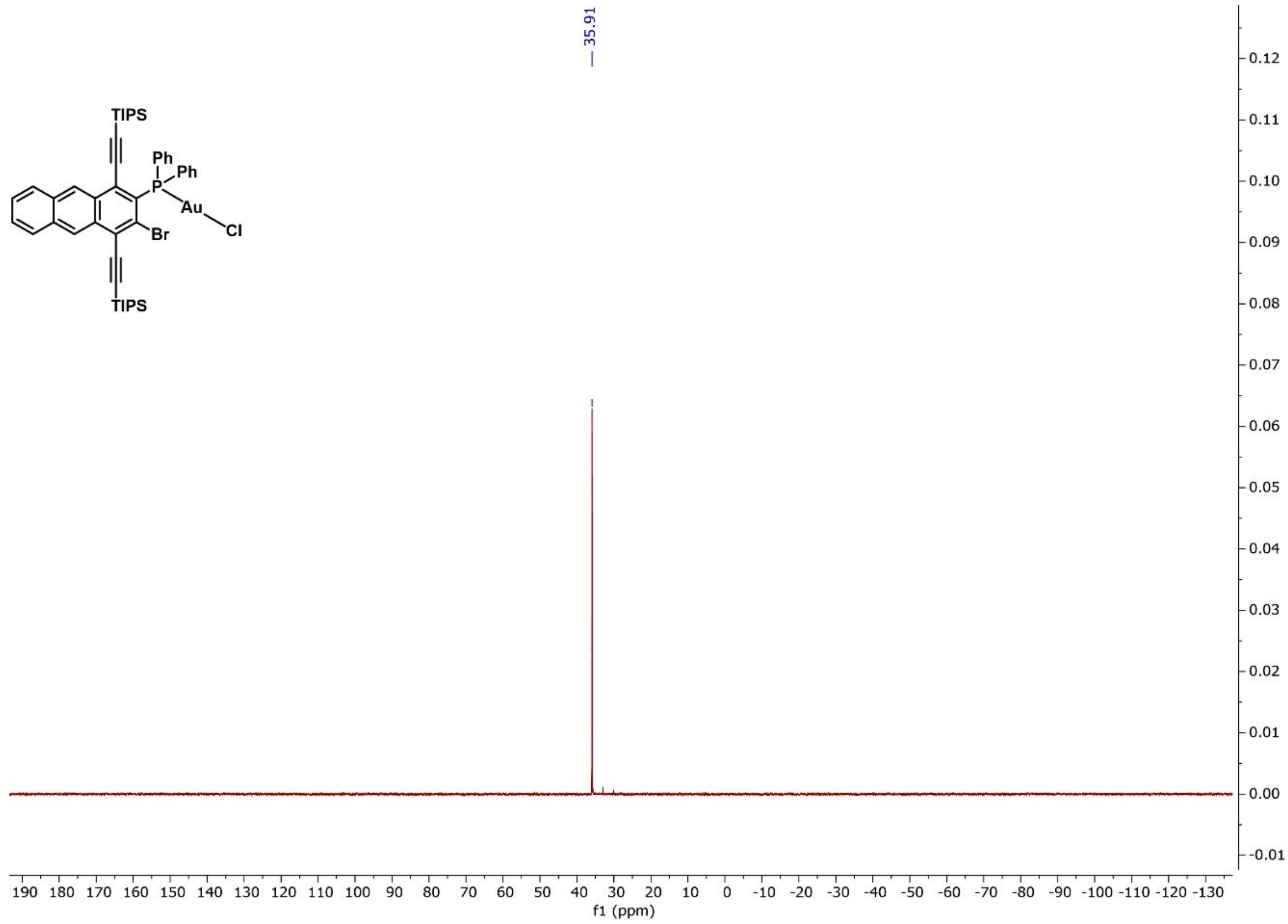

Figure S16. ^{31}P-NMR spectrum of compound H [Au(TIPS-AntP)Cl)] in $CDCl_3$ at 298 K

Compound I

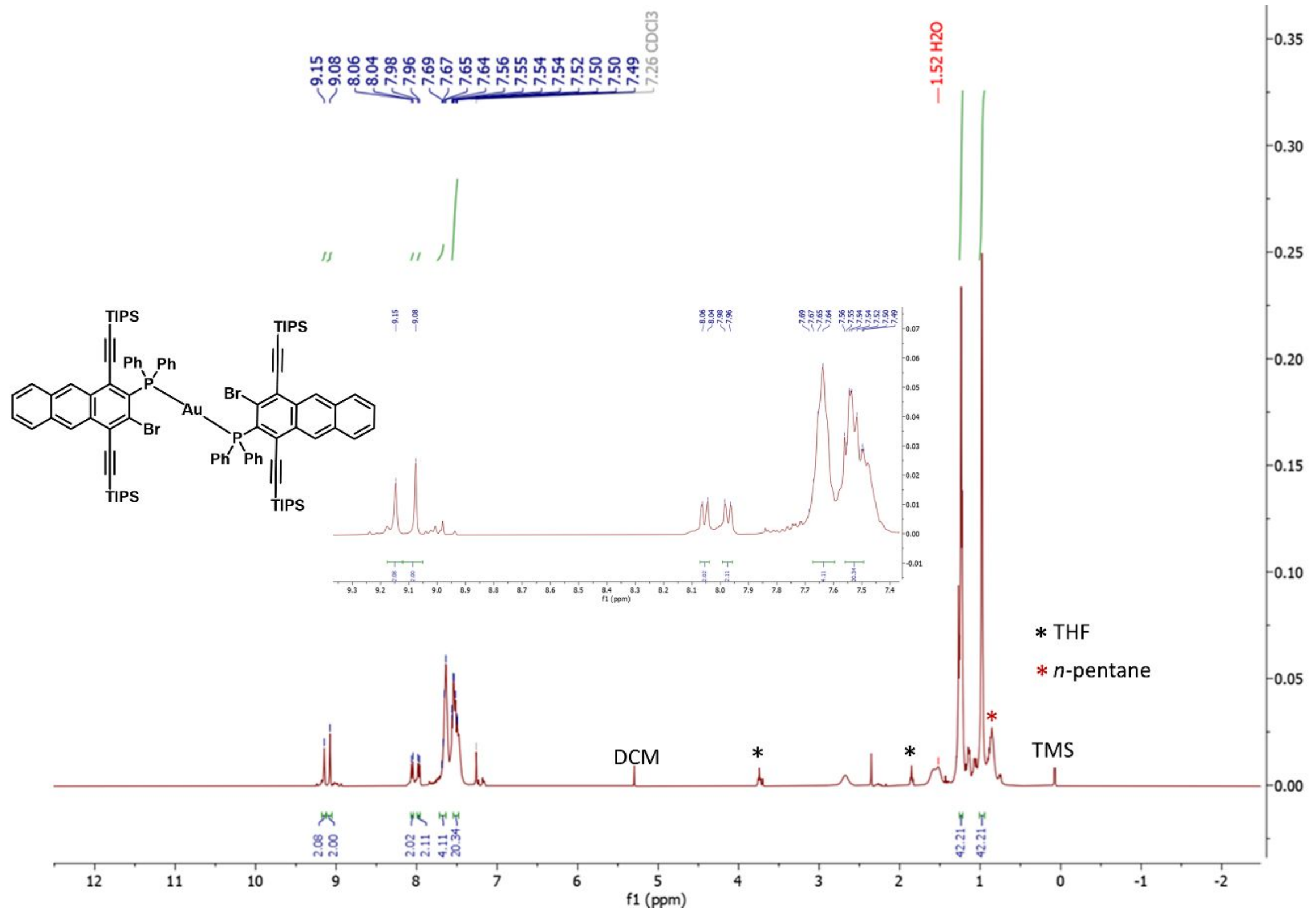

Figure S17. ^{1}H-NMR spectrum of compound I [Au(TIPS-AntP)$_2$)OTf] in $CDCl_3$ at 298 K

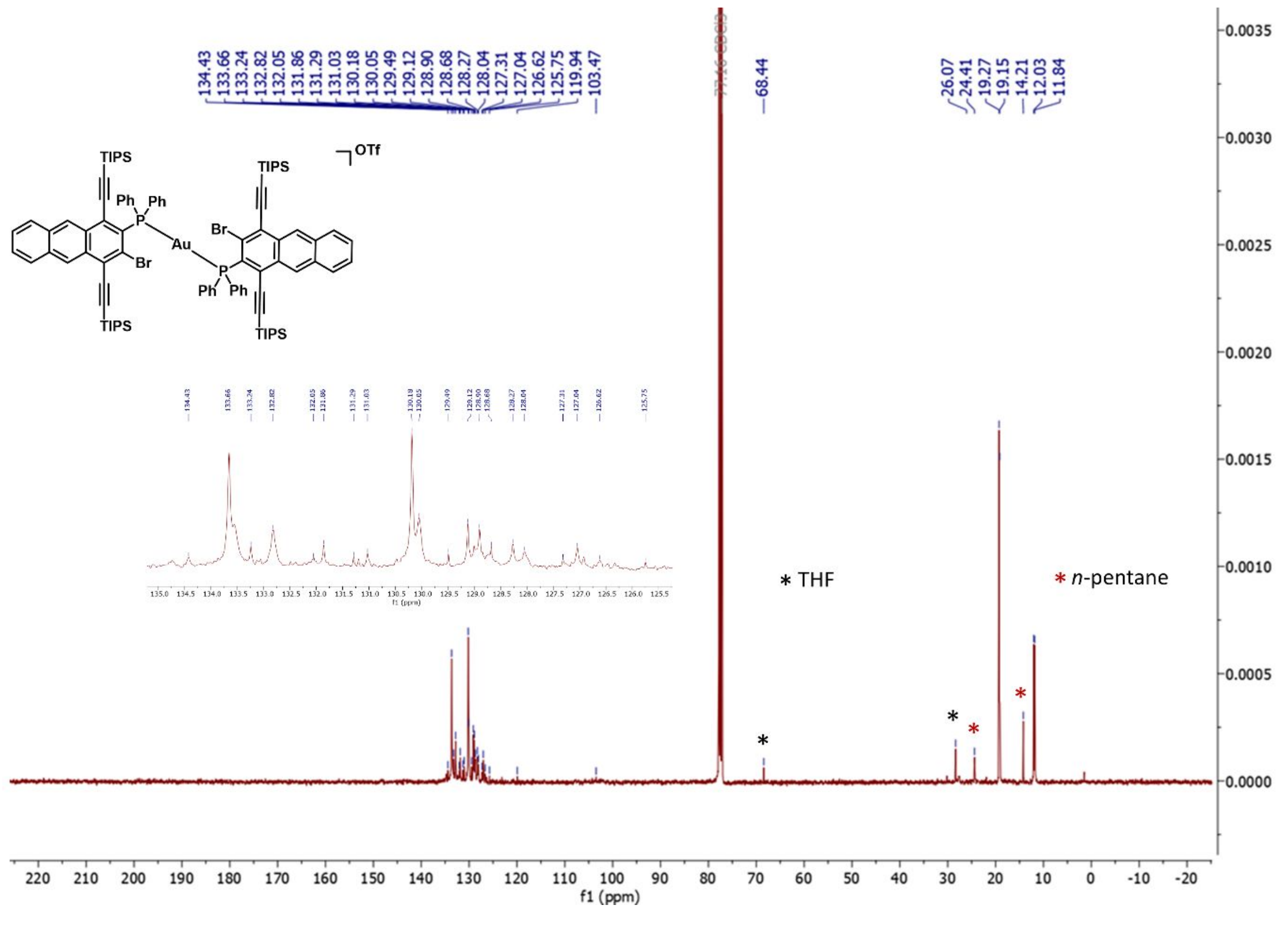

Figure S18. ^{13}C-NMR spectrum of compound I [Au(TIPS-AntP)$_2$)OTf] in $CDCl_3$ at 298 K

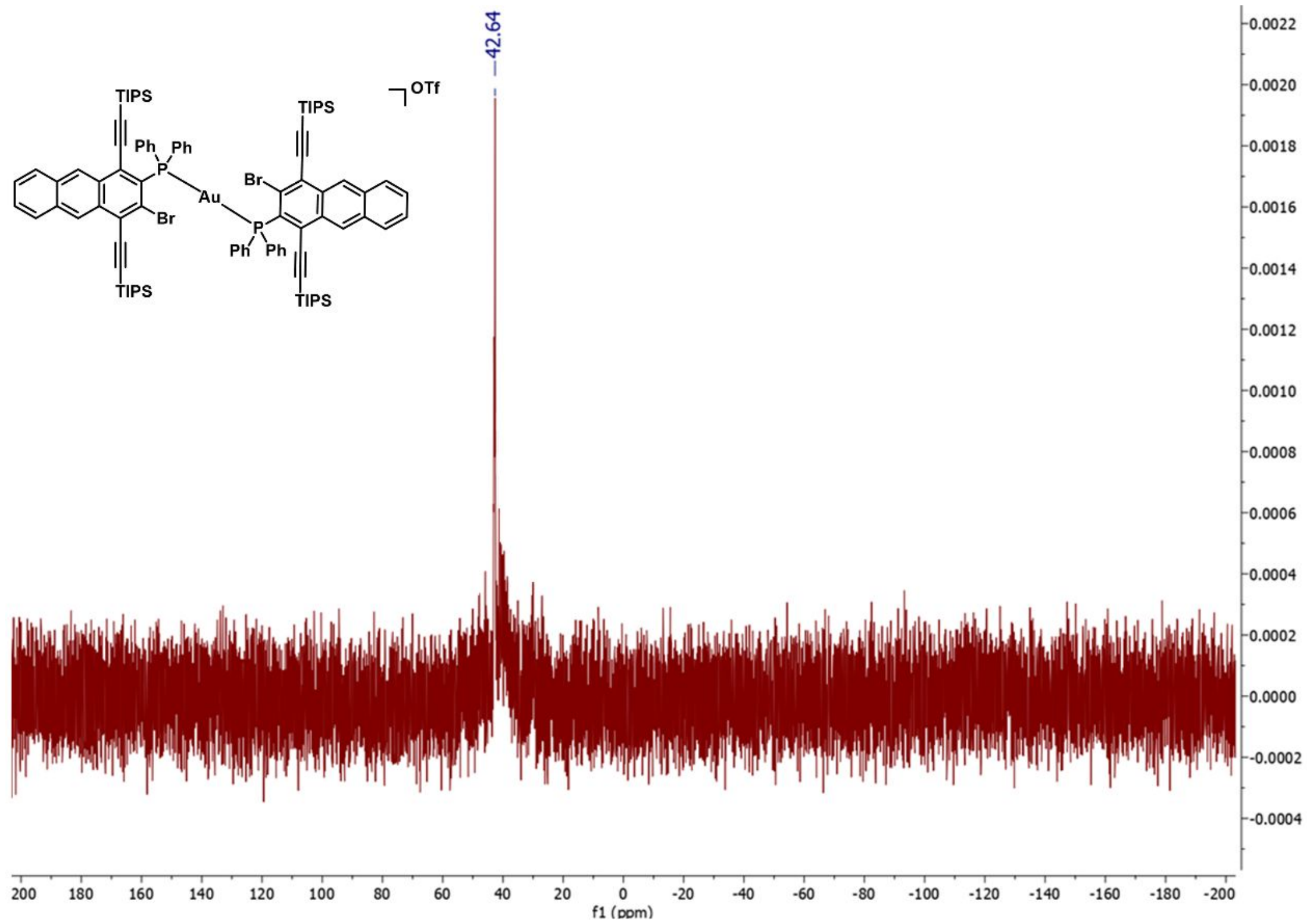

Figure S19. ^{31}P-NMR spectrum of compound I [Au(TIPS-AntP)$_2$)OTf] in $CDCl_3$ at 298 K

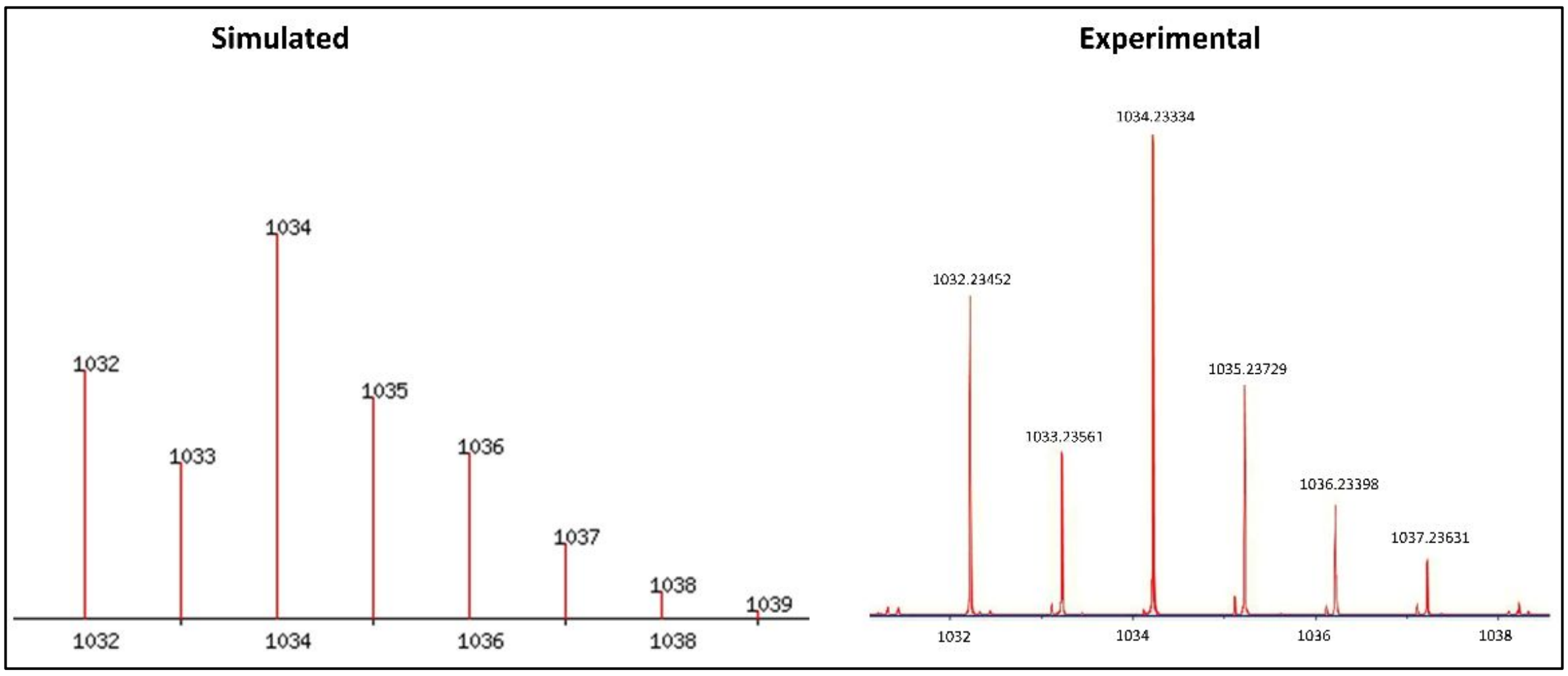

Figure S20. Experimental and simulated isotopic distribution patterns for compound H [Au(TIPS-AntP)Cl)] at m/z = 1034.23

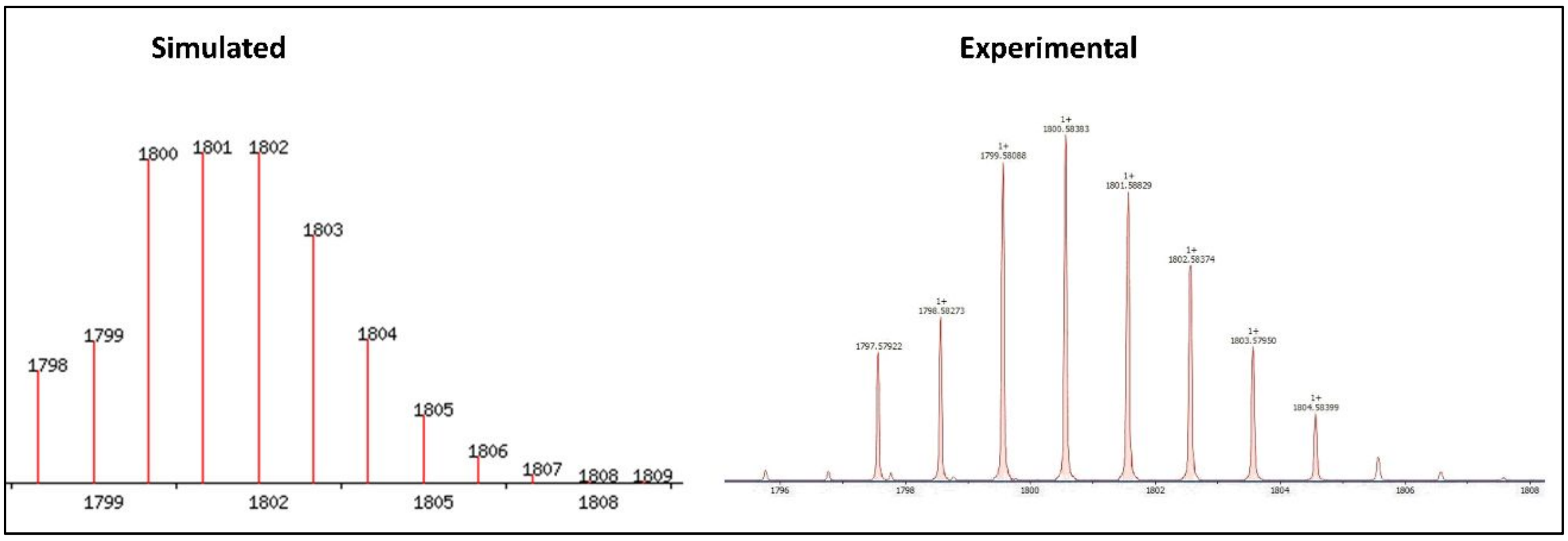

Figure S21. Experimental and simulated isotopic distribution patterns for compound I [Au(TIPS-AntP)$_2$)OTf] at m/z = 1800.57

4. **Table S1** Crystallographic data and structure refinement details of **TIPS-AntPO** and **[Au(TIPS-AntP)Cl)]**

**Parameters**	**TIPS-AntPO**	**[Au(TIPS-AntP)Cl)]**
CCDC No.	2491607	2491609
Formula	$C_{48}H_{58}BrOPSi_2$	$C_{48}H_{58}AuBrClPSi_2$
F.W. (g mol^{-1})	818.043	1034.42
crystal system	monoclinic	triclinic
space group	P 2_1/n	P$\bar{1}$
a/ Å	14.20969(10)	12.6443(4)
b/ Å	12.45990(9)	13.1320(4)
c/ Å	24.9786(2)	14.5232(4)
α/ deg	90	75.096(3)
β/ deg	97.7284(7)	85.204(3)
γ/ deg	90	81.476(3)
V/ Å^3	4382.33(6)	2302.02(12)
Z	4	2
Limiting indices	$-18 \leq h \leq 18$<br>$-13 \leq k \leq 15$<br>$-31 \leq l \leq 31$	$-16 \leq h \leq 11$<br>$-16 \leq k \leq 16$<br>$-18 \leq l \leq 18$
D_c/g cm^{-3}	1.240	1.492
2θ range	3.3780-78.8600	3.1420-79.3920
μ (mm^{-1})	2.398	8.611
F(000)	1728	1040

T/K	150	150
Total reflections	36813	32991
R(int)	0.0261	0.0669
Unique reflections	9103	9727
Observed reflections	8584	9456
Parameters	490	500
R_1; wR_2 $(I > 2\sigma(I))$	0.0307, 0.0837	0.0597, 0.1664
GOF (F^2)	1.0346	1.071
Largest diff peak and hole (e Å^{-3})	0.5141, -0.5659	2.619, -3.471

5. Packing diagrams

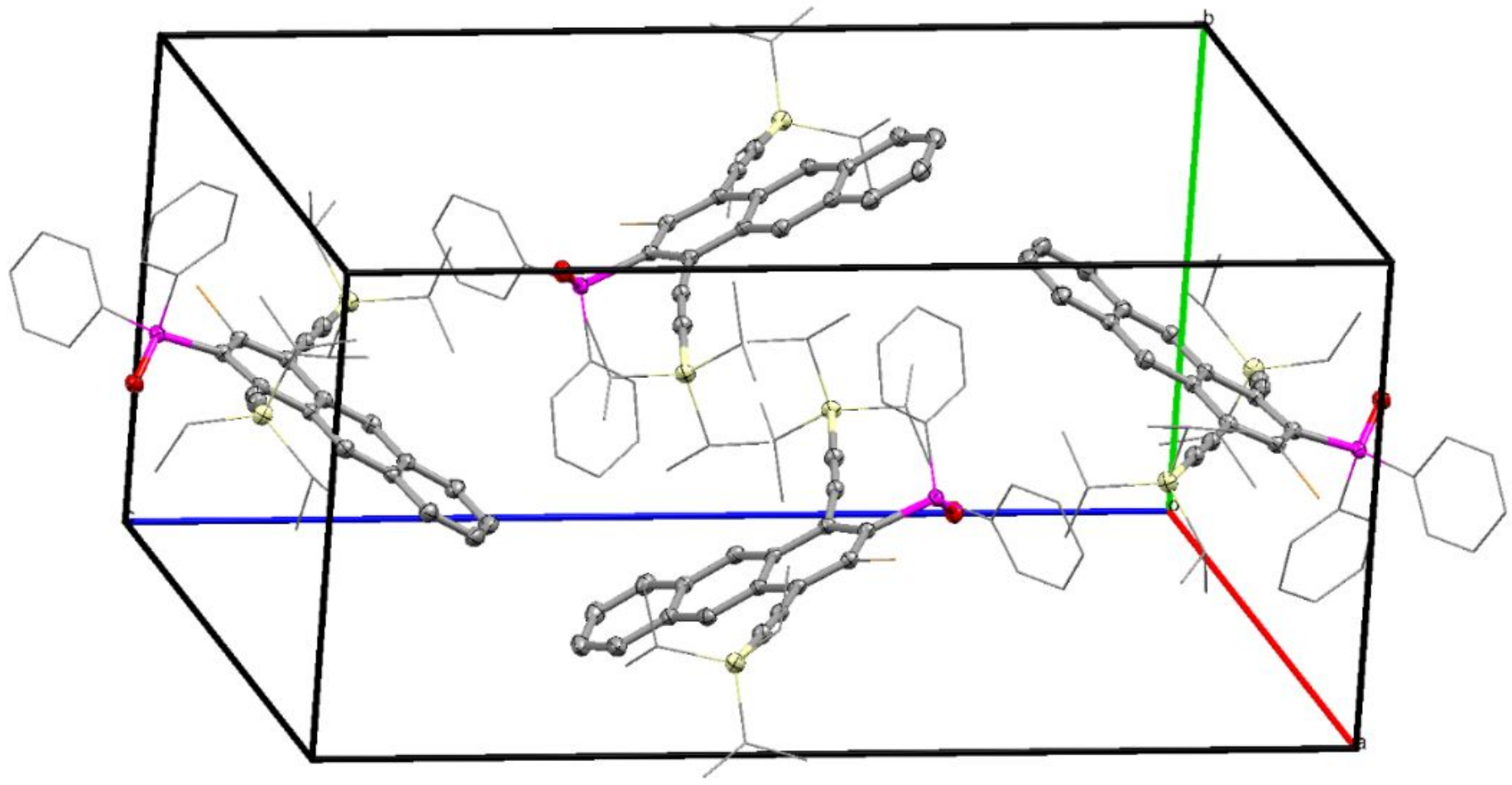

Figure S22. Packing diagram of **TIPS-AntPO**

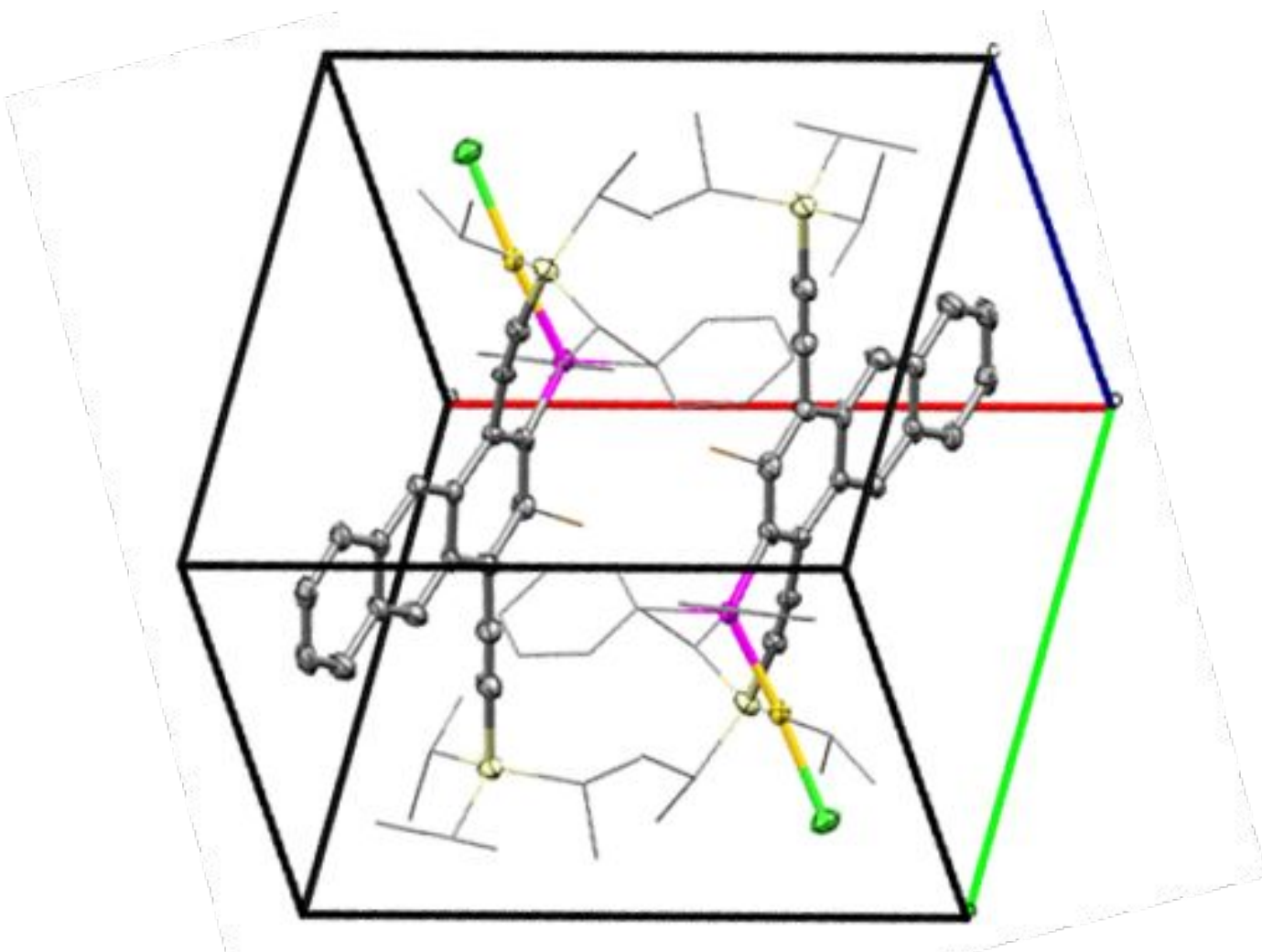

Figure S23. Packing diagram of **[Au(TIPS-AntP)Cl**

6. Photophysical Measurements

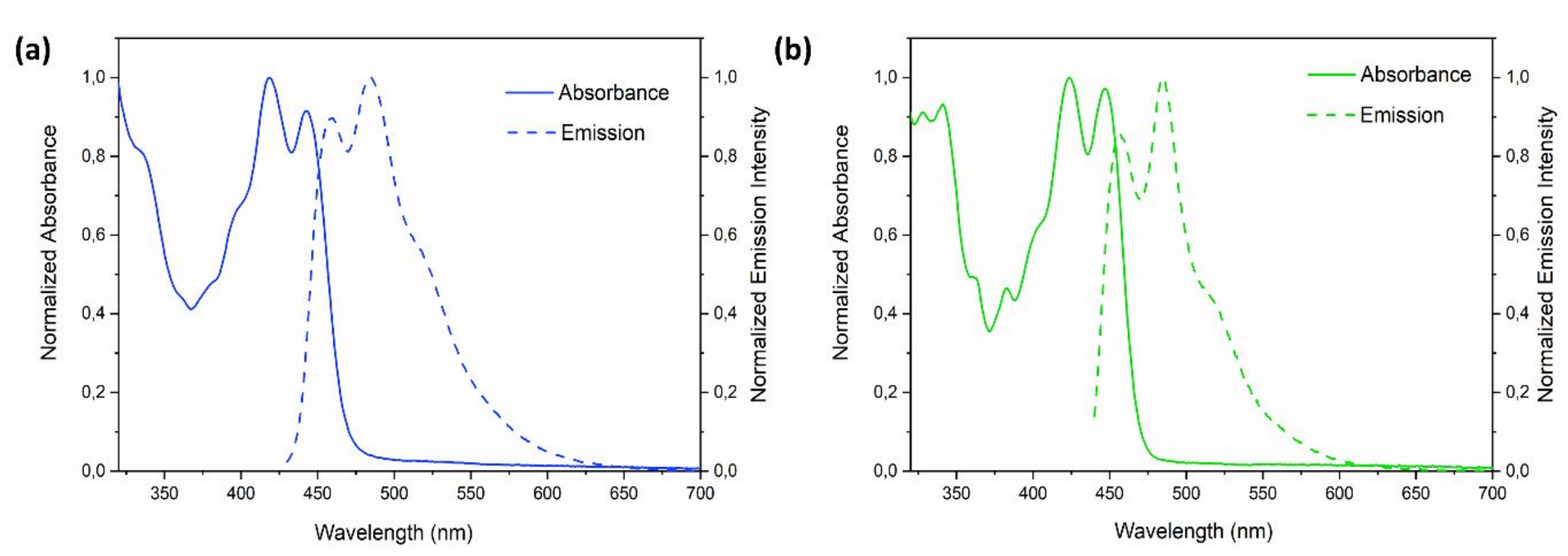

Figure S24. UV−vis absorption (solid) and emission (dashed) spectra of (a) TIPS-AntP and (b) TIPS-AntPO (λ_{exc}= 430 nm) recorded in degassed toluene at room temperature.

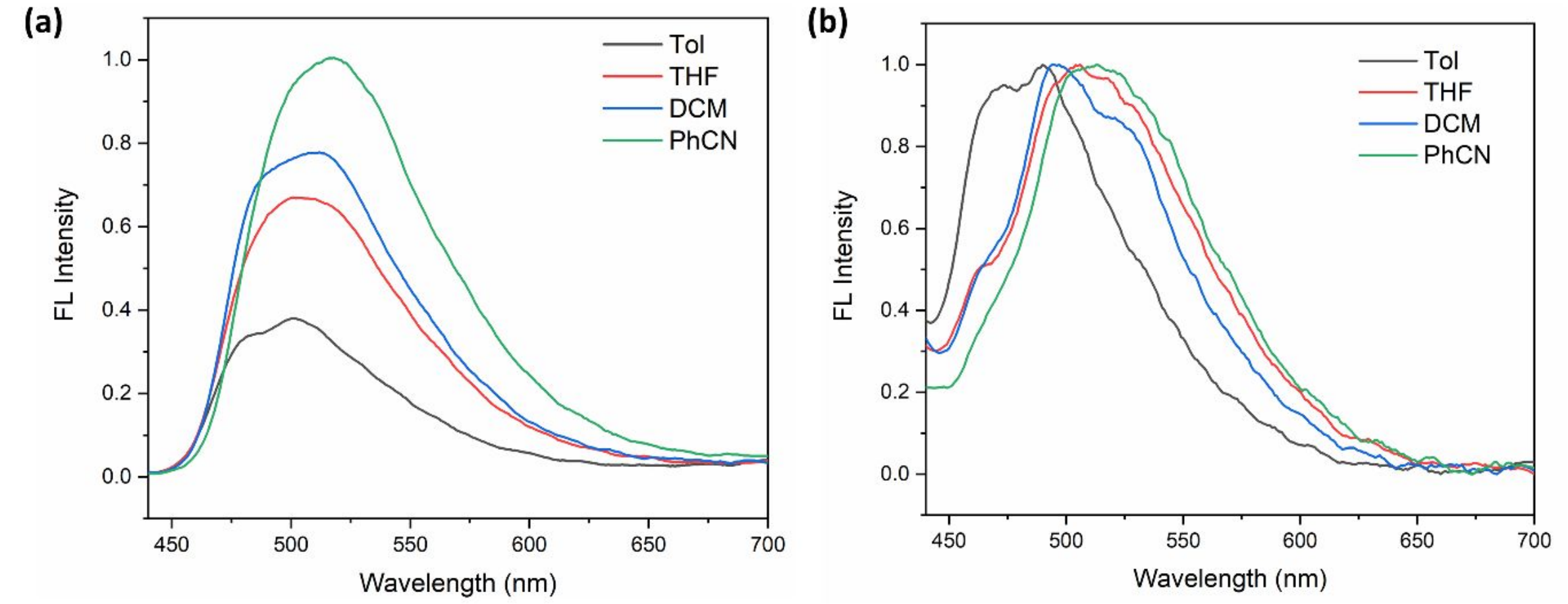

Figure S25. Fluorescence emission spectra of the (a) [Au(TIPS-AntP)Cl] and (b) [Au(TIPS-AntP)$_2$OTf] complexes in different organic solvents spanning a range of polarities at 293 K. Spectra are normalized by absorption at the excitation wavelength (430 nm), OD ~ 0.1

6.1 Quantum yield determination

Fluorescence emission quantum yield of compounds **F**, **G**, **H** and **I** were determined using TIPS-BPEA as reference in degassed toluene. The fluoresce quantum yield of TIPS-BPEA = 0.85 adopted from the previous report.[14] Following equ^n. 1,[15] was considered to determine relative quantum yield of the synthesized compounds with reference to TIPS-BPEA.

$$\phi_{sample} = \phi_{ref} \times \left(\frac{\text{Slope}_{sample}}{\text{Slope}_{ref}}\right) \times \left(\frac{\eta^2{}_{ref}}{\eta^2{}_{sample}}\right) \ldots\ldots\ldots\ldots\ldots(1)$$

To minimize the error associated with the quantum yield determination such as self-quenching, multiple trials are performed in the low concertation regime (with concentration 1-5 μM). Same excitation wavelength and emission slit width maintained for the reference and sample emission.

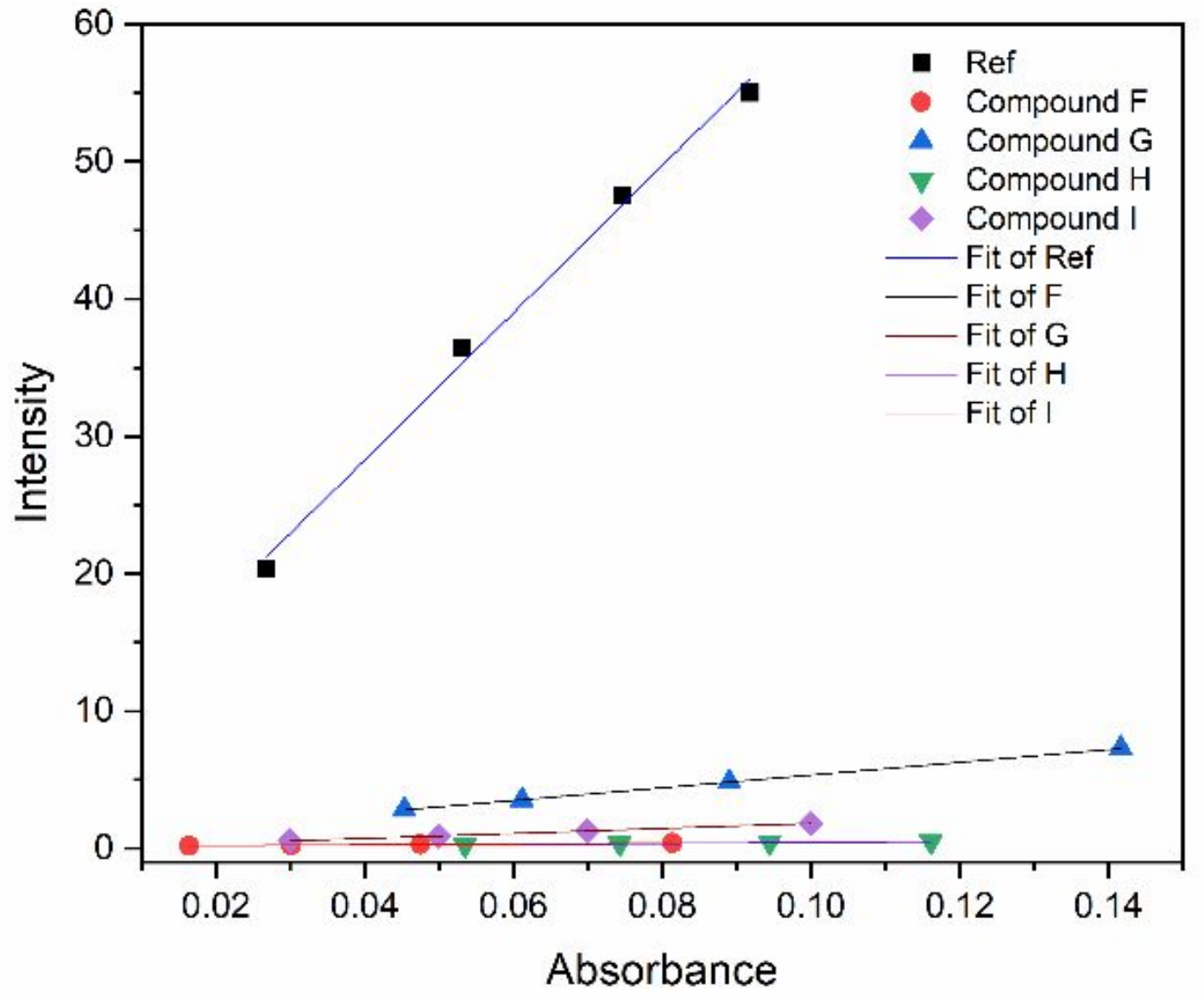

Figure S26. Fluorescence quantum yield calculation of [TIPS-AntP] (compound F), [TIPS-AntPO] (compound G), [Au(TIPS-AntP)Cl] (compound H) and [Au(TIPS-AntP)$_2$OTf] (compound I) using 9,10-Bis(phenylethynyl)anthracene as reference in degassed toluene at room temperature.

7. TD-DFT Calculation

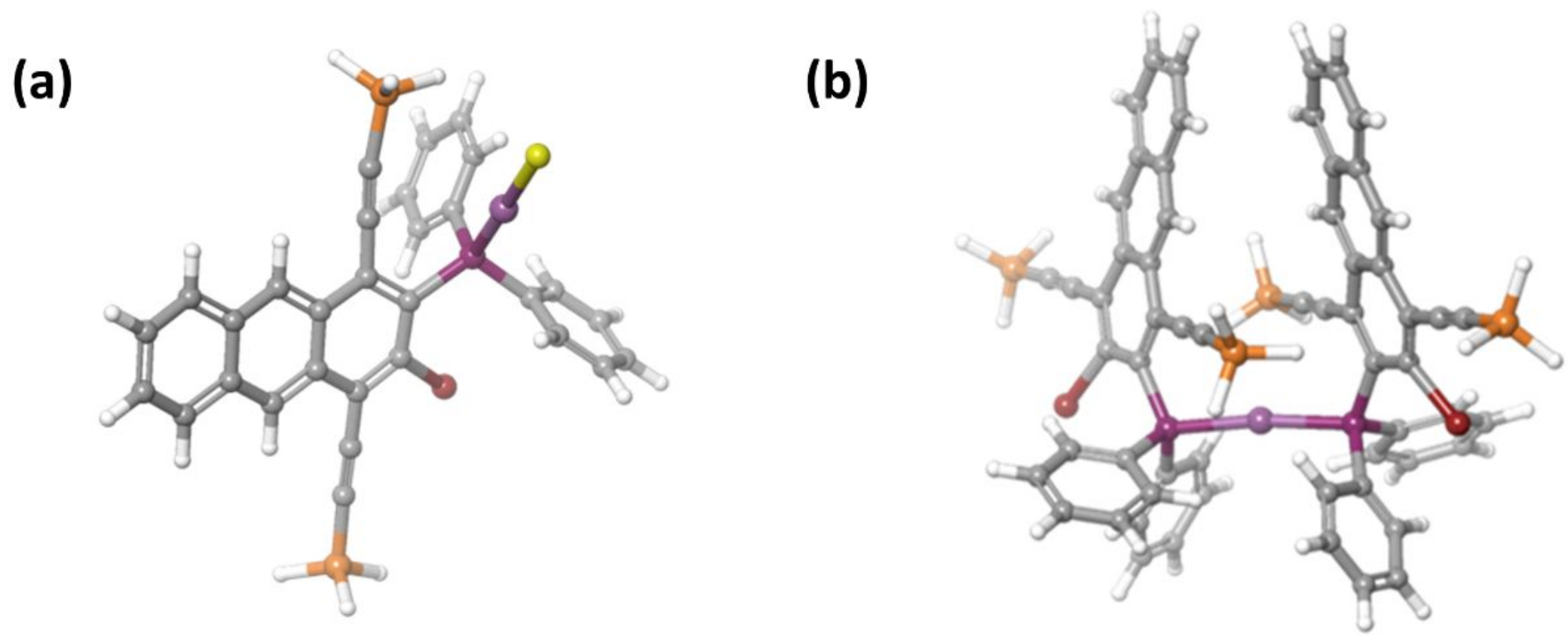

Figure S27. TD-DFT Optimized structure for (a) [Au(TIPS-AntP)Cl] and (b) [Au(TIPS-AntP)$_2$OTf]

Table S2. Singlet excited states of [Au(TIPS-AntP)Cl]

State	Energy (eV)	Wavelength (nm)	Main Transitions	Oscillator Strength (f)	Dipole Moment (D)	Comments
1	2.558	484.71	144 → 145 (0.986), 140 → 145 (–0.106)	0.1106	3.38	Bright state (visible)
2	2.961	418.70	143 → 145 (0.987), 141 → 145 (0.117)	0.0059	0.72	Very weak transition (dark)

3	3.027	409.54	142 → 145 (0.993)	0.0036	0.56	Very weak transition (dark)

141=HOMO-3, 142=HOMO-2, 143=HOMO-1, 144=HOMO, 145=LUMO, 146=LUMO+1, 147=LUMO+2, 148=LUMO+3

Table S3. Triplet excited states of [Au(TIPS-AntP)Cl]

State	Energy (eV)	Wavelength (nm)	Main Transitions	Oscillator Strength (f)	Dipole Moment (D)	Comments
1	1.476	839.99	144 → 145 (0.972), 140 → 147 (0.107), 140 → 148 (0.118)	0.0000	0.00	Triplet, spin-forbidden
2	2.498	496.36	140 → 145 (0.701), 144 → 147 (0.353), 144 → 148 (0.394), others	0.0000	0.00	Triplet, spin-forbidden
3	2.925	423.94	143 → 145 (0.796), 141 → 145 (0.393), others	0.0000	0.00	Triplet, spin-forbidden

141=HOMO-3, 142=HOMO-2, 143=HOMO-1, 144=HOMO, 145=LUMO, 146=LUMO+1, 147=LUMO+2, 148=LUMO+3

Table S4. Singlet excited states of [Au(TIPS-AntP)$_2$OTf]

State	Energy (eV)	Wavelength (nm)	Main Transitions	Oscillator Strength (f)	Dipole Moment (D)	Comments
1	2.40	516.55	HOMO → LUMO (0.724), HOMO → LUMO+1 (–0.665)	0.0998	3.31	Bright state (visible)
2	2.41	514.75	HOMO–1 → LUMO (–0.130), HOMO → LUMO (0.662), HOMO → LUMO+1 (0.728)	0.0017	0.43	Very weak transition (dark)
3	2.47	502.39	260 → 262 (0.977), 261 → 262 (0.159)	0.0378	2.01	Moderate brightness

260=HOMO-1, 261=HOMO, 262=LUMO, 263=LUMO+1

Table S5. Triplet excited states of [Au(TIPS-AntP)$_2$OTf]

State	Energy (eV)	Wavelength (nm)	Main Transitions	Oscillator Strength (f)	Dipole Moment (D)	Comments
T_1	1.429	867.90	261 → 263 (0.797), 260 → 263 (0.492), 261 → 262 (–0.222)	0.0000	0.000	Spin-forbidden (dark state)
T_2	1.443	859.17	260 → 262 (0.780), 261 → 262 (–0.520), 260 → 263 (0.177)	0.0000	0.000	Also spin-forbidden
T_3	2.429	510.45	260 → 262 (0.501), 261 → 262 (0.791), 260 → 263 (0.197)	0.0000	0.000	Triplet with higher energy, still dark

260=HOMO-1, 261=HOMO, 262=LUMO, 263=LUMO+1

Table S6. Calculated energy gaps between key states of [Au(TIPS-AntP)Cl] and [Au(TIPS-AntP)$_2$OTf]

Compound	S_0–S_1 (eV)	S_0–T_1 (eV)	S_0–T_2 (eV)	T_1–S_1 (eV)
[Au(TIPS-AntP)$_2$OTf]	2.400	1.429	1.443	0.971
[Au(TIPS-AntP)Cl]	2.558	1.476	2.498	1.082

8. Transient Absorption Spectroscopy

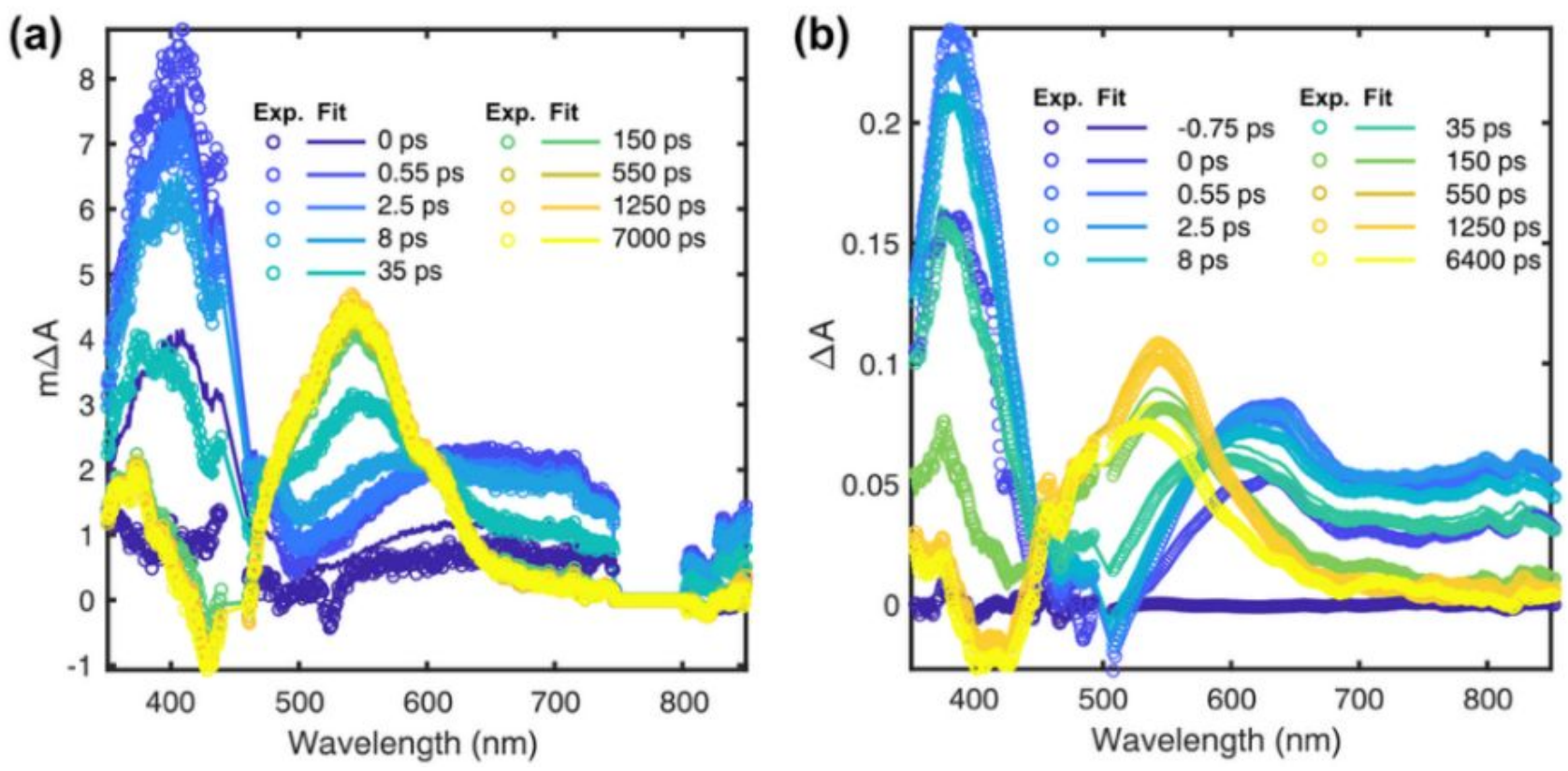

Figure S28. fsTA spectra at selected time points for (a) [Au(TIPS-AntP)Cl] monomer and (b) [Au(TIPS-AntP)$_2$OTf] dimer upon excitation at 430 nm. Solid lines represent reproduced spectra from the global fit.

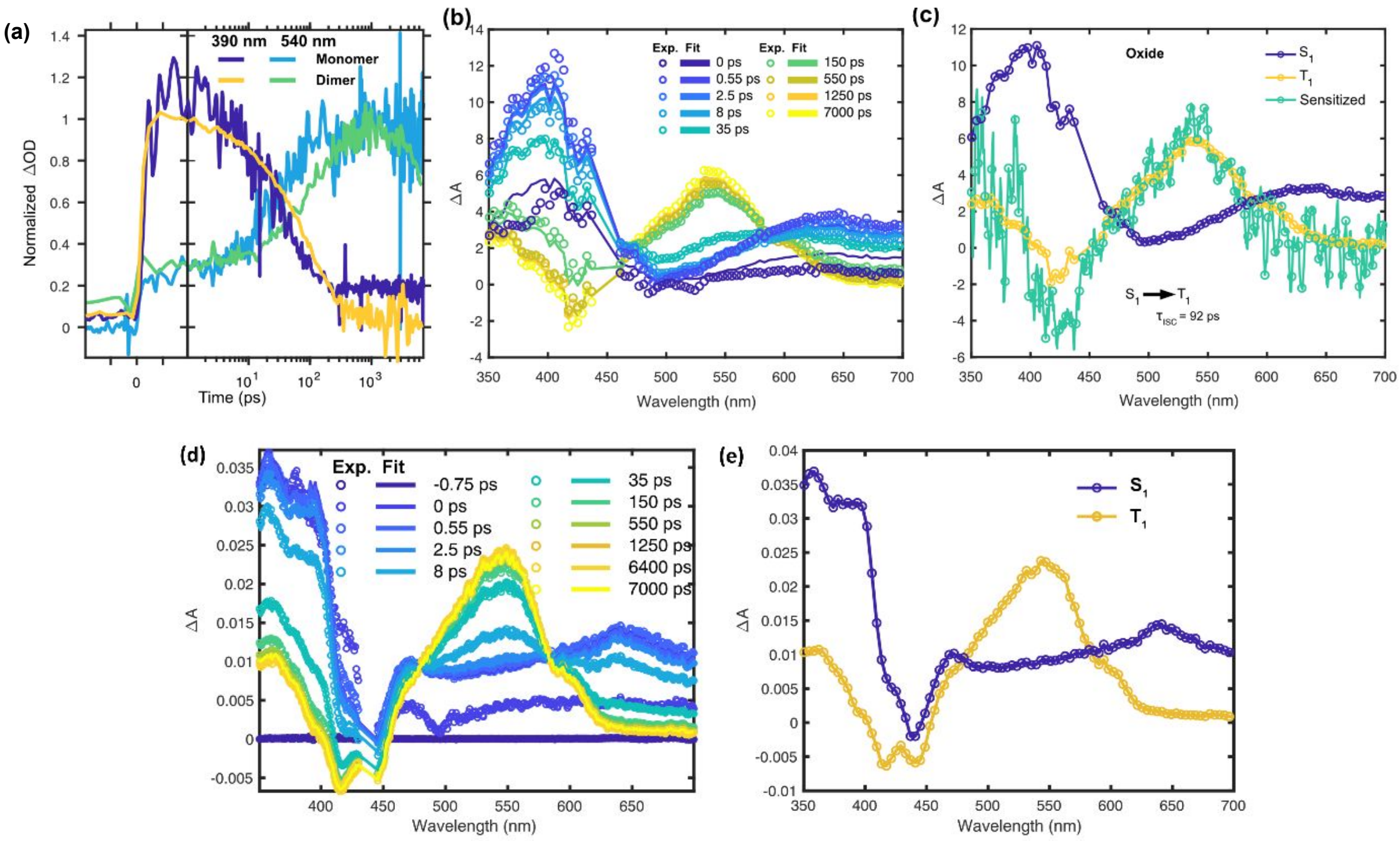

Figure S29. (a) Time evolution of the signal at 390 nm (mostly S_1 PIA) and 540 nm (mostly T_1 PIA) for monomer and dimer complexes. (b) fsTA spectra at selected time points for TIPS-AntPO. Solid lines represent reproduced spectra from the global fit. (c) two component spectra assigned to the singlet and triplet states, as well as the triplet spectra obtained from sensitization experiments for TIPS-AntPO. (d) fsTA spectra at selected time points for TIPS-AntP (e) two component spectra assigned to the singlet and triplet states for TIPS-AntP.

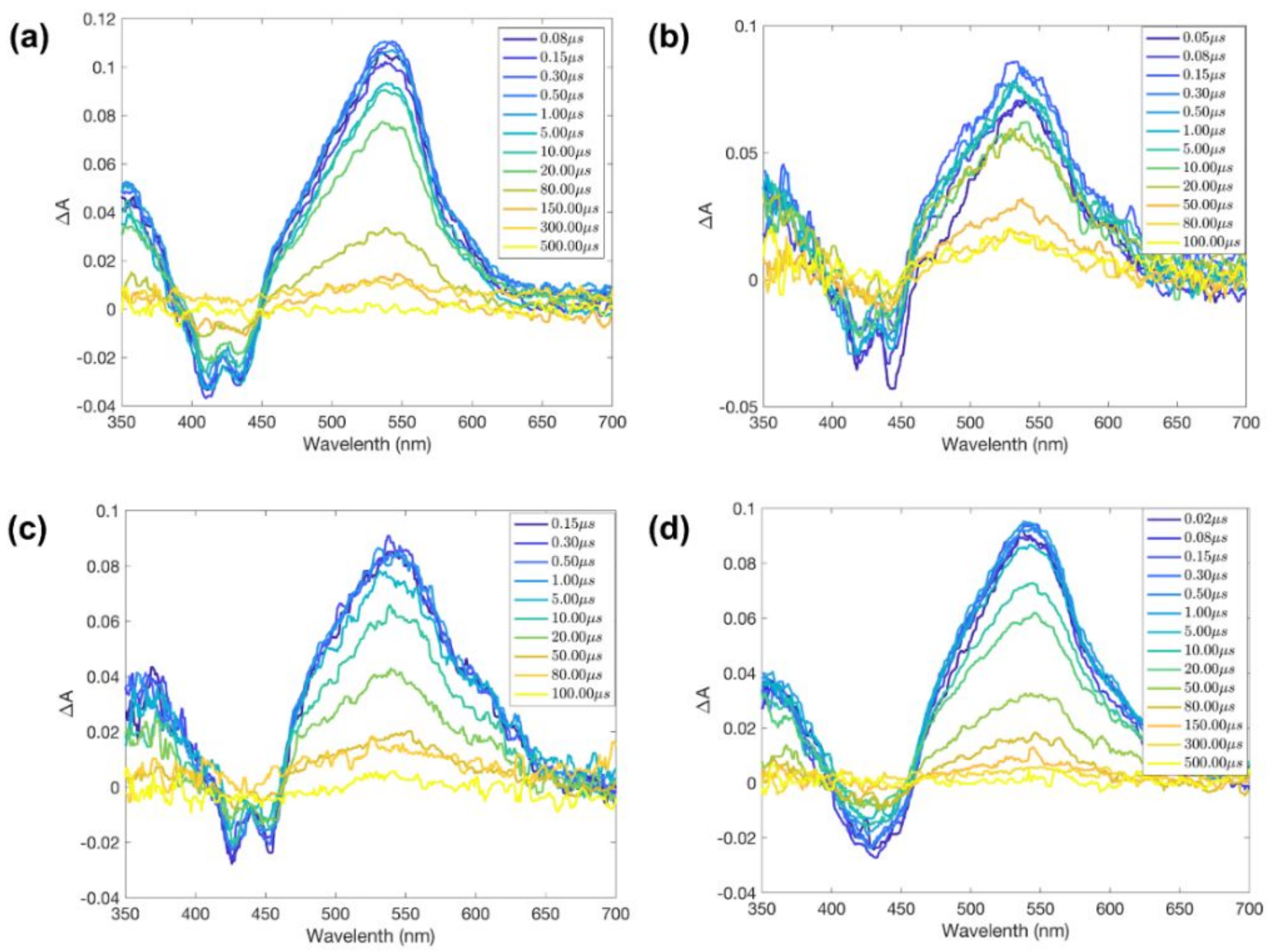

Figure S30. nsTA spectra upon excitation at 430 nm at selected time points for (a) TIPS-AntP, (b) TIPS-AntPO, (c) [Au(TIPS-AntP)Cl] and d) [Au(TIPS-AntP)$_2$OTf].

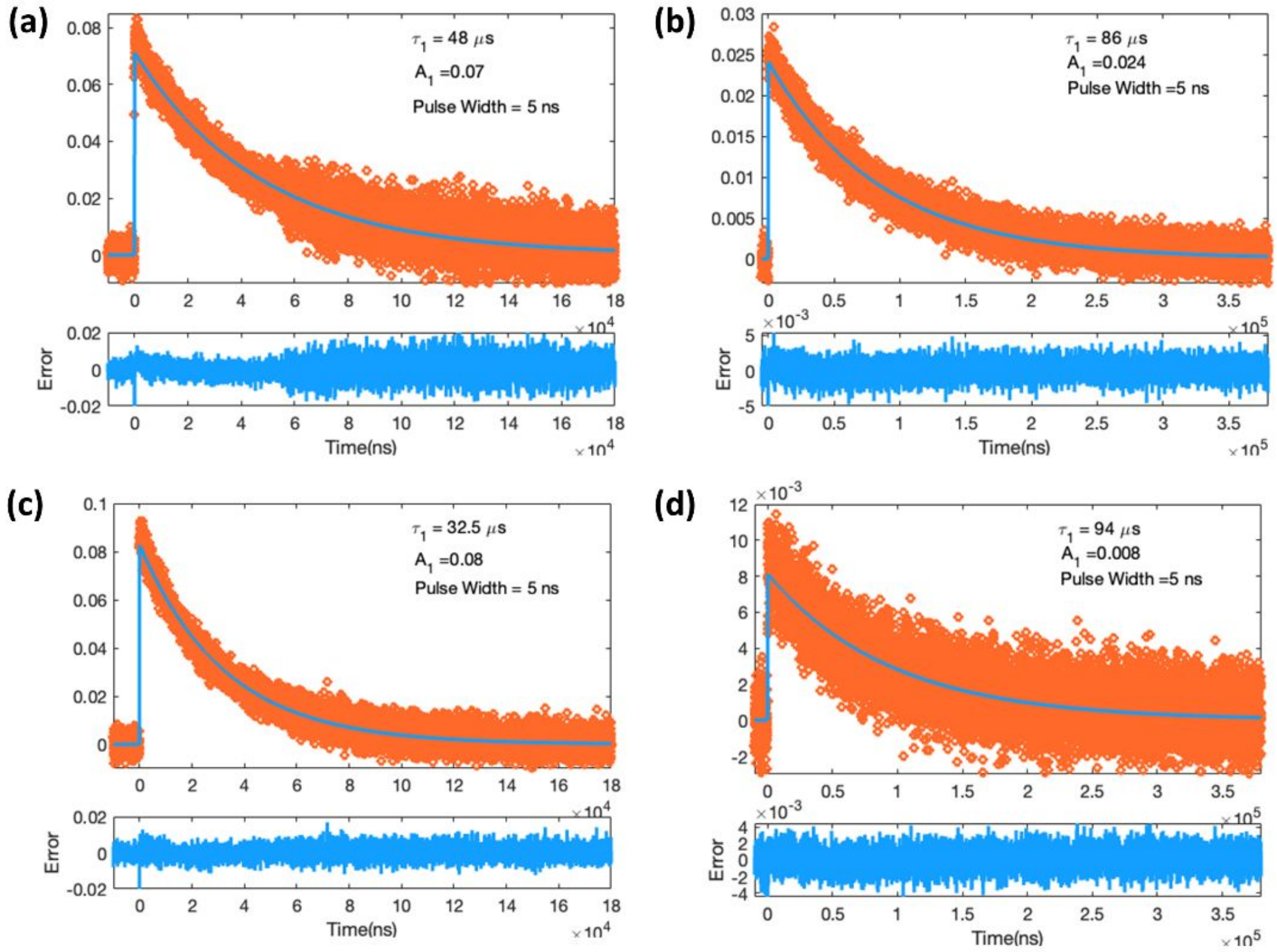

Figure S31. nsTA kinetic decay curves at 540 nm, corresponding to the triplet excited state absorption for a) TIPS-AntPO, b) TIPS-AntP, c) [Au(TIPS-AntP)Cl] and d) [Au(TIPS-AntP)$_2$OTf]. Excitation was at 430 nm with a pulse energy of ~2 mJ/pulse.

9. Room Temperature Phosphorescence

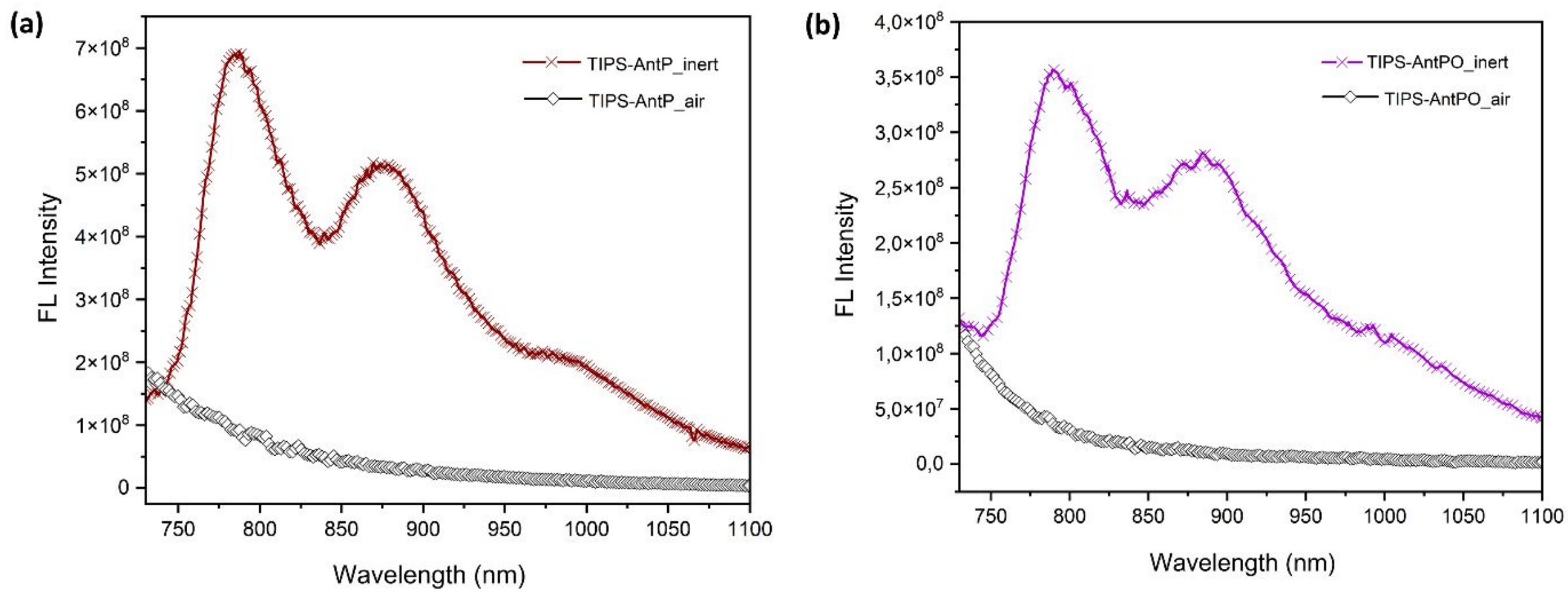

Figure S32. Phosphorescence emission spectra (λ_{exc} = 430 nm) of (a) [TIPS-AntP] and (b) [TIPS-AntPO] recorded in toluene at room temperature under inert atmosphere and under air.

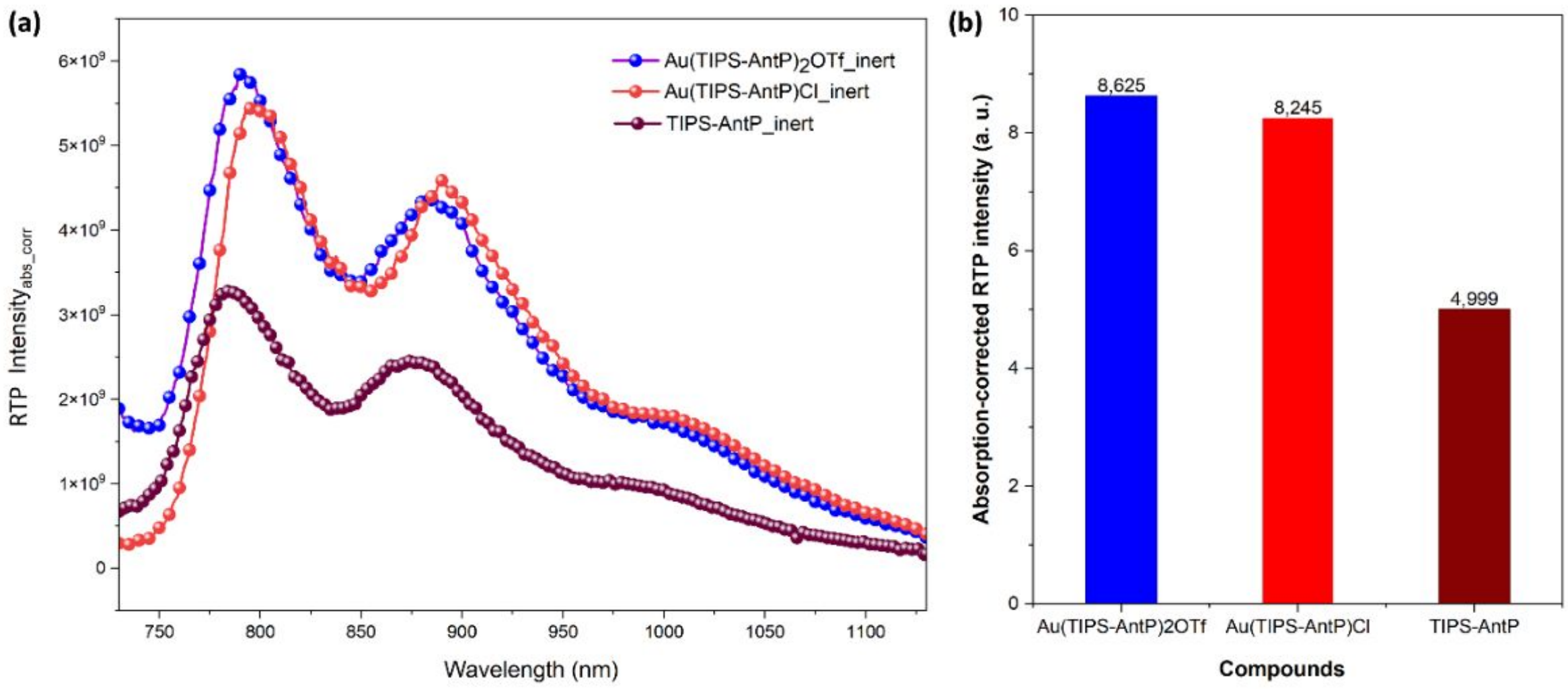

Figure S33. (a) Absorption corrected Phosphorescence emission spectra (λ_{exc} = 430 nm) of [Au(TIPS-AntP)Cl] and [Au(TIPS-AntP)$_2$OTf] in comparison with the free ligand, TIPS-AntP. (b) Integrated RTP intensity normalized to absorption ($1-10^{-A}$).

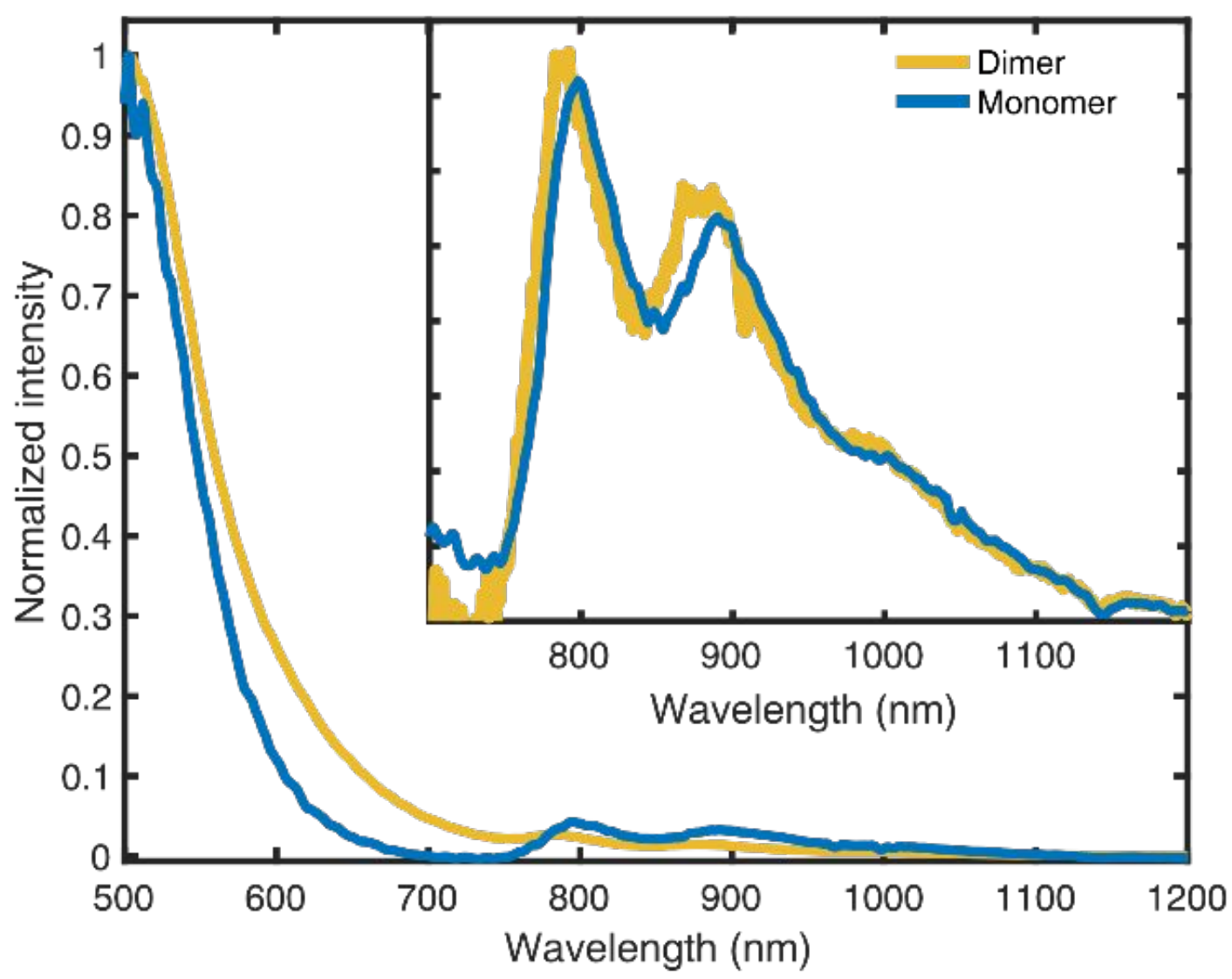

Figure S34. Full range emission spectra of Au(I) monomer and dimer recorded in toluene at room temperature under inert atmosphere

10. Photoinduced Electron Transfer

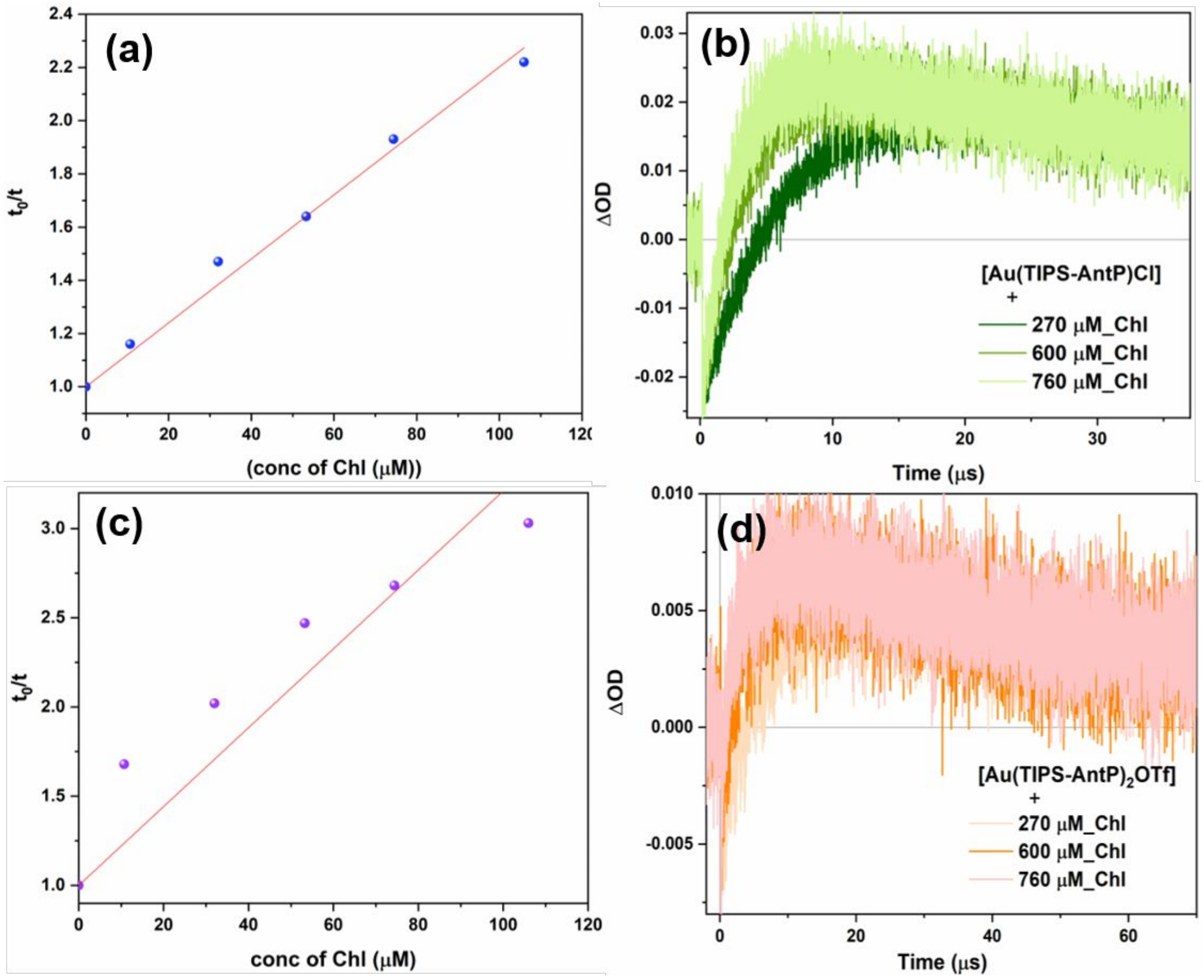

Figure S35. (a) Stern Volmer plot for the electron transfer in the case of monomer [Au(TIPS-AntP)Cl] and chloranil obtained by plotting kinetic traces monitored at 548 nm upon exciting at 470 nm. (b) Rise of the chloranil anion monitored at 440 nm upon adding increasing concentrations of chloranil to a sample of the monomer [Au(TIPS-AntP)Cl] excited at 470 nm. (c) Stern Volmer plot for the electron transfer in the case of the dimer [Au(TIPS-AntP)$_2$OTf] and chloranil obtained by plotting kinetic traces monitored at 548 nm upon exciting at 470 nm. (d) Rise of the chloranil anion monitored at 440 nm upon adding increasing concentrations of chloranil to the dimer [Au(TIPS-AntP)$_2$OTf] excited at 470 nm.

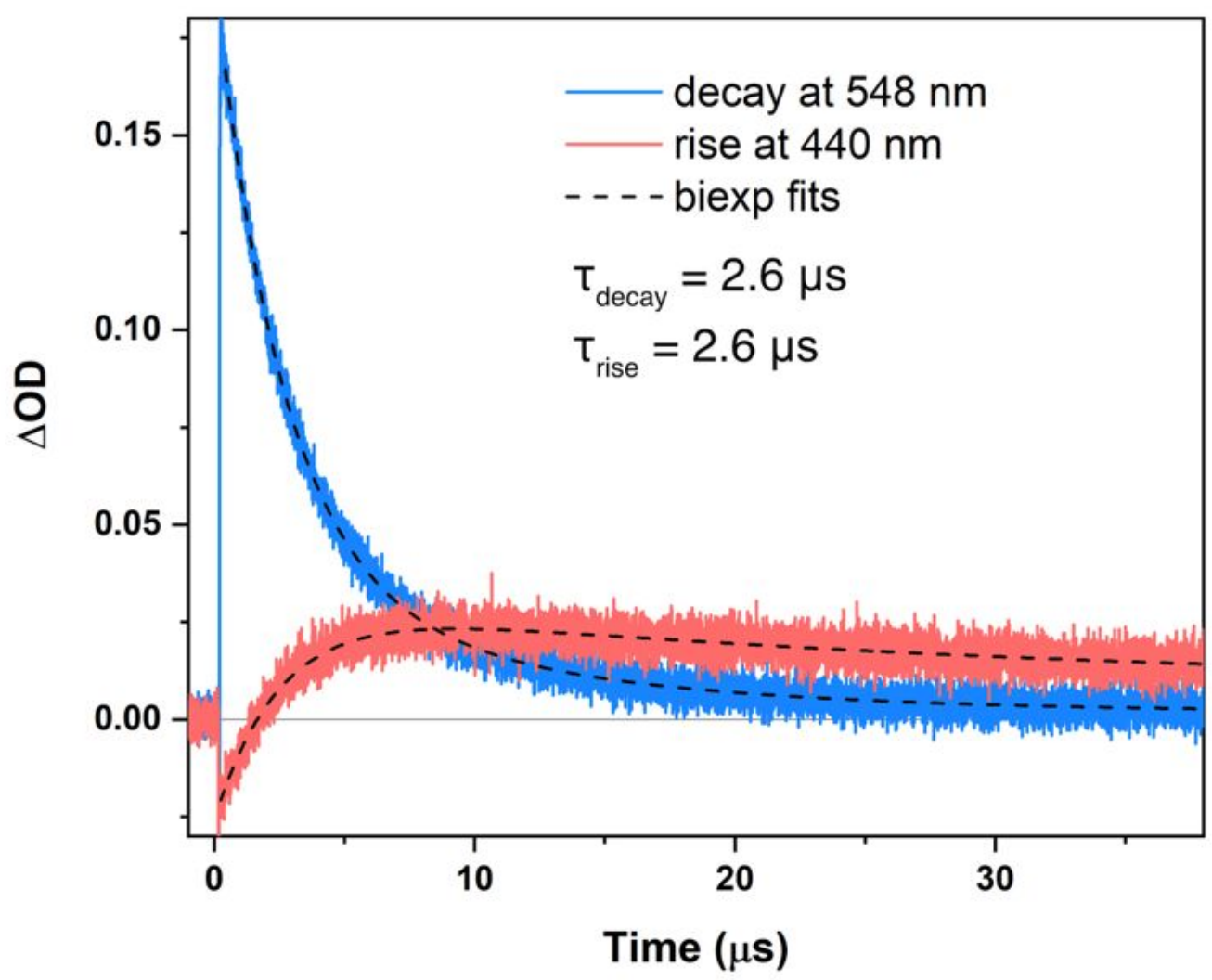

Figure S36. nsTA kinetic decay curves at 548 nm and rise of the chloranil anion monitored at 440 nm upon adding 760 μM of chloranil to the dimeric complex, [Au(TIPS-AntP)$_2$OTf].

11. Electrochemistry

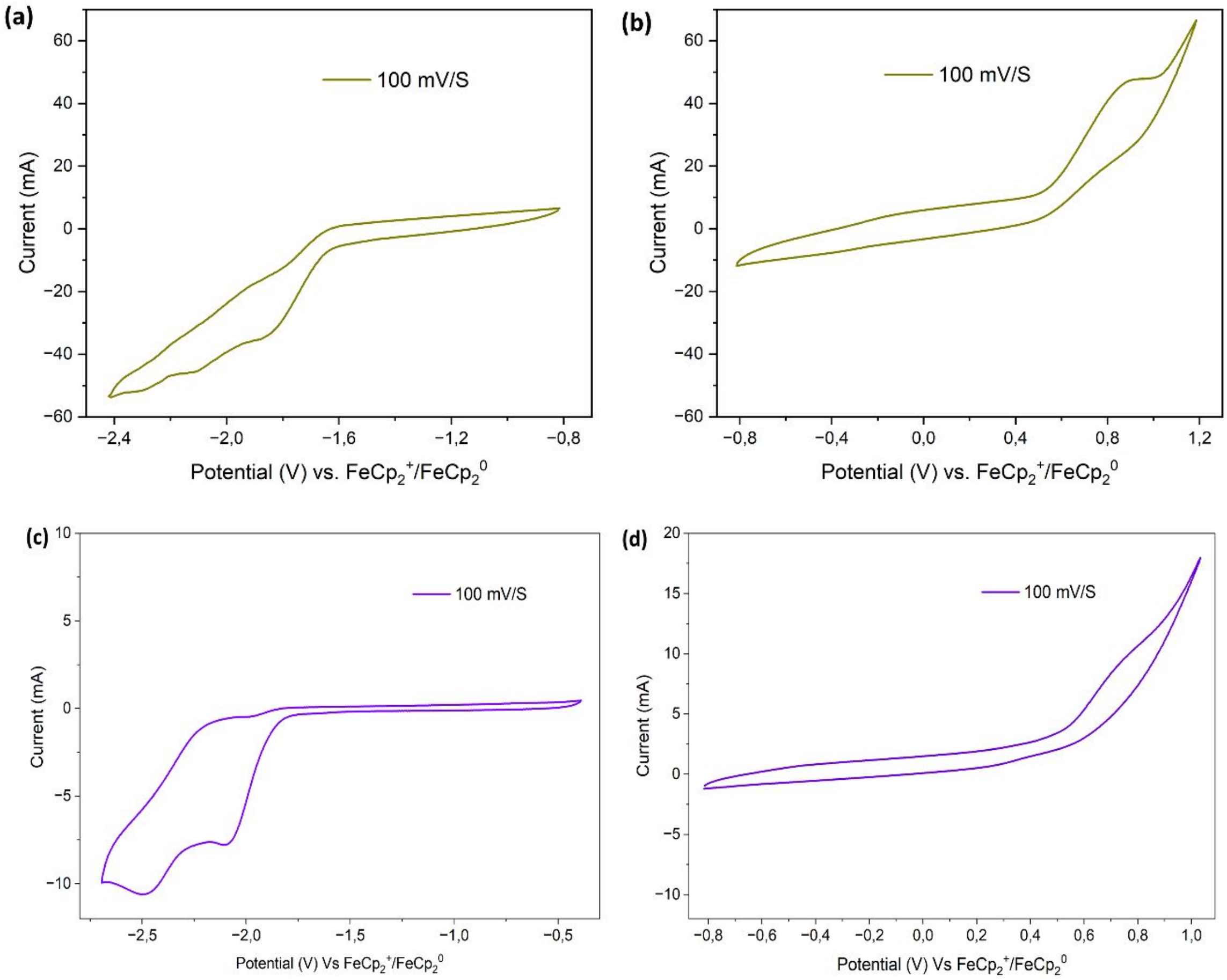

Figure S37. Oxidative *(right)* and reductive *(left)* cyclic voltammogram of (a) & (b) [Au(TIPS-AntP)Cl] and (c) & (d) [Au(TIPS-AntP)$_2$OTf] in deaerated THF. 0.1 M N(nBu)$_4$PF$_6$ was used as an electrolyte and the scan rate was 0.1 Vs^{-1}. The voltammogram was measured in a glovebox and referenced to Fe(Cp*)$_2$.

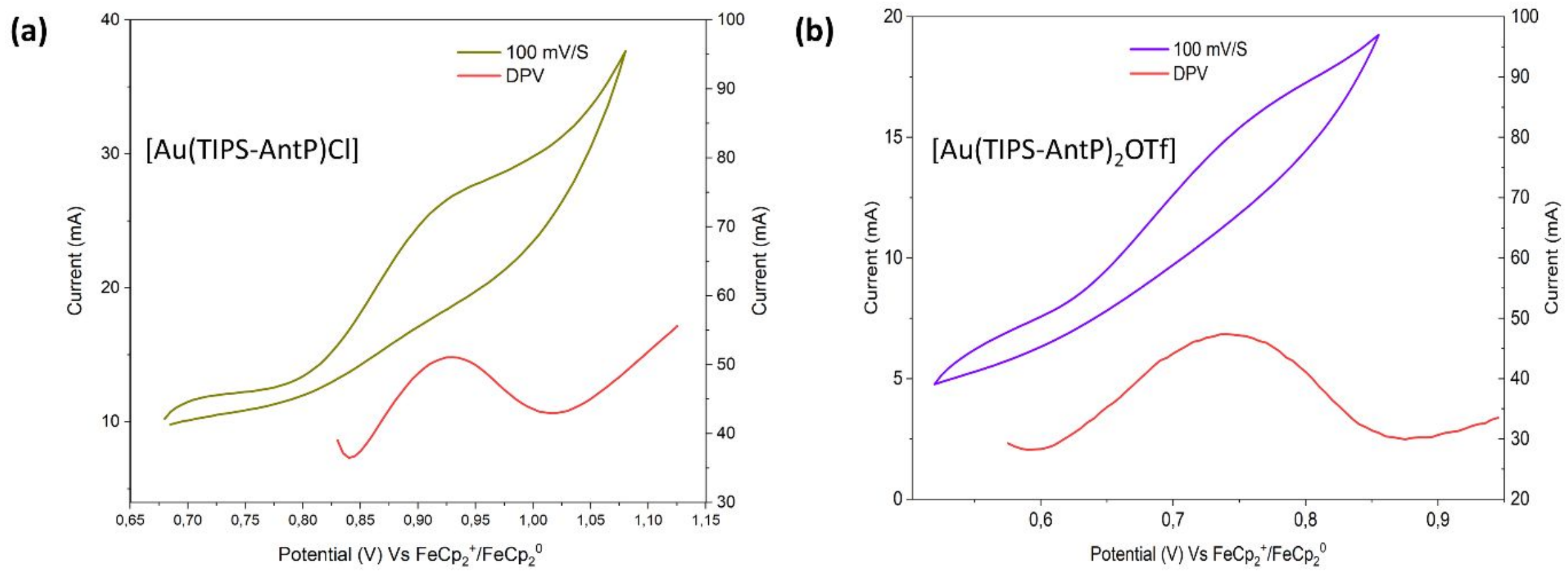

Figure S38. Oxidative cyclic voltammogram (CV) with differential pulse voltammogram (DPV) of (a) [Au(TIPS-AntP)Cl] and (b) [Au(TIPS-AntP)$_2$OTf] in THF using 0.1 M N(nBu)$_4$PF$_6$ as an electrolyte.

12. Singlet oxygen quantum yield determination

Singlet oxygen quantum yield was determined using ZnTPP ($\Phi_{\Delta\mathrm{ref}}$ in $CHCl_3$, 420 nm) = 68% as reference.[16] Then, the quantum yield of singlet oxygen ($\Phi_{\Delta\mathrm{sam}}$) was calculated by Equ^n. 2,[17]

$$\Phi_{\Delta sam} = \Phi_{\Delta ref} \times \frac{1-10^{-A_{ref}}}{1-10^{-A_{sam}}} \times \frac{I_{sam}}{I_{ref}} \times \frac{(\eta_{sam})^2}{(\eta_{ref})^2} \quad \cdots\cdots\cdots\cdots\cdots(2)$$

where, ΦΔ is the quantum yield of singlet oxygen; A is the absorbance; I is the fluorescence intensity; η is the Solvent refractive index. The subscripts "sam" and "ref" denotes sample (Au(I) complexes) and reference (ZnTPP), respectively. Since the sample and reference was taken in same solvent (DCM) then we can omit the refractive index part for calculation.

So for [Au(TIPS-AntP)Cl],

$$\Phi_{\Delta sam} = 68\% \times \frac{1-10^{-0.101}}{1-10^{-0.101}} \times \frac{3.30913}{2.215}$$

$$= 68\% \times 1.0424 = 71\%$$

So for [Au(TIPS-AntP)$_2$OTf],

$$\Phi_{\Delta sam} = 68\% \times \frac{1-10^{-0.101}}{1-10^{-0.102}} \times \frac{3.18613}{2.215}$$

$$= 68\% \times \frac{0.2073}{0.2092} \times \frac{3.18613}{2.215}$$

$$= 68\% \times 1.4246 = 97\%$$

13. Cartesian Coordinates

For [Au(TIPS-AntP)Cl],

C	16.00940	-17.81260	0.57890
C	16.12750	-19.10190	-0.04320
C	17.38200	-19.45920	-0.66950
C	18.46620	-18.59350	-0.57750
C	18.42350	-17.37930	0.17840
C	17.17410	-16.96450	0.66960
C	14.77920	-17.42380	1.12440
C	13.66530	-18.27250	1.09850
C	13.78850	-19.57320	0.48630
C	15.01720	-19.95260	-0.07370
C	12.40960	-17.89130	1.66750
C	11.33830	-18.74730	1.63160
C	11.45950	-20.03380	1.02520
C	12.64800	-20.43520	0.46890
P	19.87050	-16.31180	0.68170
C	19.87960	-14.93950	-0.52430

C	21.45340	-17.20260	0.46230
C	22.41540	-16.79420	-0.47060
C	23.63720	-17.46540	-0.54690
C	23.90150	-18.54540	0.29980
C	22.94830	-18.94240	1.24350
C	21.73380	-18.26490	1.33620
C	20.28460	-13.66460	-0.10170
C	20.33990	-12.61160	-1.01720
C	19.98720	-12.82430	-2.35290
C	19.57770	-14.09330	-2.77500
C	19.52350	-15.15080	-1.86540
Br	20.01560	-19.04060	-1.68730
C	17.01770	-15.68160	1.26180
C	17.46000	-20.67990	-1.39440
C	16.89720	-14.57100	1.75580
C	17.49710	-21.73930	-2.00030
Si	16.99050	-12.92460	2.56680
Si	17.66130	-23.30040	-2.93810
Au	19.79770	-15.62750	2.88740
Cl	19.76700	-14.84280	5.09930
H	14.69250	-16.44900	1.59280
H	15.10790	-20.92560	-0.54490
H	12.32250	-16.91190	2.13060
H	10.38850	-18.45100	2.06710
H	10.59950	-20.69700	1.00810
H	12.74120	-21.41500	0.00770
H	22.21150	-15.96800	-1.14340
H	24.38130	-17.14560	-1.27080
H	24.85160	-19.06770	0.23430
H	23.15570	-19.77090	1.91410
H	21.00600	-18.55870	2.08780
H	20.54540	-13.49610	0.93940
H	20.65100	-11.62580	-0.68430
H	20.02630	-12.00280	-3.06260
H	19.30000	-14.26050	-3.81160
H	19.20880	-16.13410	-2.20060
H	18.38000	-12.43270	2.43620
H	16.06040	-12.00370	1.86430
H	16.59660	-13.05290	3.98580
H	18.98470	-23.32820	-3.60720
H	17.54720	-24.45200	-2.00950
H	16.58520	-23.38410	-3.95630

For [Au(TIPS-AntP)$_2$OTf],

C	15.99750	-19.05240	0.18540
C	16.02790	-17.99890	1.16670
C	17.25850	-17.26490	1.37280
C	18.40470	-17.65660	0.69170
C	18.44010	-18.79740	-0.17050

C	17.22190	-19.42400	-0.48570
C	14.78960	-19.71720	-0.06570
C	13.61550	-19.39420	0.62760
C	13.64940	-18.34140	1.61440
C	14.85620	-17.67000	1.85570
C	12.38170	-20.07530	0.39110
C	11.24990	-19.73030	1.08210
C	11.28160	-18.68650	2.05350
C	12.44720	-18.01380	2.31250
P	19.96890	-19.62620	-0.82770
C	20.23980	-19.02690	-2.53140
C	21.47040	-19.20350	0.13780
C	21.56330	-19.64510	1.46720
C	22.73150	-19.43620	2.19610
C	23.83050	-18.81170	1.60000
C	23.74950	-18.39200	0.27130
C	22.57500	-18.58160	-0.45950
C	19.79390	-17.76350	-2.94560
C	20.05020	-17.32790	-4.24540
C	20.75160	-18.14360	-5.13480
C	21.19470	-19.40380	-4.72730
C	20.93640	-19.84970	-3.43170
Br	19.95100	-16.46170	0.82400
C	17.14410	-20.39670	-1.52550
C	17.25740	-16.13940	2.24230
C	16.99930	-21.14720	-2.47830
C	17.24390	-15.18190	2.99660
Si	16.76900	-22.20640	-3.95850
Si	17.32570	-13.72150	4.10370
Au	19.90390	-22.01500	-0.67120
C	16.14740	-25.04830	-0.25120
C	15.79450	-25.86420	-1.38430
C	16.84890	-26.46630	-2.17290
C	18.17380	-26.17740	-1.86780
C	18.54510	-25.22380	-0.86900
C	17.53740	-24.72930	-0.02200
C	15.13120	-24.54320	0.57100
C	13.77880	-24.79480	0.30430
C	13.42630	-25.59280	-0.84590
C	14.44510	-26.10890	-1.65870
C	12.73380	-24.28480	1.13450
C	11.42110	-24.54930	0.84560
C	11.07340	-25.33510	-0.29190
C	12.04610	-25.84090	-1.11320
P	20.20060	-24.39110	-0.68710
C	21.02430	-25.11180	0.77330
C	21.32390	-24.67510	-2.11000
C	22.51240	-25.40550	-1.97860
C	23.39040	-25.51410	-3.05830
C	23.09150	-24.90320	-4.27690

C	21.91020	-24.16750	-4.41020
C	21.03860	-24.04020	-3.32990
C	20.81200	-26.44760	1.14580
C	21.50370	-26.98720	2.23140
C	22.40880	-26.20300	2.95000
C	22.61720	-24.87090	2.58430
C	21.92650	-24.32340	1.50340
Br	19.53280	-27.26470	-2.76280
C	17.86380	-23.91770	1.10420
C	16.50270	-27.36050	-3.22420
C	18.11330	-23.28430	2.11760
C	16.18460	-28.10510	-4.13640
Si	18.53320	-22.38940	3.66130
Si	15.80280	-29.25070	-5.51750
H	14.76490	-20.51060	-0.80470
H	14.88430	-16.87120	2.58860
H	12.35900	-20.87230	-0.34680
H	10.31630	-20.25170	0.89060
H	10.37270	-18.42710	2.58830
H	12.47580	-17.21870	3.05200
H	20.72180	-20.14540	1.93850
H	22.78670	-19.76690	3.22930
H	24.74450	-18.65760	2.16610
H	24.60110	-17.90980	-0.20090
H	22.52160	-18.23710	-1.48720
H	19.25260	-17.11990	-2.25990
H	19.70260	-16.34900	-4.56200
H	20.95040	-17.79930	-6.14580
H	21.73910	-20.04100	-5.41880
H	21.27620	-20.83430	-3.11930
H	16.42570	-21.34930	-5.11800
H	15.68200	-23.17980	-3.70160
H	18.03100	-22.93340	-4.23770
H	18.29090	-12.74610	3.54190
H	15.98170	-13.10340	4.20220
H	17.77600	-14.15670	5.44840
H	15.39670	-23.93500	1.42860
H	14.18520	-26.72590	-2.51200
H	13.00130	-23.68610	2.00040
H	10.63420	-24.16160	1.48590
H	10.02680	-25.53350	-0.50330
H	11.78340	-26.44190	-1.97890
H	22.75320	-25.89740	-1.04240
H	24.30930	-26.08260	-2.94490
H	23.77510	-24.99580	-5.11610
H	21.66960	-23.68850	-5.35540
H	20.12850	-23.45640	-3.44110
H	20.11320	-27.06740	0.59160
H	21.33500	-28.02270	2.51300
H	22.94660	-26.62720	3.79280

H	23.31720	-24.25560	3.14190
H	22.08800	-23.28460	1.22730
H	17.57270	-21.27940	3.86530
H	18.47450	-23.33070	4.80390
H	19.90550	-21.84020	3.53860
H	17.03450	-29.47250	-6.31230
H	15.31650	-30.53820	-4.96610
H	14.75420	-28.64630	-6.37510

14. Reference

1. Gray, V.; Moth-Poulsen, K.; Dreos, A.; Erhart, P.; Albinsson, B.; Abrahamsson, M. Loss Channels in Triplet–Triplet Annihilation Photon Upconversion: Importance of Annihilator Singlet and Triplet Surface Shapes. *Phys. Chem. Chem. Phys*. **2017**, *19*, 10931–10939.
2. Dolomanov, O. V.; Bourhis, L. J.; Gildea, R. J.; Howard, J. A. K.; Puschmann, H. OLEX2: A Complete Structure Solution, Refinement and Analysis Program. *J. Appl. Cryst*. **2009**, *42*, 339–341.
3. Sheldrick, G. M. SHELXT–Integrated Space-Group and Crystal-Structure Determination. *Acta Cryst. A* **2015**, *71*, 3–8.
4. Bochevarov, A. D.; Harder, E.; Hughes, T. F.; Greenwood, J. R.; Braden, D. A.; Philipp, D. M.; Rinaldo, D.; Halls, M. D.; Zhang, J.; Friesner, R. A. Jaguar: A High-Performance Quantum Chemistry Software Program with Strengths in Life and Materials Sciences. *Int. J. Quantum Chem*. **2013**, *113*, 2110–2142.
5. Becke, A. D. Density-Functional Exchange-Energy Approximation with Correct Asymptotic Behavior. *Phys. Rev. A* **1988**, *38*, 3098–3100.
6. Lee, C.; Yang, W.; Parr, R. G. Development of the Colle–Salvetti Correlation-Energy Formula into a Functional of the Electron Density. *Phys. Rev. B* **1988**, *37*, 785–789.
7. Becke, A. D. Density-Functional Thermochemistry. III. The Role of Exact Exchange. *J. Chem. Phys*. **1993**, *98*, 5648–5652.
8. Grimme, S.; Antony, J.; Ehrlich, S.; Krieg, H. A Consistent and Accurate Ab Initio Parametrization of Density Functional Dispersion Correction (DFT-D) for the 94 Elements H–Pu. *J. Chem. Phys.* **2010**, *132*, 154104.
9. Grimme, S.; Ehrlich, S.; Goerigk, L. Effect of the Damping Function in Dispersion-Corrected Density Functional Theory. *J. Comput. Chem.* **2011**, *32*, 1456–1465.
10. Cao, Y.; Hughes, T.; Giesen, D.; Halls, M. D.; Goldberg, A.; Vadicherla, T. R.; Sastry, M.; Patel, B.; Sherman, W.; Weisman, A. L.; Friesner, R. A. Highly Efficient Implementation of Pseudospectral Time-Dependent Density-Functional Theory for the Calculation of Excitation Energies of Large Molecules. *J. Comput. Chem*. **2016**, *37*, 1425–1441.
11. Ko, C.; Malick, D. K.; Braden, D. A.; Friesner, R. A.; Martínez, T. J. Pseudospectral Time-Dependent Density Functional Theory. *J. Chem. Phys*. **2008**, *128*, 104103.
12. Mao, Z.; Huang, A.; Ma, L.; Zhang, M. Visible Light Enabled [4+2] Annulation Reactions for Anthracenone-Furans from 2,3-Dibromonaphthoquinone and Phenylbenzofurans. *RSC Adv*. **2021**, *11*, 38235–38238.

13. Chuong, R.; Luck, K. A.; Luck, R. L.; Nguyen, L. P.; Phan, D.; Pignotti, L. R.; Urnezius, E.; Valente, E. J. Ditopic Ligands Featuring [P,S], [P,P] or [P,B] Chelating Pockets Housed on a Protected o-Hydroquinone Core. *J. Organomet. Chem*. **2013**, *724*, 45–50.
15. Nishimura, N.; Gray, V.; Allardice, J. R.; Beljonne, D.; Rao, A. Photon Upconversion from Near-Infrared to Blue Light with TIPS-Anthracene as an Efficient Triplet–Triplet Annihilator. *ACS Mater. Lett.* **2019**, *1*, 660–664.
16. Levitus, M. Tutorial: Measurement of Fluorescence Spectra and Determination of Relative Fluorescence Quantum Yields of Transparent Samples. *Methods Appl. Fluoresc*. **2020**, *8*, 033001.
17. O'Neill, J. S.; Boyle, N. M.; Passos, T. M.; Heintz, K.; Browne, W. R.; Quilty, B.; Pryce, M. T. Photophysical and Electrochemical Properties of meso-Tetrathien-2′-yl Porphyrins Compared to meso-Tetraphenylporphyrin. *J. Photochem. Photobiol., A* **2023**, *438*, 114573.
18. Chen, K.-K.; Guo, S.; Liu, H.; Li, X.; Zhang, Z.-M.; Lu, T.-B. Strong Visible-Light-Absorbing Cuprous Sensitizers for Dramatically Boosting Photocatalysis. *Angew. Chem., Int. Ed*. **2020**, *59*, 12951–12957.
